# Genome organization and chromatin analysis identify transcriptional downregulation of insulin-like growth factor signaling as a hallmark of aging in developing B cells

**DOI:** 10.1186/s13059-018-1489-y

**Published:** 2018-09-05

**Authors:** Hashem Koohy, Daniel J. Bolland, Louise S. Matheson, Stefan Schoenfelder, Claudia Stellato, Andrew Dimond, Csilla Várnai, Peter Chovanec, Tamara Chessa, Jeremy Denizot, Raquel Manzano Garcia, Steven W. Wingett, Paula Freire-Pritchett, Takashi Nagano, Phillip Hawkins, Len Stephens, Sarah Elderkin, Mikhail Spivakov, Peter Fraser, Anne E. Corcoran, Patrick D. Varga-Weisz

**Affiliations:** 1 Nuclear Dynamics Programme, Babraham Institute, Cambridge, UK; 2 Human Immunology Unit, MRC Weatherall Institute of Molecular Medicine, University of Oxford, Oxford, UK; 3 Laboratory of Lymphocyte Signalling and Development, Babraham Institute, Cambridge, UK; 4 Signalling, Babraham Institute, Cambridge, UK; 5 Present address: Université Clermont Auvergne, Inserm U1071, M2iSH, USC-INRA 2018, F-, 63000 Clermont-Ferrand, France; 6 Bioinformatics, Babraham Institute, Cambridge, UK; 7 Division of Cell Biology, Medical Research Council Laboratory of Molecular Biology, Cambridge, CB2 0QH UK; 8 Department of Biological Science, Florida State University, Tallahassee, FL, USA; 9 School of Biological Sciences, University of Essex, Colchester, UK; 10 Functional Gene Control Group, MRC London Institute of Medical Sciences (LMS), Du Cane Road, London, W12 0NN UK

## Abstract

**Background:**

Aging is characterized by loss of function of the adaptive immune system, but the underlying causes are poorly understood. To assess the molecular effects of aging on B cell development, we profiled gene expression and chromatin features genome-wide, including histone modifications and chromosome conformation, in bone marrow pro-B and pre-B cells from young and aged mice.

**Results:**

Our analysis reveals that the expression levels of most genes are generally preserved in B cell precursors isolated from aged compared with young mice. Nonetheless, age-specific expression changes are observed at numerous genes, including microRNA encoding genes. Importantly, these changes are underpinned by multi-layered alterations in chromatin structure, including chromatin accessibility, histone modifications, long-range promoter interactions, and nuclear compartmentalization. Previous work has shown that differentiation is linked to changes in promoter-regulatory element interactions. We find that aging in B cell precursors is accompanied by rewiring of such interactions. We identify transcriptional downregulation of components of the insulin-like growth factor signaling pathway, in particular downregulation of Irs1 and upregulation of Let-7 microRNA expression, as a signature of the aged phenotype. These changes in expression are associated with specific alterations in H3K27me3 occupancy, suggesting that Polycomb-mediated repression plays a role in precursor B cell aging.

**Conclusions:**

Changes in chromatin and 3D genome organization play an important role in shaping the altered gene expression profile of aged precursor B cells. Components of the insulin-like growth factor signaling pathways are key targets of epigenetic regulation in aging in bone marrow B cell precursors.

**Electronic supplementary material:**

The online version of this article (10.1186/s13059-018-1489-y) contains supplementary material, which is available to authorized users.

## Background

Old age is accompanied by increased frailty including a breakdown in functionality of the adaptive immune system mediated by B and T lymphocytes [1]. This results in refractory responses to vaccination, loss of previously established immunity, and substantial increases in susceptibility to infection. Unravelling the molecular changes and mechanisms underlying aging phenotypes is thus an important task for biology. The B cell population is a critical pillar of adaptive immunity, involved in generating protective antibodies, presenting antigens, and regulating immune responses. B cells develop continuously in the bone marrow from hematopoietic stem cells through several precursor stages, including pro-B cells, where immunoglobulin heavy chain (IgH) recombination occurs, followed by pre-B cells in which the immunoglobulin light chains (IgK or IgL) recombine. Inherent inefficiencies in the recombination process lead to substantial cell loss at each stage. To provide adequate numbers of B cells to ensure a diverse antibody repertoire, recombination events alternate with proliferative expansion at each stage to restore depleted B cell numbers. Pro-B cell expansion is controlled by the interleukin-7 receptor (IL7R) [2], potentiated by the insulin-like growth factor 1 (IGF1) receptor [3], while progression to the pre-B cell stage is characterized by signaling through both the IL7R and the pre-B cell receptor (pre-BCR) which is composed of the productively recombined IgH and the invariant surrogate light chain (SL) [4]. Thereafter, the pre-BCR assumes control of both pre-B cell proliferation and IgK recombination [5, 6]. This pro-B to pre-B transition also requires IGF1 signaling [7]. The size of precursor B cell subsets and the primary antibody repertoire are reduced during aging (reviewed in [8]), which, together with defects in maturation of the antigen-responsive repertoire, substantially reduces the antibody response to infection during aging. In particular, the size of the pre-B cell pool is reduced in the aged mouse, indicating that aging-specific defects arise early in B cell development [9]. In vivo labeling experiments show that the progression of B cell progenitors through the pro- and pre-B cell stages is also diminished with age [10–12]. There is evidence of both B cell-intrinsic defects (e.g. [13]) as well as defects in the stromal cell compartment [10], which supports developmental progression, but the underlying causes of these changes remain to be elucidated (reviewed in [8]). In particular, the nature and extent of changes in gene expression in aged B cell precursors are unknown.

The advent of new technologies in functional genomics enables illumination of the changes in B cell development that occur during aging genome-wide. Recently, application of these technologies to aging human T cells has provided profound insight into widespread epigenetic changes that impair the function of closely related lymphocytes in the adaptive immune system. In particular, aging CD8+ T cells lose chromatin accessibility at promoters, which may compromise their metabolic state [14]. Comparison of aging human lymphocyte populations in peripheral blood has revealed concordant downregulation of chromatin accessibility and gene expression of components of the IL7R signaling pathway in CD8+, but not CD4+ T cells, suggesting that epigenetic dysfunction in shared pathways in aging requires careful unravelling [15].

Here we present an integrated analysis of the transcriptome, epigenetic landscape, and higher-order chromatin structure in young and aged pro- and pre-B cells from mouse bone marrow. We show that aging affects the expression of a limited number of genes, in particular key components of the insulin-like growth factor (IGF) signaling pathway. These alterations in the transcriptome are accompanied by perturbations of multiple regulatory layers affecting transcriptional and post-transcriptional mechanisms, including microRNAs (miRNAs) and polycomb-mediated epigenetic regulation.

## Results

### Gene expression changes in B cell precursors in aged mice

**Fig. 1:**
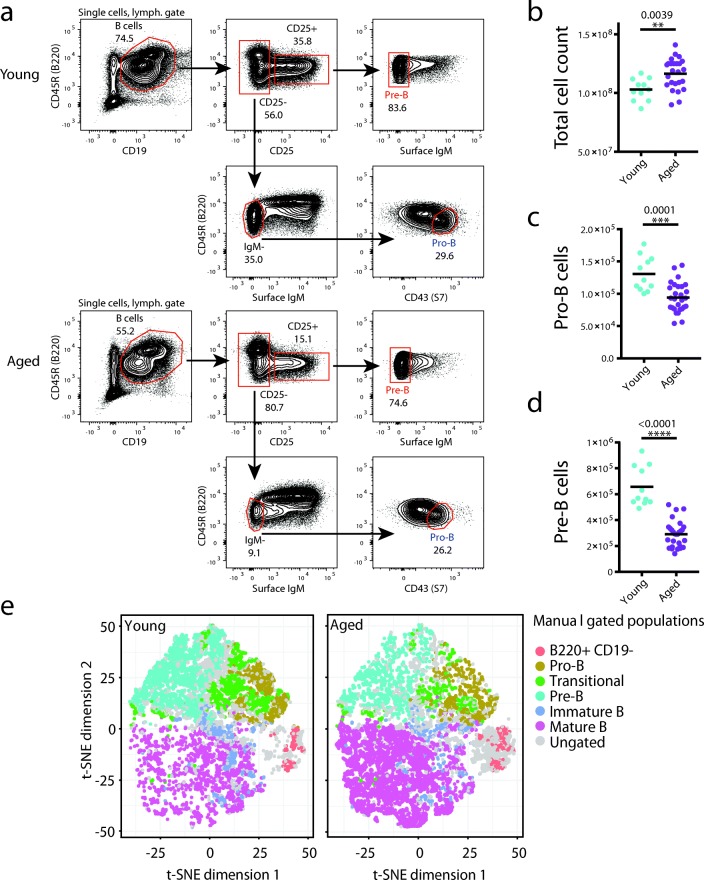
Reduction in average pro-B and pre-B cell numbers upon aging. **a** Representative *flow cytometry plots* showing the gating strategy used to isolate pro- and pre-B cells from young and aged bone marrow following depletion of non-B cells. Numbers indicate percentage of cells in the gate. **b**–**d** Average total number of cells (**b**) and numbers of flow-sorted pro-B (**c**) and pre-B (**d**) cells obtained from the bone marrow of both tibias and femurs of young and aged mice. Each point represents the average number of cells per mouse from a single flow sort comprising cohorts of 12–15 mice. Differences were tested for significance using an unpaired t test. **e** t-SNE analysis based on flow cytometry data shows changes in bone marrow derived B cell precursor populations between young (3 months) and aged mice (19–22 months)

Using multi-parameter flow cytometry analysis of the bone marrow B cell compartment, we found substantial changes in the B cell precursor pools in aged mice (Fig. 1). Our analysis revealed a roughly twofold reduction in pro-B (Fig. 1c) and an almost threefold reduction in pre-B cell numbers (Fig. 1d) in the bone marrow of aged (19–22 months) compared with young mice (three months), consistent with previous reports [9, 16]. In contrast, we found an increase in recirculating mature B cells in the bone marrow of aged mice (Fig. 1e).

**Fig. 2:**
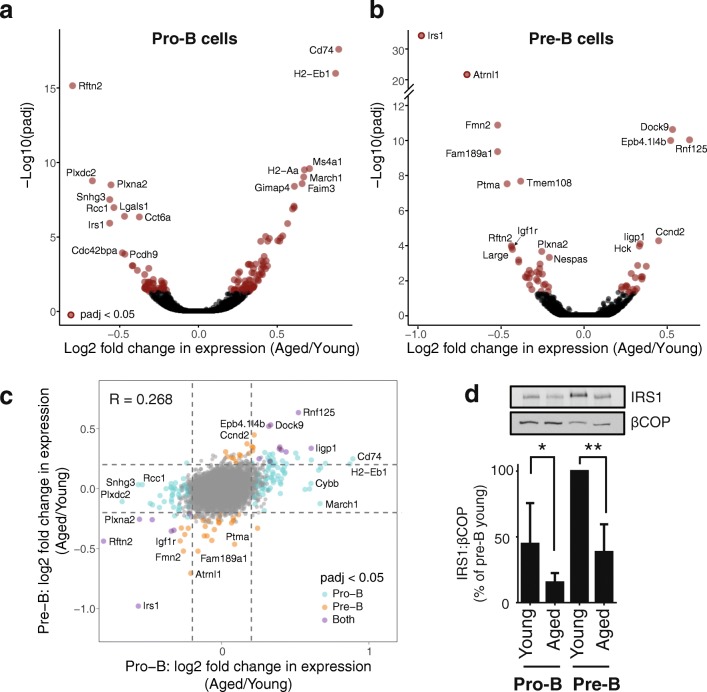
Gene expression changes upon aging in pro- and pre-B cells. **a**, **b** *Volcano plots* of total RNA (depleted of rRNA) expression changes in aged pro-B (**a**) and pre-B cells (**b**). Names of selected genes showing highly significant changes are indicated. Padj: FDR adjusted *p* value. **c** Comparison of fold change in gene expression upon aging between pre-B and pro-B cells, showing a similar trend at both developmental stages; Pearson correlation coefficient is indicated. Genes with a significant (FDR adjusted *p* value < 0.05) age-related change in expression are indicated by a larger point and are colored based on whether this change is observed in pro-B, pre-B cells or both. *Dashed lines* indicate a log2 fold change of ± 0.2. **d** *Western blot* showing changes in IRS1 protein levels between young and aged pro- and pre-B cells, relative to a loading control (βCOP). Protein equivalent of 1 × 10^6^ cells per lane. *Top*: A representative western blot is shown. *Bottom*: Results of quantification of 3 technical repeat westerns from 3 biological repeats (sorts), using Aida software. Data (ratio of relative IRS1 levels:relative βCOP levels) are normalized to young pre-B cells and are means ± SD. Statistical analysis on raw data was with ANOVA combined with Bonferroni’s multiple comparison test. *p = 0.0278; **p = 0.0199

We sorted cells from pooled cohorts of male young and aged mice (12–15 mice per cohort) as shown in Fig. 1a and generated transcriptome, chromatin accessibility (ATAC-seq), histone modification (ChIP-seq), and chromosome conformation (Hi-C) datasets to identify changes associated with the aging phenotype (summary statistics for all datasets and replicates are provided in Additional file 1: Table S1). Transcriptome analysis of ribosomal RNA (rRNA)-depleted total RNA identified 82 significantly upregulated and 54 downregulated genes in aged pro-B cells and 23 upregulated and 33 downregulated genes in aged pre-B cells (Fig. 2a, b; Additional file 1: Tables S2 and S3). Seventeen of these differentially expressed genes (DEGs) were detected in both pro- and pre-B cells (including age-upregulated *Rnf125*, *Dock9*, *Iigp1*, *Igj*, and age-downregulated *Irs1*, *Rftn2*, *Plxna2*, and *Igf2bp3*). Overall, age-specific expression changes were correlated between pro- and pre-B cells, even when significance was reached in only one of the cell types (Fig. 2c). Several of the age-upregulated genes, particularly in pro-B cells, have known roles in late stages of B cell development; these include *Igj*, *Faim3* (Fc receptor for IgM), and genes coding for Major Histocompatibility Complex proteins, such as *Cd74* (Additional file 1: Table S2). This could be explained by aberrant expression of mature B cell-specific genes in the aged pro-B cells. However, we cannot exclude the presence of a small number of contaminating mature B cells in the sorted aged pro-B cell population. The latter may be due to the increased number of recirculating mature B cells, and the less distinct separation between B cell precursors (IgM-) and mature cells (IgM+), in the aged bone marrow (Fig. 1a). Therefore, we excluded mature B cell-specific genes from further analyses and chose to focus primarily on age-downregulated genes, since we are confident that they do not arise from this potential contamination.

Many genes with age-associated changes in expression also showed a strong modulation of their expression during B cell development (Additional file 2: Figure S1a). Genes whose expression decreased upon aging in pro-B cells were frequently downregulated during the pro-B to pre-B cell transition in young cells (Additional file 2: Figure S1a, blue points); examples include *Rftn2*, *Plxna2*, *Cdc42bpa*, *Plxdc2*, and *Pcdh9* (Additional file 2: Figure S1b). This suggests that these genes are either prematurely downregulated in aged pro-B cells or that they fail to be upregulated at an earlier developmental stage in the aged cells. Conversely, genes with reduced expression in aged pre-B cells were often upregulated in the pro-B to pre-B cell transition in young mice (Additional file 2: Figure S1a, red points); examples include *Irs1* (Insulin Receptor Substrate 1) and the PI3K-AKT pathway antagonist *Inpp4b* (reviewed in [17]; Additional file 2: Figure S1b)*.* This pattern suggests a failure to upregulate these genes as the aged B cell progenitors progress to the pre-B cell stage. Notably, several genes downregulated upon aging at the pro-B and/or pre-B cell stage encode components of the IGF signaling pathway, such as *Irs1* and *Igf1r*. Indeed, *Irs1*, a key component of this pathway, was found to be the most significantly downregulated gene in pre-B cells upon aging at the messenger RNA (mRNA) level. We therefore examined IRS1 protein levels and found that these were decreased upon aging in both pro-B and pre-B cells (Fig. 2d), demonstrating that age-specific mRNA changes of *Irs1* are propagated into reduced protein levels.

**Fig. 3:**
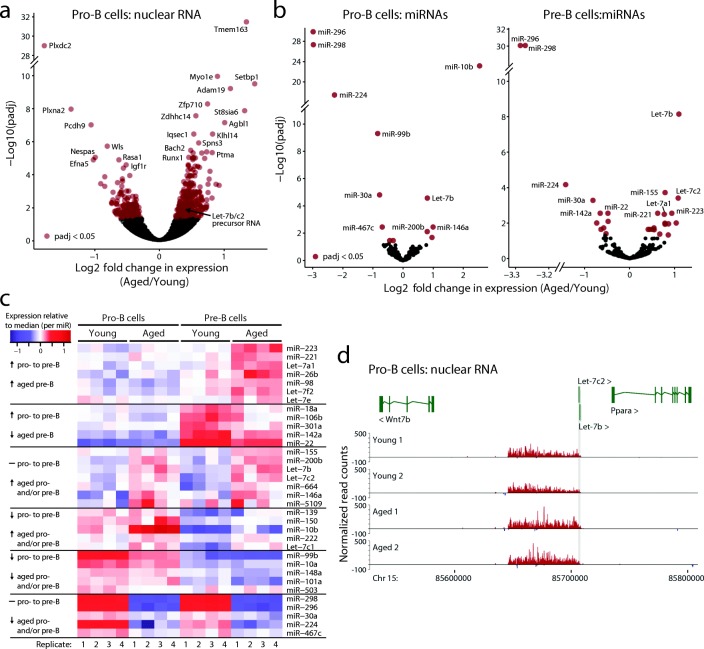
Nascent RNA and miRNA expression changes upon aging in pro- and pre-B cells. **a** Nuclear RNA-seq from pro-B cells identifies significant changes in expression of nascent and non-coding transcripts between B cell precursor cells from young and aged mice. **b** Small RNA-seq from pro-B (*left*) and pre-B cells (*right*) identifies significant changes in expression of miRNAs between B cell precursor cells from young and aged mice. **c** Hierarchical clustering of miRNAs that are differentially expressed upon aging in pro- and/or pre-B cells identifies groups of co-regulated miRNAs. Several of these clusters also display developmental co-regulation from the pro-B to the pre-B cell stage. *Horizontal black lines* indicate the major clusters identified, with trends outlined on the left hand side of the *heatmap*. **d** A novel non-coding RNA encompassing *Let-7b/c2* is differentially expressed between young and aged pro-B cells. Read counts were generated over 100-bp windows and normalized using size factors from DESeq2. Reads originating from the forward strand are represented as positive values (*red bars*), while those originating from the reverse strand are represented as negative numbers (*blue bars*). Genes are indicated at the *top*; *shading* indicates the location of the *Let-7b* and *-c2* genes

Total RNA levels reflect both the rate of transcription and downstream processes such as RNA stability. To explore the effects of aging on gene transcription more directly, we isolated nuclear RNA from young and aged pro-B cells to enrich for nascent transcripts and profiled global changes in intronic transcription as a specific measure of nascent transcription. While ~ 17% (30 out of 175) of DEGs detected in total RNA were also DEGs in the nuclear RNA-sequencing (RNA-seq) analysis, and overall the fold changes were correlated (Additional file 2: Figure S2), this analysis revealed many more genes showing significant age-related differential transcription (Fig. 3a; Additional file 1: Table S4; 147 downregulated and 255 upregulated genes upon aging). Notably, several age-downregulated genes, such as *Plxdc2* [18], *Igf1*, *Igf2bp3*, and *Igf1r*, and upregulated genes, such as *Adam19* [19] and *Tmem163* [20], have been linked to IGF signaling or type 2 diabetes. More broadly, KEGG pathway analysis of DEGs highlighted several metabolic pathways linked to nutrient signaling (Additional file 2: Figure S3).

The nuclear RNA-seq analysis revealed *Nespas* as one of the most significantly downregulated genes in aged pro-B cells (Fig. 3a, Additional file 2: Figure S4a)*. Nespas* is a non-coding transcript implicated in the regulation of imprinting and serves as the non-coding precursor RNA of miRNAs *miR-296* and *miR-298* [21, 22]. To explore the link between miRNA expression and aging in B cell precursors, we performed small RNA-seq. We identified 34 significantly differentially expressed miRNAs in either pro- or pre-B cells (Fig. 3b, c; Additional file 1: Table S5). Of these, 20.6% (7 out of 34) were differentially expressed in both pro- and pre-B cells. This analysis confirmed a profound downregulation of *miR-296* and *miR-298*, consistent with changes in *Nespas* levels detected with nascent RNA-seq. We also observed an upregulation of seven *Let-7* family members and of *miR-223*, upon aging in pro- and/or pre-B cells (Fig. 3b, c). Differentially expressed miRNAs segregated into clusters displaying similar expression changes upon aging, several of which also showed a modulation in their expression between pro-B and pre-B cells (Fig. 3c).

KEGG pathway analysis of validated target genes of the differentially regulated miRNAs (Additional file 1: Tables S13-S16) showed that they mapped to multiple pathways, including those related to cancer, PI3 kinase, and FoxO signaling, suggesting the potential post-transcriptional modulation of these miRNA target genes in aging (Additional file 2: Figure S5).

*Let-7* miRNAs consist of 12 members that share the same seed sequence and are expressed from eight genomic loci (*Let-7a1*, *Let-7a2*, *Let-7b*, *Let-7c1*, *Let-7c2*, *Let-7d*, *Let-7e*, *Let-7f1*, *Let-7f2*, *Let-7g*, *Let-7i*, and *miR-98*) [23]. As *Let-7b* and *-7c2* were the most significantly upregulated miRNAs in aged pre-B cells, we examined the nuclear RNA-seq data and identified an unannotated long, apparently non-coding RNA on chromosome 15 that was significantly increased in the aged pro-B cells and that overlaps with *Let-7b* and *-7c2* (Fig. 3a, d). Thus, this transcript is likely a precursor RNA for these miRNAs. We also noted the presence of a long transcript encompassing *Let-7a1*, *−7d*, and *-7f1*, although its expression was not significantly altered in aged pro-B cells (Additional file 2: Figure S4b).

Taken together, these results demonstrate that aging leads to specific changes in the developing B cell transcriptome affecting coding, long non-coding, and miRNA transcripts.

### Changes in chromatin structure underlie alterations in gene expression in aged B cell precursors

**Fig. 4:**
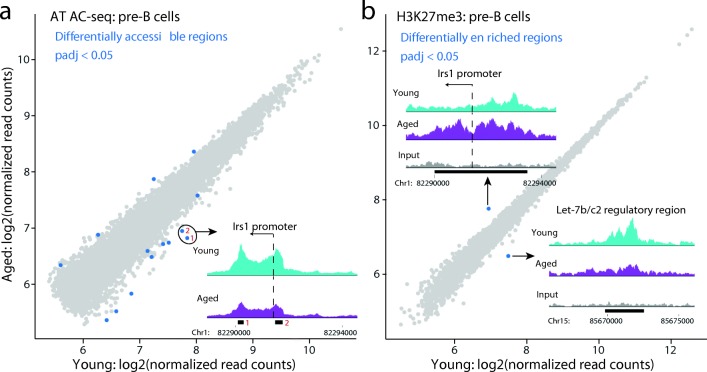
Age-associated changes in chromatin accessibility and H3K27me3 in pre-B cells. **a** DESeq2 analysis of read counts over ATAC-seq peaks in pre-B cells reveals 4 regions with increased chromatin accessibility and 10 regions with reduced accessibility upon aging. Three of the latter overlap with the *Irs1* gene; two of these are illustrated in the insert. **b** DESeq2 analysis of read counts over H3K27me3 peaks in pre-B cells reveals only two regions with a significant alteration in this repressive mark upon aging. The first overlaps with the *Irs1* promoter region, where H3K27me3 is significantly increased. The second overlaps with a potential regulatory region/alternative promoter for the *Let-7b/c2* precursor, where there is a significant decrease in H3K27me3. Both are illustrated in *inserts*. For inserts, median normalized reads for aged and young are shown on the same scale. *Black bars* beneath the plots indicate the peak locations

To explore the mechanisms of age-specific transcriptional modulation, we profiled chromatin accessibility by ATAC-seq [24] in developing B cells from young and aged mice. In aged pro-B cells, accessibility was significantly increased in only five regions and decreased in 12 regions genome-wide (Additional file 2: Figure S6a; Additional file 1: Table S6). Interestingly, two of the regions with decreased accessibility mapped to the promoter of *Plxna2*, whose expression is decreased in aged pro-B cells (as found by total RNA-seq and nuclear RNA-seq). In aged pre-B cells, we identified ten regions with significantly lower accessibility and four regions with higher accessibility (Fig. 4a; Additional file 1: Table S6). Two of the regions with reduced accessibility overlapped with the *Irs1* promoter region and another one mapped close to the *Irs1* transcription termination site (Fig. 4a; Additional file 2: Figure S7a). Therefore, ATAC-seq revealed a limited number of changes in chromatin accessibility upon aging and implicated a role for chromatin remodeling in repressing the *Irs1* locus in aged B cell precursors.

Transcriptional regulation and chromatin accessibility are associated with changes in the binding of regulatory and architectural factors, as well as alterations in histone modifications. Therefore, we generated genome-wide profiles of histone modifications associated with active promoters (H3K4me3, H3K27ac), active enhancers (H3K27ac), and polycomb repressive complex binding (H3K27me3), as well as binding of the architectural protein CTCF in young and aged B cell precursors (summary statistics for all datasets are presented in Additional file 1: Table S1).

For H3K4me3, we found 87 differentially enriched peaks in pro-B cells, with a loss of H3K4me3 detected at 19 of these sites, including the *Irs1* promoter region, and a gain of H3K4me3 at 68 sites. The latter regions included a potential regulatory region which may serve as an alternative promoter for the *Let-7b/c2* precursor RNA (chromosome 15) and the promoter of the *Let-7a1/d/f1* precursor (chromosome 13) (Additional file 2: Figures S6b and S7b; Additional file 1: Table S7). Changes in H3K4me3 overlapped significantly with DEGs (Additional file 2: Figure S6b; as expected, increase of H3K4me3 was only linked to increased expression and vice versa). In pre-B cells, we identified 15 differentially enriched H3K4me3 peaks between young and aged, including a loss of this mark at the *Irs1* promoter region and a gain at the promoter of the age-upregulated gene *Reln*, as well as at the precursors to the *Let-7b/c2* and *Let-7a1/d/f1* miRNAs (Additional file 2: Figures S6b and S7c; Additional file 1: Table S7).

For the polycomb-mediated histone modification H3K27me3, we identified only two peaks with a significant differential enrichment between young and aged pre-B cells (Fig. 4b; Additional file 1: Table S8). The first peak was located at the *Irs1* promoter and showed a significantly higher enrichment in aged pre-B cells, consistent with *Irs1* transcriptional downregulation. The second peak, displaying a decrease in H3K27me3 upon aging, was located in the *Let-7b/c2* regulatory region on chromosome 15 (Fig. 4b; Additional file 2: Figure S8a, b). This is in line with our finding that nascent transcription over this region was significantly upregulated in aged pro-B cells compared to young and consistent with *Let-7b* and *-7c2* being the most upregulated miRNAs in aged pre-B cells. Comparing young to aged pro-B cells, no H3K27me3 peaks passed the multiple testing correction in the differential enrichment analysis (Additional file 1: Table S8). However, the peak overlapping the *Let-7b/c2* precursor displayed a lower enrichment in aged pro-B cells, similar to pre-B cells (Additional file 2: Figure S6c). Consistent with the developmental upregulation of *Irs1* expression observed during B cell differentiation, we observed a decrease in H3K27me3 in young pre-B compared to young pro-B cells at the *Irs1* promoter region (Additional file 2: Figure S9b). This decrease in H3K27me3 did not occur to the same extent in the aged pre-B cells (Additional file 2: Figure S9b). Thus, *Irs1* repression in aged pre-B cells appears to arise due to a failure to relieve polycomb-mediated silencing in the transition from the pro-B to the pre-B cell stage.

Acetylation at lysine 27 of histone H3 is mutually exclusive to methylation of this residue and is linked to active enhancers or promoters. We noted a trend towards increased H3K27ac enrichment at peaks overlapping or within 10-kb of the upregulated genes in the aged pro-B and pre-B cells, while the opposite was true for downregulated genes (Additional file 2: Figure S6d). Notably, H3K27ac displayed reciprocal changes to H3K27me3 at the promoter of *Irs1* and the *Let-7b/c2* precursor (Additional file 2: Figures S6d, S8, and S9). Analysis of CTCF ChIP-seq data did not reveal any significant differential binding of CTCF between young versus aged pre-B cells (Additional file 2: Figure S6e).

**Fig. 5:**
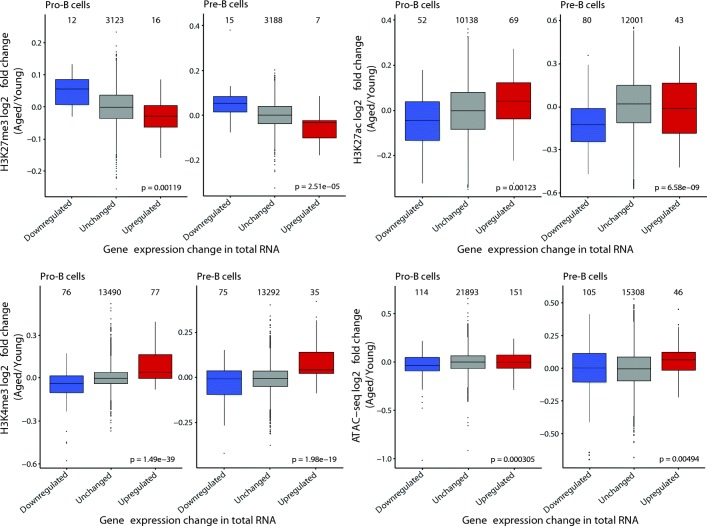
Gene expression changes are linked to chromatin changes upon aging in B cell precursors. Log2 fold change (Aged/Young) in median read counts over peaks for H3K27me3, H3K27ac, H3K4me3, and ATAC-seq that are close to genes identified as up- or downregulated in total RNA-seq data, compared to peaks that are close only to genes whose expression is not significantly altered. Peaks were assigned to a gene if they overlapped with a window extending 1-kb up- and downstream of the gene. ANOVA was used to determine *p* values; these were then FDR adjusted to correct for multiple testing. Numbers above each *boxplot* indicate the number of peaks in each category

The stringent threshold-based approach presented above identified high-confidence loci showing changes in chromatin accessibility and histone modifications upon aging, revealing remarkably few such changes, but highlighting a significant chromatin component to the transcriptional regulation of *Irs1* and the *Let-7* miRNAs. However, this does not exclude the possibility that more subtle changes in the epigenomic landscape might play a broader role in shaping the gene expression profile of aged compared to young B cell precursors. Therefore, we tested whether changes in chromatin, identified using a low stringency threshold, were generally accompanied by altered gene expression (Additional file 2: Figure S10; Additional file 1: Tables S6–S9) and whether genes with altered expression were characterized by remodelled chromatin (Fig. 5). This revealed not only that changes in chromatin upon aging, especially H3K4me3 and H3K27me3 occupancy, likely impact on gene expression (Additional file 2: Figure S10), but further, that if there is a change in gene expression, this is frequently linked to changes in chromatin (Fig. 5). It is noteworthy that H3K4me3 and H3K27ac agree more with differential gene expression than chromatin accessibility (ATAC-seq; Fig. 5; Additional file 2: Figure S10). This is in line with the fact that chromatin accessibility is not necessarily linked to active gene expression but can also be found over other regulatory elements such as insulators, while H3K4me3 and H3K27ac are more strongly linked to actively transcribed genes [25, 26].

In order to examine the relationship between the different types of chromatin alterations that we have analyzed, we identified overlapping peaks between all pairwise combinations of ChIP-seq and ATAC-seq data within a given cell type (pro-B or pre-B) and between pro-B and pre-B cells for a given chromatin feature. We then derived correlation coefficients comparing the age-related log2 fold changes in enrichment for overlapping peaks from each pairwise combination (Additional file 2: Figure S11, left). The highest correlation was observed for log2 fold changes in the same chromatin mark in pro-B versus pre-B cells, consistent with the correlation between age-related gene expression changes at these two developmental stages. We also observed a positive correlation between changes in ATAC-seq and H3K4me3 in aging, but less so between either of these and H3K27ac, in line with the non-redundant nature of these chromatin features [26, 27]. The majority of correlation coefficients increased when considering only pairs of peaks for which at least one ranked highly (top 10%) in significance (Additional file 2: Figure S11, right). Most strikingly, changes in H3K27me3 anti-correlate with changes in ATAC-seq, H3K4me3, and H3K27ac, suggesting coordinated changes in chromatin marks upon aging.

**Fig. 6:**
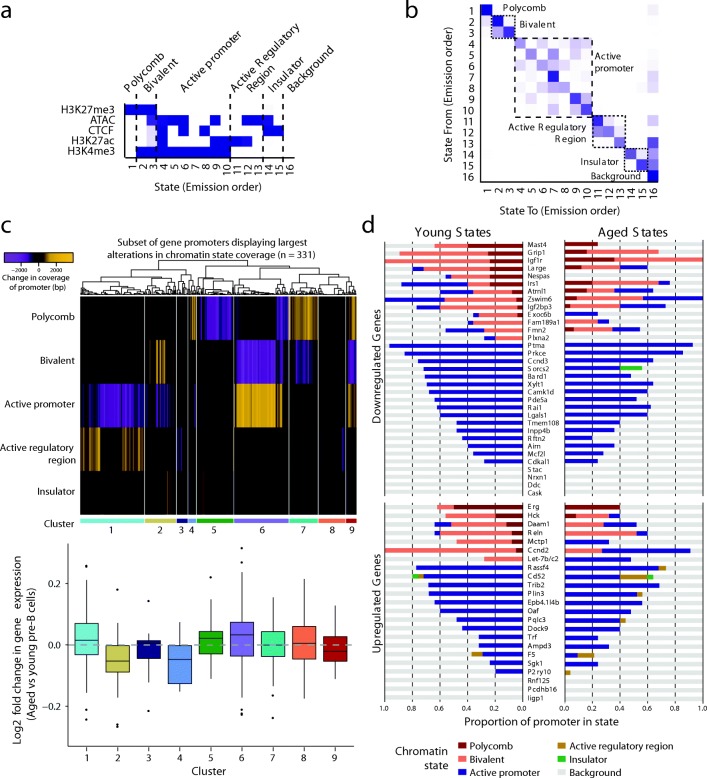
Integrated chromatin state analysis in pre-B cells identifies age-related changes. **a** Emission parameters learnt by the Hidden Markov Model in chromHMM analysis. It shows the likelihood of emitting each of the 5 marks used in this analysis at any given chromatin state. **b** Transition parameters learnt by the HMM in chromHMM analysis: given the current chromatin state (at a specific 200-bp genomic region), how likely is it that the adjacent 200-bp region will be in any of the 16 inferred states. *Dashed blocks* show states that transit to each other more frequently suggesting a more biologically meaningful chromatin state, thus driving our clustering into 6 states: Polycomb, Bivalent, Active Promoter, Active Regulatory Region, Insulator, and Background. **c** *Top*: *Heatmap* showing the change in the occupancy (bp) of each regulatory chromatin state over gene promoter regions (2500-bp up- and downstream of TSS), for a subset of genes showing the largest magnitude changes. Genes are hierarchically clustered based on their correlation and assigned to 9 clusters showing distinct patterns. *Bottom*: Log2 fold change in expression of genes in each cluster, in aged versus young pre-B cells. *Dashed line* indicates unchanged gene expression. To better display the data, some outliers are not displayed. **d** Fraction of promoter regions (defined as for **c**) of DEGs occupied by each of the 6 chromatin states in young and aged pre-B cells. Note that Let-7b/c2 refers to the potential regulatory region/alternative promoter over which we have observed significant alterations in chromatin marks

To further increase the power of the analysis, we next sought to identify age-associated changes in the chromatin landscape jointly across histone marks, chromatin accessibility (as assayed by ATAC-seq) and CTCF occupancy. To do this, we employed chromHMM, an unsupervised machine-learning approach that employs a multivariate Hidden Markov Model and segments the genome into characteristic chromatin states [28]. We chose a 16-state model based on balancing within-class homogeneity and between-class heterogeneity (Fig. 6a). This model was further collapsed into six broader classes (Fig. 6a, b), termed ‘polycomb’ (prevalence of repressive H3K27me3 mark), ‘bivalent’ (presence of active H3K4me3 and repressive H3K27me3 marks), ‘active promoter’ (prevalence of H3K4me3), ‘active regulatory region’ (H3K27ac), ‘insulator’ (CTCF), and ‘background’ (Additional file 3; Additional file 4). We first measured the coverage of each chromatin state over both promoters and regulatory elements (defined as the union between ATAC-seq and ChIP-seq peaks for histone modifications and CTCF in pre-B cells) genome-wide and assigned each to the chromatin state covering the largest proportion, excluding background except if no other state was present. The proportion of regions assigned to each state was very similar in young and aged cells, particularly for promoters (Additional file 2: Figure S12a, b). Over half of all promoters were in the active promoter state, with significant subsets also assigned to the polycomb and bivalent states; indeed the majority of regulatory regions in these three states mapped to a transcription start site (TSS), in contrast to the active regulatory region and insulator states which were rarely localized at TSSs (Additional file 2: Figure S12c). The chromatin states of the vast majority of both promoters and regulatory regions were unchanged in aged compared to young pre-B cells (Additional file 2: Figure S12d).

To extend this analysis and identify more subtle changes that do not necessarily result in an overall switch in the state, we then calculated the difference in the total number of base pairs occupied by each state in aged compared to young pre-B cells. While the majority of promoters displayed minimal changes in state occupancy upon aging (Additional file 2: Figure S12), we selected a subset of genes that displayed the largest magnitude changes over their promoters. In order to integrate these changes and identify groups of genes whose promoters displayed similar transitions in their chromatin state, we used unsupervised clustering. This segregated the promoters into nine clusters displaying different patterns of state changes (Fig. 6c, top; Additional file 1: Table S10). These genes also frequently displayed modulations in gene expression consistent with their altered chromatin state (Fig. 6c, bottom). For example, expression of genes that transitioned from the active promoter state to the bivalent or polycomb state (clusters 2 and 4, respectively) was generally reduced upon aging. Conversely, genes showing the opposite transition (cluster 6) or whose polycomb state occupancy was decreased (cluster 5), tended to increase in expression (Fig. 6c, bottom). We next assessed chromatin state changes at the promoters of all genes showing significant age-specific expression changes in pre-B cells. Promoters of age-upregulated genes showed only subtle changes, with a few notable exceptions, including the newly identified *Let-7b/c2* precursor gene, *Ccnd2* (cyclin D2), and *Mctp1* (multiple C2 and transmembrane domain-containing protein 1). In these cases, the promoter regions switched from a bivalent dominant state in young to a more active state in aged pre-B cells (Fig. 6d). Both *Ccnd2* and *Mctp1* were also assigned to cluster 6 in the genome-wide analysis (Fig. 6c). For genes downregulated in aged pre-B cells, occupancy of the active promoter or active regulatory region state was reduced over 19 of the 32 promoters. Similar to the observation for the majority of upregulated genes, most of these changes were subtle, consistent with the relatively small fold changes in the expression of these genes (Fig. 2b) and the binary nature of chromHMM (at a given genomic location, a peak for a specific chromatin feature is either present or absent). However, we observed substantial changes occurring over the *Irs1* promoter, where the active state transitioned to a bivalent state upon aging (Fig. 6d); it was also identified in the genome-wide analysis, segregating in cluster 2 (Fig. 6c). Chromatin state analysis thus highlights genes that show the most profound alterations in the chromatin at their promoters.

Taken together, these results demonstrate specific age-associated changes in the chromatin at gene promoters in developing B cells, which potentially underlie the observed age-specific changes in gene expression.

### Age-specific changes in genome organization in B cell precursors

**Fig. 7:**
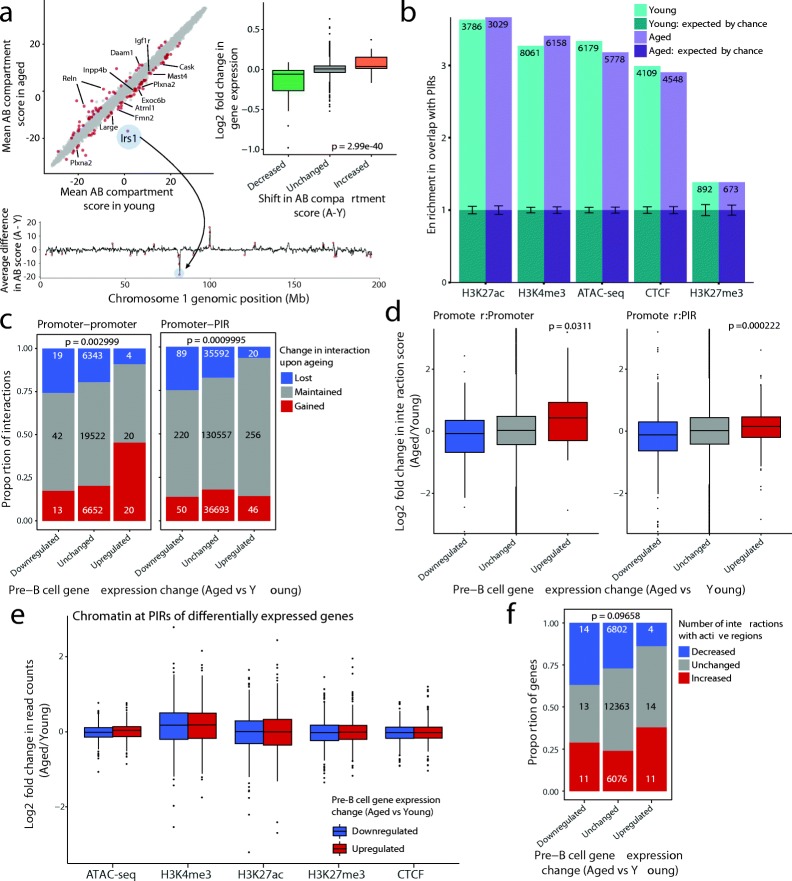
Genome organization changes in pre-B cells upon aging. **a** *Top left*: Mean AB compartment strength (first principal component of Hi-C matrices) in aged vs young mice, at 250-kb resolution. *Red points* represent bins showing significant differences in compartment strength (*p* < 0.05, ANOVA without multiple testing correction but with standardized compartment strength change > 3). The bin encompassing *Irs1* is highlighted; DEGs that lie within bins with a significant shift are indicated. *Bottom*: Average shift in compartment strength between aged and young pre-B cells (Aged - Young; A-Y) on chromosome 1, at 250-kb resolution. Positive peaks represent bins that shift away from the inactive B compartment and towards the active A compartment in aged mice; significantly altering regions are shown in *red*. The bin containing *Irs1* is highlighted: it shifts to a more inactive compartment in aged pre-B cells. *Top right*: Log2 fold change in expression (aged vs young pre-B cells) of genes located within bins showing a significant increase or decrease in AB compartment score. ANOVA was used to determine the *p* value. **b** Observed over expected enrichment in the number of overlaps between promoter interacting regions (PIRs) and ATAC-seq or ChIP-seq peaks for young and aged pre-B cell PCHi-C datasets. *Error bars* indicate the 95% confidence interval in the expected overlaps. *Numbers* indicate the total number of overlaps for each feature. **c** Proportion of all promoter interactions that are gained or lost based on the gene expression changes in pre-B cells upon aging. The total number of interactions in each category is shown on each bar. FDR-adjusted *p* values are based on a Fisher’s exact test for independence of the proportions of gained/lost interactions across gene categories. **d** Log2 fold change in the promoter interaction scores from CHiCAGO for genes whose expression is upregulated, unchanged or downregulated in pre-B cells. FDR-adjusted *p* values are based on ANOVA. **e** Log2 fold change upon aging in median read counts (normalized to library size for each replicate) for ATAC-seq and ChIP-seq datasets over the PIRs of genes whose expression is significantly up- or downregulated in aged pre-B cells. *Boxplots* for upregulated genes comprise log2 fold changes over 322 PIRs from 16 genes, while for downregulated genes *boxplots* comprise 359 PIRs from 20 genes. **f** Proportion of genes that display an overall increase or decrease in the total number of active regions that they contact (including both other promoters and PIRs) in aged compared to young pre-B cells. An increase or decrease might be due to gain/loss of interactions, or a change in the chromatin state of the interacting region. *P* value based on a Fisher’s exact test for independence of the proportion of genes that gain/lose interactions across gene categories

Aging has been linked with changes in genome organization in other systems [29]. In order to address whether such changes occur in developing B cells and whether these correlate with changes in gene regulation, we first performed Hi-C in nuclei isolated from pre-B cells. We used Hi-C data to segment the genomes into A (active) and B (repressed) compartments using principal component analysis (PCA) combined with H3K4me3 data [30]. This analysis showed that the chromosome compartmentalization was near identical between young and aged pre-B cells. However, 100 out of 9928 regions displayed a significant change in their compartment score upon aging (Fig. 7a top left; Additional file 1: Table S11). The compartment score was significantly increased for 40 regions, three of which switched from a repressive B compartment (negative score) to an active A compartment (positive score). Conversely, 60 regions showed a significant decrease in compartment score, with six switching from the A compartment to the B compartment. Notably, one of the A-to-B switch regions encompassed the *Irs1* gene on chromosome 1 (Fig. 7a, bottom; Additional file 2: Figure S13a). Regions with decreased compartment score overlapped with a total of ten genes whose expression was significantly decreased in aged pre-B cells, including *Plxna2* and *Igf1r*, while two genes whose expression increased upon aging (*Reln* and *Daam1*) were located in regions displaying an increase in compartment score (Fig. 7a, top left; Additional file 1: Table S11). When we compared the expression of all genes located within regions with significantly altered compartment scores, we observed a highly significant trend towards an increase in expression of genes with an increased compartment score and vice versa (Fig. 7a, top right; *p* = 2.99e-40). This suggests that relocation of genes between active and repressive nuclear environments may contribute to the modulation of gene expression upon aging.

To obtain a high-resolution view of promoter interactions in pre-B cells and their dynamics upon aging, we performed Promoter Capture Hi-C (PCHi-C; [31, 32]) on cells from young and aged mice and identified statistically significant promoter interactions using CHiCAGO [33]. PCHi-C enriches for elements that interact with a given promoter (promoter interacting regions [PIRs]), including enhancers and silencers. It also enriches for interactions between promoters, e.g. when they are located in the same transcription factory or share an enhancer. To distinguish such interactions, we analyzed promoter:PIR interactions, where we specifically exclude promoter:promoter interactions, separately from promoter:promoter interactions. Consistent with PCHi-C results in other cell types [31, 32], we observed that PIRs were enriched for features associated with gene regulation including H3K4me3, H3K27me3, and H3K27ac (Fig. 7b). This suggests that many of the detected physical interactions connect promoters with regulatory elements. In both young and aged cells, active promoters frequently interacted with active regulatory regions as well as with other active promoters (Additional file 2: Figure S14a, b), while polycomb-associated and bivalent promoters were enriched for interactions with bivalent promoters and bivalent PIRs, as well as with insulator regions.

We asked how the number and strength of promoter interactions relates to age-specific changes in gene expression (Additional file 1: Table S12). Promoters of age-upregulated genes gained interactions with other promoters in aged pre-B cells and rarely lost interactions with either promoters or PIRs, while downregulated genes frequently lost interactions with both upon aging (Fig. 7c). We then considered changes in the CHiCAGO interaction scores, which give a measure of the confidence with which we detect interactions [33], instead of the binary (present/absent) interaction states. This revealed a general trend towards higher scores at upregulated genes, suggesting stronger or more frequent interactions, and lower scores at downregulated genes, suggesting loss of contacts with regulatory elements (Fig. 7d; Additional file 2: Figure S14c).

**Fig. 8:**
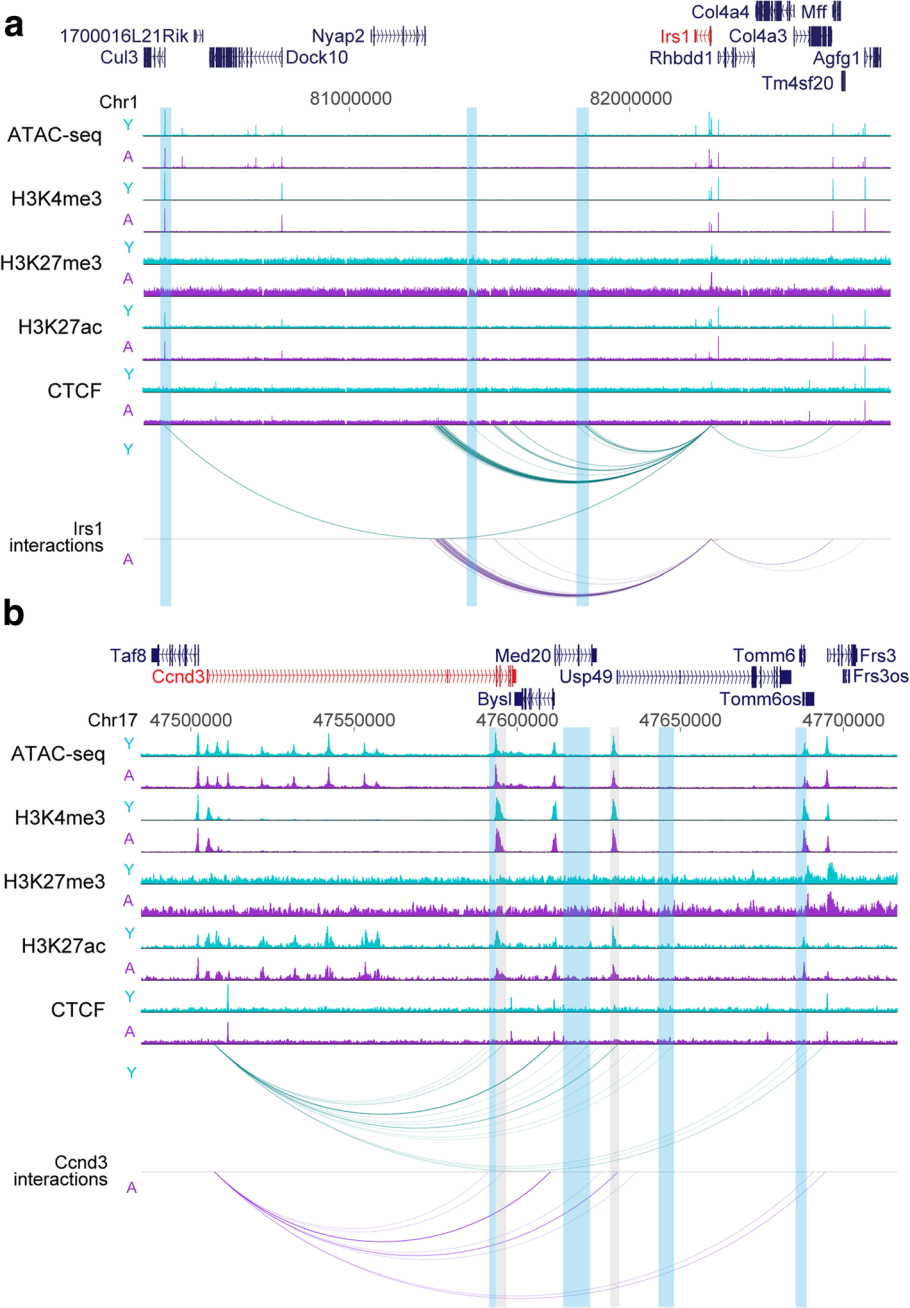
Rewiring of promoter interactions in aged pre-B cells. Genome browser representation of interactions from the *Irs1* (**a**) and *Ccnd3* (**b**) promoters in young and aged pre-B cells. Both are examples of genes whose expression is downregulated upon aging. Shown are all interactions with a CHiCAGO score above the significance threshold of 5 that lie within the genomic region depicted. For ATAC-seq and ChIP-seq tracks, read counts were quantified over 100-bp windows and normalized with size factors from DESeq2 analysis of MACS peaks. For a given locus and chromatin mark, young and aged are displayed on the same scale. *Shading* indicates interactions that are lost upon aging (*blue*) or that display a decrease in H3K27ac enrichment at the PIR (*grey*)

We next explored how age-specific changes in gene expression associated with changes in the chromatin state of the PIRs. In some cases, such as at the *P2ry10*, *Epb4.1l4b*, and *Ccnd3* genes, the PIR of an up- or downregulated gene showed a slight increase or decrease in the levels of active chromatin marks, respectively (Fig. 8b, Additional file 2: Figure S15a, c). However, overall, we did not detect a significant association between gene expression changes and alterations in chromatin at PIRs upon aging (Fig. 7e), in contrast to findings in other systems such as ES cell differentiation [34]. We also observed a number of cases where the promoters of upregulated genes gained connections to regions characterized by an active chromatin state (including both promoters and PIRs), while a number of downregulated genes lost interactions with active regions (Fig. 8, Additional file 2: Figure S15); although, overall, this trend did not reach significance (Fig. 7f). Notably, examples of such ‘rewiring’ of interactions included the age-downregulated *Irs1* gene, which lost several interactions in aged cells, including an interaction with the highly active chromatin surrounding the *Cul3* promoter (Fig. 8a; Additional file 2: Figure S13b).

These results suggest that modulation of the chromatin at PIRs occurs infrequently in aging, even when the expression from the promoter is altered. Rather, our analysis reveals that gene expression changes upon aging are frequently linked to ‘rewiring’ of specific promoter interactions, that might contribute to the broader shifts between active (A) and inactive (B) genomic environments.

## Discussion

### Multilayered chromatin mechanisms underlie age-associated changes in gene expression

Here we present the first study examining how aging affects gene regulation in B cell precursors during their development in the mouse bone marrow. Importantly, our global integrative analysis revealed that, while the chromatin landscape of aged B cell precursors was generally similar to that of young cells, changes in chromatin structure and chromosomal conformation frequently accompanied age-specific alterations in gene expression. In addition to changes in protein-coding gene expression, we identified several long non-coding and miRNAs that are differentially regulated between young and aged B cell precursors, and highlighted chromatin alterations that might play a role therein. Thus, modulation of the underlying chromatin likely contributes to transcriptional alterations upon aging and may play a causative role in some of the gene expression changes that we observed. We found differences in the repressive, polycomb-linked histone modification H3K27me3 particularly noteworthy: this modification has the potential to be heritable from one cell generation to the next by regulating the binding of polycomb repressive complexes [35] and was broadly increased over age-downregulated genes, while upregulated genes displayed decreased enrichment. These opposing trends indicate that it is unlikely that there is a general change in the machinery that sets or removes H3K27me3; indeed, we did not detect altered expression of polycomb group genes. Rather, these differences are more readily explained by gene-specific alterations, such as in signaling pathways to chromatin or the binding of site-specific transcription factors that, in turn, affect H3K27me3. This is illustrated by our observation that modulation of *Irs1* and *Let-7* gene expression was linked to opposing changes in histone H3K27me3 over proximal regulatory elements at those genes. A previous study demonstrated that in skeletal muscle of rapamycin-treated mice (which develop diabetes-like symptoms), *Irs1* was downregulated together with several other insulin signaling-linked genes through PRC1 recruitment and increased H3K27me3; this repression was dependent on the transcriptional regulator YY1 [36]. Consistent with this, the changes in H3K27me3 that we observed in the *Irs1* promoter and the *Let-7b/c2* regulatory region are close to YY1 binding sites. Future work will explore the mechanisms that target H3K27me3 to these genes in pro-B and pre-B cells.

In contrast to the association between gene expression changes and alterations in histone modifications and chromatin accessibility over promoter regions, the chromatin at PIRs was remarkably stable. However, we found that changes in gene expression upon aging were often accompanied by rewiring of the interactions of these promoters with other, potentially regulatory, regions of the genome. Given the absence of chromatin alterations at PIRs, it is conceivable that this rewiring is driven by chromatin modulations at the promoters themselves. Notably, downregulation of genes upon aging was often linked to a loss of promoter interactions, while upregulation was linked to a gain of promoter:promoter interactions. These alterations in specific promoter interactions likely contribute to the shifts that we observed for several DEGs between active and inactive nuclear compartments. Rewiring of promoter interactions has been documented in other systems, especially during differentiation (reviewed in [37]). Here we reveal this layer of genome regulation in an important new context, aging. Together, these analyses shed light on the multiple complementary layers of gene regulation that affect the aging phenotype in B cell precursors.

Interestingly, there was only a partial overlap in the genes differentially expressed upon aging between the total versus nuclear RNA-seq analyses, with more widespread changes identified in the nuclear transcriptome. This might suggest that aging is accompanied by alterations in nascent transcription of numerous genes, whose levels are then buffered by the cells, e.g. through mechanisms involving RNA stability. On the other hand, genes identified only in the total RNA-seq analysis might be transcribed with the same kinetics in young and aged cells, but differently regulated by mechanisms acting post-transcriptionally. The differential expression of several miRNAs that we observed likely contributes to altered protein abundance upon aging. It will be important to explore the numerous facets of post-transcriptional mechanisms and their potential role in aging.

Aging has been linked to increased heterogeneity at the organismal and cellular level [38]. To minimize confounding effects of heterogeneity in this study, we used pools of healthy mice to normalize for inter-individual variation. The fact that we could robustly detect both an increase of H3K27me3 over the age-downregulated *Irs1* promoter and a decrease of this mark over the *Let-7b/c2*-linked regulatory region supports the notion that the gene expression changes we observe are not due to a few ‘outlier’ mice and/or cells, because in the latter case a decrease in H3K27me3 would be difficult to observe, as the signal would be diluted out. Conversely, this normalization approach likely masked gene expression changes within individual aged mice, leading to an underestimate of the numbers of genes affected. While we cannot exclude the possibility that there are additional pathways that may be revealed by analysis of individual mice, we believe we have captured dominant pathways that are altered in aging mouse B cell precursors.

### Precursor B cell aging is associated with gene expression alterations linked to IGF signaling

Our analyses revealed alterations in the IGF signaling pathway at several regulatory levels, suggesting a pivotal contribution to the aging process in B cell precursors. We observed changes in the expression of several key components of the insulin/IGF signaling (IIS) machinery, especially *Irs1* and *Igf1r*, with striking alterations in the chromatin and interaction profile at the *Irs1* promoter/regulatory regions, underscoring that this regulation is in part at the transcriptional level and involves polycomb-mediated repression. We also observed decreased IRS1 protein levels in the aged pro- and pre-B cells compared with the young cells, demonstrating that the lower mRNA level leads to a reduction in the amount of IRS1 protein produced. Notably, downregulation of these key players in the IIS and nutrient signaling pathways has been associated with the aging process, as further discussed below.

Several of the differentially expressed miRNAs that we identified have also been linked to insulin/IGF/metabolic signaling. These include *miR-223*, which modulates *Igf1r* expression [39, 40], and the *Let-7* miRNA family, which regulates *Irs1. Let-7* miRNAs are critical regulators of glucose metabolism and insulin signaling; the upregulation of *Let-7* expression leads to downregulation of expression of several components of the insulin signaling machinery in skeletal muscle and liver, such as *Insr* and *Irs2* [41, 42]. We predict that the increased abundance of *Let-7* in aged B cell precursors would drive a decrease in IIS responsiveness, through targeting *Irs1* and other components at the post-transcriptional level. Notably, we also observed striking alterations in the chromatin at a novel potential precursor RNA for the *Let-7b* and *-7c2* miRNAs, indicative of an interplay between epigenetic and post-transcriptional mechanisms in shaping gene expression.

### Insights into the biology of B cell precursors and aging

The gene expression changes we observed in developing B cells upon aging differ from those that have been detected in hematopoietic stem cells (HSC), where aging in the mouse leads to changes in different classes of genes, such as those involved in TGF-beta signaling [43]. The majority of HSCs in the bone marrow are not proliferative, in contrast to the pro- and pre-B cells. This illustrates that aging affects gene expression in cells within a developmental cascade in different ways, dependent on proliferative status, niche, and other factors.

Nevertheless, genes encoding members of the IIS pathway have been linked to the aging process in other tissues and systems [44–46]. In fact, the nutrient- and growth-linked IIS and mTOR pathways are some of the most conserved aging-controlling pathways in evolution, in worms, flies, and mice [47]. Moreover, members of the *Let-7* miRNA family have previously been linked to the metabolic physiology of aging [48, 49] and show increased expression in human skeletal muscle of aged individuals [50]. Much of this past work linking IIS to aging is based on gene deletion or mutation analysis. Our work highlights changes in gene expression of these components in normal aging and reveals epigenetic mechanisms that are involved.

B cell development is critically dependent on the bone marrow microenvironment, which is altered upon aging. A decrease in secretion of IL7 by aged stromal cells has been linked to impaired generation of B cells in the bone marrow (reviewed in [51]), which is exacerbated by impaired signaling responses to IL7 in aged pro-B cells. Consistent with reduced IL7-dependent survival and proliferation, we observed reduced numbers of pro-B cells in aged mice. Our data additionally implicate IGF-1 signaling in the aged phenotype of the B cell precursors (see above). Previous work has analyzed the involvement of IGF1R signaling in B cell function, showing that *Igf1r* is the predominantly expressed insulin-family receptor in B lymphocytes [52] and that IGF-1, secreted by osteoblasts, is required for normal B cell development in the bone marrow [53]; reviewed in [51]. We observed a more profound reduction in pre-B compared to pro-B cell numbers, concomitant with the onset of IGF-1 signaling in the pro-B to pre-B cell transition [7]. Notably, *Igf1r* deletion in B cell precursors leads to a similar reduction in numbers as we have observed (see Fig. 3 in reference [52]). It is well established that the IIS pathway is subject to multiple levels of feedback regulation. In particular, post-translational regulation of IRS-1 by serine/threonine protein kinases is thought to promote homeostasis with regard to circulating levels of nutrients and insulin (e.g. [54]). Our results uncover a further level of control in this pathway, the chromatin reorganization of key genes, such as *Irs1* and *Let-7*. It will therefore be important to establish the upstream triggers for these changes during aging and the extent to which they are cell autonomous or reflect changes in other components of the bone marrow niche of the aged mouse, such as bone architecture, cell composition or secreted levels of IGF-1 [55, 56]. It will also be important to understand how altered expression of these genes integrates with other central signaling pathways regulating B cell precursor growth, survival, and differentiation. Notably, we did not find alterations in gene expression or epigenetic regulation within these pathways. In particular, we found unaltered expression of the IL7R, consistent with previous studies implicating reduced availability of IL7 and cytoplasmic signaling of the IL7R in the reduction of pro-B cell numbers in aging [13, 57]. Additionally, we did not detect altered expression of SL components of the pre-BCR. Reduced SL cell surface expression observed in aged precursor B cells has been proposed to be an adaptive response to the aging microenvironment which restricts pre-B cell selection [58]. Our data suggest that neither of these pathways is dysregulated at the level of gene expression in aging B cell precursors.

It is noteworthy that *Irs1*^*−/−*^ mice have a longer life- and health-span, with an aged T cell profile that is more comparable to young mice than the control aged mice [44, 45] and that heterozygous IGF-1R mice (*Igfr1*−/+) also show an increased lifespan [46]. Furthermore, there is evidence that reducing IIS leads to greater resilience against cellular stress [46], such as proteotoxicity [59]. Thus, the precursor B cells that remain in aged mice may be those that have adapted best to altered stress conditions in aging. One might speculate that pre-B cells with lower expression of *Irs1* in the aged mice may be more viable and/or the result of a selection process. This could be because greater stimulation of the IRS1-mediated signaling pathway may render cells more susceptible to age-associated stresses, e.g. proteotoxicity [59].

In addition to the genes related to IGF-1 signaling, we identified other DEGs that have the potential to affect the aged phenotype in B cell precursors. Several miRNAs that are differentially expressed between young and aged B cell precursors play important roles in B cell development, including *miR-142a*, *miR-155*, and *miR-221* [60–62]. Therefore, changes in miRNA and gene expression may contribute at multiple levels to reduced B cell precursor numbers and function. An important question that needs to be tackled in the future is whether the pre-B cells in aged mice ultimately develop into B cells with altered immune responses, as has been reported for *Igf1r* KO mice [52].

## Conclusions

In summary, we demonstrate altered gene expression, especially of genes linked to metabolic signaling, in developing B cells upon aging. These changes are connected to alterations in promoter chromatin structure and three-dimensional genome organization. Our study reveals in a well-characterized developmental paradigm that aging affects the regulation of key signaling pathways at multiple levels. A key question for the future is whether these regulatory mechanisms affect other tissues and systems during aging.

## Methods

### Babraham institute C57BL/6 J/Babr aging mouse Colony

Mice were bred and maintained in the Babraham Institute Biological Services Unit under Specific Opportunistic Pathogen Free (SOPF) conditions. C57BL/6 J mice were imported from Charles River Laboratories by embryo transfer and bred as a SOPF colony (/Babr) in plastic film isolators. After weaning, male mice were maintained in individually ventilated cages (2–5 mice per cage). Mice were fed CRM (P) VP diet (Special Diet Services) ad libitum, as well as sunflower seeds, poppy seeds or millet at cage-cleaning as environmental enrichment. Health status was monitored closely and any mouse with clinical signs of ill-health or distress persisting for more than three days was culled. Treatment with antibiotics was not permitted to avoid interference with immune function. Thus, all mice remained ‘sub-threshold’ under UK Home Office severity categorization. As a result of these procedures, mice in the aging colony remained healthy for a median period of 99 weeks. Clinical signs necessitating culling included dermatitis (excessive scratching), fight wounds, and muscle weakness. Post-mortem analysis occasionally revealed neoplasias (especially lymphomas and hemangiosarcomas) typical of the C57BL/6 J strain. Any mice exhibiting gross pathology upon post-mortem examination were excluded from this study.

### Primary cells

Bone marrow was flushed from 12–15 12-week (young) or 19–22 month (aged) healthy male C57BL/6 mice per replicate and depleted of macrophages, granulocytes, erythroid lineage, and T cells using biotinylated antibodies against Cd11b (MAC-1; ebioscience), Ly6G (Gr-1; ebioscience), Ly6C (Abd Serotec), Ter119 (ebioscience), and Cd3e (ebioscience) followed by streptavidin MACs beads (Miltenyi). Thereafter, pro-B (B220^+^CD19^+^CD43^+^CD25^−^IgM^−^) and pre-B (B220^+^CD19^+^CD43^-^CD25^+^IgM^−^) cells were flow sorted on a BD FACSAria in the Babraham Institute Flow Cytometry facility. Antibodies used were CD45R BV421 (B220, RA3-6B2, Biolegend), CD19 PerCP-Cy5.5 (1D3, BD Pharmingen), CD43 FITC (S7, BD Pharmingen), CD25 APC (PC61.5, eBioscience), and IgM PE (eB121-15F9, eBioscience). Purities were > 85% for pro-B and > 90% for pre-B.

The flow cytometry data were quality checked using the automated method in FlowAI [63] and low-quality events were filtered out. Lymphocytes, singlets, and B220 positive cells were gated using FlowJo V10.1 software (FlowJo, LLC) and all compensated parameters were exported for subsequent analysis. Target populations were also manually gated and exported. A tSNE plot was constructed using the Rtsne package with default parameters [64]. To identify populations of interest, manual gates were projected onto the tSNE plot.

### Nuclear and total RNA-seq

For nuclear RNA-seq, nuclei were isolated from 1 to 10 × 10^6^ flow sorted B lymphocytes by incubation in 50 mM Tris-HCl pH 7.5, 140 mM NaCl, 1.5 mM MgCl_2_, 1 mM DTT, 0.4% NP40 for 5 min on ice followed by centrifugation at 500 × *g*. RNA was isolated with a RNeasy mini kit (Qiagen) and treated with Turbo DNAse (Ambion). Total RNA was isolated and depleted of rRNA using a Ribo-Zero Gold rRNA removal kit (Human/Mouse/Rat; Illumina) according to the manufacturer’s instructions. For both nuclear and total RNA, paired-end strand-specific libraries for Illumina sequencing were generated as described [65] except polyA+ RNA selection was omitted, first strand complementary DNA (cDNA) synthesis was performed with random hexamer primers, and double-stranded cDNA was fragmented with a Covaris e220 sonicator. Libraries were sequenced on Illumina HiSeq sequencers according to manufacturer’s instructions.

### Short RNA-seq

Short RNAs were isolated from 1 to 10 × 10^6^ flow sorted B lymphocytes using a mirVANA kit (Ambion/ThermoFisher) and libraries generated directly from this using the NEBNext small RNA library prep set for Illumina with 15 cycles of polymerase chain reaction (PCR). Transfer RNA and rRNA derived fragments were removed from the libraries by ‘double-sided’ AMPure XP size selection as described in the user manual and libraries were sequenced on a HiSeq2500 sequencer (Illumina) according to manufacturer’s instructions.

### ATAC-seq

ATAC-seq was performed on 5 × 10^4^ flow sorted B lymphocytes as described in [24] and libraries were sequenced on a HiSeq2500 sequencer (Illumina) according to manufacturer’s instructions.

### ChIP-seq

Sorted B cells (3–4 million/sort) were fixed in phosphate buffered saline (PBS) containing 1% formaldehyde and 10% fetal bovine serum for 10 min at room temperature. After quenching the fixation with glycine (125 mM final concentration) for 5 min at room temperature, the cells were washed with PBS and the cell pellet was flash frozen in liquid nitrogen and stored at − 80 °C. After defrosting on ice, the cells were then resuspended in sonication buffer (150 mM NaCl, 25 mM Tris-HCl pH 7.4, 5 mM EDTA, 0.1% Triton, 1% SDS) complemented with protease inhibitor cocktail (P8340, Sigma) and, in the case of H3K27ac, with 20 mM Sodium Butyrate and incubated 10–15 min on ice to allow complete lysis. Samples were sonicated using a Bioruptor UCD 200 (Diagenode), for 25 cycles of 30 s ON/30 s OFF, high power. After centrifugation at 14,000 × *g* for 10 min, the supernatant was diluted 5–10 times in ChIP dilution buffer (0.01% SDS, 1.1% Triton, 1.2 mM EDTA, 16.7 mM Tris-HCl pH 8.1, 167 mM NaCl) and 5–25 μg of DNA equivalents (25 μg for CTCF or 15 μg for H3K4me3 ChIP, and 5 μg for H3K27me3) were incubated with either 10 μL of anti-CTCF (Millipore 07–729), 1 μg of anti-H3K27me3 (Active motif 39,155) or 1 μg of anti-H3K4me3 (Active Motif 39,159) antibody overnight on a rotating wheel at 4 °C. A total of 25 μL of protA-G magnetic beads (Millipore 16–663) were added to the samples and incubated on a rotating wheel for 3 h at 4 °C.

For H3K27ac ChIP 0.3 μg of H3K27ac antibody (Abcam ab4729) were pre-incubated with 15 μL protA-G magnetic beads (Millipore 16–663) on a rotating wheel at 4 °C for 4 h; the beads were then washed three times for 5 min on a rotating wheel at 4 °C with RIPA buffer (10 mM Tris-HCl pH 7.5, 140 mM NaCl, 1 mM EDTA, 0.5 mM EGTA, 1% Triton X-100, 0.1% SDS, 0.1% Na-deoxycholate), added to chromatin (5 μg of DNA equivalents), diluted ten times in RIPA buffer, and incubated on a rotating wheel at 4 °C overnight. Antibody-bound beads were washed once with Low Salt buffer (0.1% SDS, 2 mM EDTA, 20 mM Tris-HCl pH 8.0, 150 mM NaCl), twice with High Salt buffer (0.1% SDS, 2 mM EDTA, 20 mM Tris-HCl pH 8.0, 500 mM NaCl), and twice with TE buffer (10 mM Tris-HCl pH 8.0, 1 mM EDTA). For CTCF ChIP the last High Salt buffer was substituted with a LiCl Buffer (0.25 M LiCl, 1% IGEPAL, 1% deoxycholic acid, 1 mM EDTA, 10 mM Tris-HCl pH 8.1). For H3K27ac ChIP the first three washes were performed with RIPA buffer. Each wash was carried out for 5 min on a rotating wheel at 4 °C. ChIP DNA was eluted at 65 °C for 30 min in 200 μL of Elution Buffer (0.1 M NaHCO_3_ and 1% SDS) upon shaking at 1300 rpm. After bringing all inputs to 200 μL with Elution Buffer, all samples were reverse-crosslinked by adding NaCl at 200 mM final concentration and incubating overnight at 65 °C, 300 rpm, followed by addition of Proteinase K (Ambion) to a final concentration of 125 ng/μL and incubation at 65 °C for 2 h. ChIP and input DNA was purified using the QIAquick PCR Purification Kit (Qiagen) and quantified using a Qubit™ 3.0 fluorimeter.

Library preparation was performed from 0.2–0.8 ng of purified DNA using the NEBNext® Ultra™ II DNA Library Prep Kit for Illumina® with the following modifications: Illumina Tru-Seq adaptors were used and library amplification was performed with the KAPA PCR Amplification kit (KAPA, Cat. KK2501) using 15 cycles. Libraries were sequenced on a HiSeq2500 sequencer (Illumina) according to manufacturer’s instructions.

### Hi-C and Promoter Capture Hi-C (PCHi-C)

Hi-C and PCHi-C libraries were generated as described [32] with modifications as detailed below. In total, 3.2 to 3.5 × 10^7^ pre-B cells were fixed in 2% formaldehyde (Agar Scientific) for 10 min, after which the reaction was quenched with ice-cold glycine (Sigma; 0.125 M final concentration). Cells were collected by centrifugation (400 × *g* for 10 min at 4 °C) and washed once with 50 mL PBS pH 7.4 (Gibco). After another centrifugation step (400 × *g* for 10 min at 4 °C), the supernatant was completely removed and the cell pellets were immediately frozen in liquid nitrogen and stored at − 80 °C. After thawing, the cell pellets were incubated in 50 mL ice-cold lysis buffer (10 mM Tris-HCl pH 8, 10 mM NaCl, 0.2% Igepal CA-630, protease inhibitor cocktail [Roche]) for 30 min on ice. After centrifugation to pellet, the cell nuclei (650 × *g* for 5 min at 4 °C) were washed once with 1.25 × NEBuffer 2 (NEB). The nuclei were then resuspended in 1.25 × NEBuffer 2, SDS (10% stock; Promega) was added (0.3% final concentration) and the nuclei were incubated at 37 °C for 1 h with agitation (950 rpm). Triton X-100 (Sigma) was added to a final concentration of 1.7% and the nuclei were incubated at 37 °C for 1 h with agitation (950 rpm). Restriction digest was performed overnight at 37 °C with agitation (950 rpm) with *HindIII* (NEB; 1500 units per 7 million cells). Using biotin-14-dATP (Life Technologies), dCTP, dGTP, and dTTP (Life Technologies; all at a final concentration of 30 μM), the *HindIII* restriction sites were then filled in with Klenow (NEB) for 75 min at 37 °C. The ligation was performed for 4 h at 16 °C (50 units T4 DNA ligase [Life Technologies] per 7 million cells starting material) in a total volume of 5.5 mL ligation buffer (50 mM Tris-HCl pH 7.5, 10 mM MgCl_2_, 1 mM ATP, 10 mM DTT, 100 μg/mL BSA) per 7 million cells starting material. After ligation, crosslinking was reversed by incubation with Proteinase K (Roche; 65 μL of 10 mg/mL per 7 million cells starting material) at 65 °C overnight. An additional Proteinase K incubation (65 μL of 10 mg/mL per 7 million cells starting material) at 65 °C for 2 h was followed by RNase A (Roche; 15 μL of 10 mg/mL per 7 million cells starting material) treatment and two sequential phenol/chloroform (Sigma) extractions. After DNA precipitation (3 M sodium acetate pH 5.2 [1/10 volume] and ethanol [2.5 × volumes]) overnight at − 20 °C, the DNA was spun down (centrifugation 3200 × *g* for 30 min at 4 °C). The pellets were resuspended in 400 μL TLE (10 mM Tris-HCl pH 8.0; 0.1 mM EDTA) and transferred to 1.5 mL Eppendorf tubes. After another phenol/chloroform (Sigma) extraction and DNA precipitation overnight at − 20 °C, the pellets were washed three times with 70% ethanol and the DNA concentration was determined using Quant-iT Pico Green (Life Technologies). For quality control, candidate 3C interactions were assayed (primers available upon request) by PCR and the efficiency of biotin incorporation was assayed by amplifying a 3C ligation product (primers available upon request), followed by digest with *HindIII* or *NheI*.

To remove biotin from non-ligated fragment ends, 40 μg of Hi-C library DNA were incubated with T4 DNA polymerase (NEB) for 4 h at 20 °C, followed by phenol/chloroform purification and DNA precipitation overnight at − 20 °C. After a wash with 70% ethanol, sonication was carried out to generate DNA fragments with a size peak around 400-bp (Covaris E220 settings: duty factor: 10%; peak incident power: 140 W; cycles per burst: 200; time: 55 s). After end repair (T4 DNA polymerase, T4 DNA polynucleotide kinase, Klenow [all NEB] in the presence of dNTPs in ligation buffer [NEB]) for 30 min at room temperature, the DNA was purified (Qiagen PCR purification kit). dATP was added with Klenow exo- (NEB) for 30 min at 37 °C, after which the enzyme was heat-inactivated (20 min at 65 °C). A double size selection using AMPure XP beads (Beckman Coulter) was performed: first, the ratio of AMPure XP beads solution volume to DNA sample volume was adjusted to 0.6:1. After incubation for 15 min at room temperature, the sample was transferred to a magnetic separator (DynaMag-2 magnet; Life Technologies) and the supernatant was transferred to a new Eppendorf tube, while the beads were discarded. The ratio of AMPure XP beads solution volume to DNA sample volume was then adjusted to 0.9:1 final. After incubation for 15 min at room temperature, the sample was transferred to a magnet (DynaMag-2 magnet; Life Technologies). Following two washes with 70% ethanol, the DNA was eluted in 100 μL of TLE (10 mM Tris-HCl pH 8.0; 0.1 mM EDTA). Biotinylated ligation products were isolated using MyOne Streptavidin C1 Dynabeads (Life Technologies) on a DynaMag-2 magnet (Life Technologies) in binding buffer (5 mM Tris-HCl pH 8, 0.5 mM EDTA, 1 M NaCl) for 30 min at room temperature. After two washes in binding buffer and one wash in ligation buffer (NEB), PE adapters (Illumina) were ligated onto Hi-C ligation products bound to streptavidin beads for 2 h at room temperature (T4 DNA ligase NEB, in ligation buffer, slowly rotating). After washing twice with wash buffer (5 mM Tris-HCl pH 8, 0.5 mM EDTA, 1 M NaCl, 0.05% Tween-20) and then once with binding buffer, the DNA-bound beads were resuspended in a final volume of 90 μL NEBuffer 2. Bead-bound Hi-C DNA was amplified with seven PCR amplification cycles (36–40 individual PCR reactions) using PE PCR 1.0 and PE PCR 2.0 primers (Illumina). After PCR amplification, the Hi-C libraries were purified with AMPure XP beads (Beckman Coulter). The concentration of the Hi-C libraries was determined by Bioanalyzer profiles (Agilent Technologies) and qPCR (Kapa Biosystems); the Hi-C libraries were paired-end sequenced (HiSeq 1000, Illumina) at the Babraham Institute Sequencing Facility.

For PCHi-C, 500 ng of Hi-C library DNA was resuspended in 3.6 μL H_2_O and hybridization blockers (Agilent Technologies; hybridization blockers 1 and 2, and custom hybridization blocker) were added to the Hi-C DNA. Hybridization buffers and the custom-made RNA capture bait system (Agilent Technologies; designed as previously described [32, 66]: 39,021 individual biotinylated RNAs targeting the ends of 22,225 promoter-containing mouse *HindIII* restriction fragments) were prepared according to the manufacturer’s instructions (SureSelect Target Enrichment, Agilent Technologies). The Hi-C library DNA was denatured for 5 min at 95 °C and then incubated with hybridization buffer and the RNA capture bait system at 65 °C for 24 h (all incubation steps in a MJ Research PTC-200 PCR machine). After the hybridization incubation, 60 μL of MyOne Streptavidin T1 Dynabeads (Life Technologies) were washed three times with 200 μL binding buffer (SureSelect Target Enrichment, Agilent Technologies), before incubation with the Hi-C DNA/RNA capture bait mixture with 200 μL binding buffer for 30 min at room temperature, slowly rotating. Hi-C DNA bound to capture RNA was isolated using a DynaMag-2 magnet (Life Technologies). Washes (15 min in 500 μL wash buffer I at room temperature, followed by three 10 min incubations in 500 μL wash buffer II at 65 °C) were performed according to the SureSelect Target enrichment protocol (Agilent Technologies). After the final wash, the beads were resuspended in 300 μL NEBuffer 2, isolated on a DynaMag-2 magnet, and then resuspended in a final volume of 30 μL NEBuffer 2. After a post-capture PCR (four amplification cycles using Illumina PE PCR 1.0 and PE PCR 2.0 primers; 13–15 individual PCR reactions), the PCHi-C libraries were purified with AMPure XP beads (Beckman Coulter). The concentration of the PCHi-C libraries was determined by Bioanalyzer profiles (Agilent Technologies) and qPCR (Kapa Biosystems); the PCHi-C libraries were paired-end sequenced (HiSeq 1000, Illumina) at the Babraham Institute Sequencing Facility.

### Computational and statistical methods

All next-generation sequencing reads were first trimmed using Trim Galore [67] and aligned to the GRCm38 mouse genome reference sequence. Statistics for each dataset and replicate are provided in Additional file 1: Table S1.

### RNA-seq analysis

RNA-seq reads were aligned to the GRCm38 reference genome using HISAT2 [68]. Seqmonk version 1.39 [69] was used for quality control, visualization, and quantification. For this, BAM files were imported into Seqmonk as paired-end RNA-seq data and reads with a mapping quality score < 30 were filtered out. Gene and mRNA annotations were filtered to exclude unannotated genes or genes annotated only as a predicted gene, Riken transcripts, pseudogenes, non-functional transcripts, and ribosomal protein subunits. Raw read counts were generated over genes using the RNA quantitation pipeline (total RNA) or counted over mRNA with merged isoforms using the active transcription pipeline, which counts reads only over introns (nuclear RNA), in both cases assuming opposing strand specificity. DESeq2 version 1.12.4 was used for differential expression analysis [70]. For total RNA from both pro- and pre-B cells, we noted a significant batch effect when comparing the first two replicates, which were processed together, with the third replicate which was processed at a later date; this was therefore included in the DESeq2 modeling. Otherwise, default parameters were used, except that ‘independentFiltering’ was set to False and instead only genes with at least 25 reads for at least two replicates in one or both conditions were included. Genes with an adjusted *p* value < 0.05 were labelled as DEGs; for the comparison of pre-B versus pro-B cells in young an additional filter requiring a log2 fold change > 1 was also applied. KEGG pathway analysis was performed using gProfiler [71]. To visualize transcription over specific loci, read counts originating from the forward and reverse strand were quantified over 100-bp windows covering the region of interest using Seqmonk, normalized using DESeq2 size factors from the differential gene expression analysis and visualized in R. Gene annotations for the same regions were imported into R using biomaRt and visualized using the GenomeGraphs package [72].

### MiRNA analysis

MiRNA annotations from Ensembl and miRBase were merged and deduplicated. Reads were mapped to genome build GRCm38 using Bowtie 2 [73] and strand-specific raw read counts were generated using Seqmonk. DESeq2 was then used for differential expression analysis with default parameters and an adjusted *p* value threshold of 0.05. Complete-linkage hierarchical clustering of differentially expressed miRNAs was performed based on the Euclidean distance between the Pearson correlation coefficients between expression patterns for each replicate.

Target genes of differentially expressed miRNAs were identified using mouse mirTarBase v7 database, released in 2017 [74]. Only functionally validated target genes have been considered. The identified target genes for each class have been used for KEGG pathway analysis using DAVID v6.8 online server [75, 76]. *P* values reflect multiple-test corrected (Benjamini–Hochberg) values.

### ATAC-seq analysis

Trimmed reads were aligned to the GRCm38 mouse reference genome using Bowtie 2 [73]. Reads mapping to the mitochondrial genome and alternative contigs were excluded from downstream analysis. Fragment size analysis and quality control was made by a custom in-house code. Fragment coverage BigWig files were constructed using bedtools V2 [77].

We used MACS2 [78, 79] with ‘--nomodel --shif -25 --extsize 50 -q 0.01’ for detection of open chromatin (peaks of read counts). With these parameters, we detected 18,000–51,000 peaks per sample. For each condition (young and aged), a region was accepted as a condition-specific peak if the region was detected as a peak in at least three out of four samples. Two peaks closer than 100-bp to each other were merged. We further pooled young and aged peak sets to get a genome-wide set (union) of open chromatin regions for further differential accessibility analysis. After removing reads with a mapping quality score < 30, peaks were filtered to exclude those with < 64 reads or that overlapped with a blacklisted region [80, 81]. We used featureCounts [82] to count the number of fragments overlapping these regions in each sample. We used DESeq2 [70] for detection of differentially accessible regions, with an adjusted *p* value threshold of 0.05. A lower stringency threshold (unadjusted *p* value < 0.005) was used to identify a larger group of genes over which chromatin accessibility is altered, and the log2 fold gene expression changes were compared between these groups. Peaks were assigned to a gene if they overlapped with a window extending 1-kb up- and downstream of the gene. For comparison of ATAC-seq peaks with genes whose expression was significantly altered upon aging, assignment to genes was performed in the same way. Read counts for each peak were normalized with DESeq2 and the log2 fold change in the median counts (aged/young) was compared for peaks assigned to upregulated genes, downregulated genes or exclusively to genes whose expression was not significantly changed. For pro-B and pre-B cell ATAC-seq data, up- and downregulated genes were assigned from the total RNA-seq analyses from pro-B and pre-B cells, respectively. All upregulated genes predicted to originate from mature B cells were excluded.

### ChIP-seq analysis

ChIP-seq data were mapped to the GRCm38 mouse reference genome using Bowtie 2. For all except H3K27me3, we called peaks using MACS2 with default parameters for narrow peaks. For H3K27me3, we instead used MACS V1.4 to call broad peaks. Similar to ATAC-seq analysis, peaks for aged and young were merged; those with low numbers of high-quality reads (< 32 for CTCF; otherwise < 64) or overlapping blacklisted regions were filtered out and differential enrichment analysis was performed using featureCounts and DESeq2, with adjusted *p* value threshold < 0.05. To identify a larger set of genes for comparison of their log2 fold change in expression, lower stringency thresholds were used (unadjusted *p* value < 0.005 for H3K4me3 and < 0.1 for H3K27me3 and H3K27ac), with peaks assigned to genes as described for ATAC-seq. Comparison with genes whose expression was significantly up- or downregulated was performed as described for ATAC-seq.

### Fold change correlation analyses for chromatin datasets

Overlapping peaks between all pairwise combinations of ChIP-seq and ATAC-seq data within a given cell type (pro-B or pre-B), and between pro-B and pre-B cells for a given chromatin feature, were first defined. These were filtered such that if a single peak in one dataset overlapped with multiple peaks in the second, one of the peaks from the second dataset was selected at random. Pearson correlation coefficients comparing the age-related log2 fold changes in enrichment for overlapping peaks from each pairwise combination were then calculated and displayed as heatmaps. To reduce the influence of stochastic variation in peaks that are unchanged upon aging, peaks from each dataset were then ranked based on their uncorrected *p* values from the DESeq2 differential enrichment analysis (see above), with the smallest (most significant) *p* value ranked highest. Each pairwise set of overlapping peaks was then filtered to retain only pairs of peaks for which at least one of the peaks was ranked in the top 10%, i.e. retaining those peaks with the greatest evidence for an age-related change in enrichment. Pearson correlation coefficients were then derived as above for these filtered sets of overlapping peaks.

### Chromatin state analysis and differential occupancy of states at promoter regions

For chromatin state analysis, we used chromHMM [28]. We took a similar approach to that described in a previous study [26], allowing us to provide both Young and Aged data (analogous to cell types in that study) in one CellMarks file. With this setting, only one Emission and one Transition parameter matrix is inferred. Based on these learnt parameters, the genomic localization of these states is inferred separately for Young and Aged. For this, first, the genome was binned into 200-bp bins and binarized based on the pre-B cell ATAC-seq and ChIP-seq peaks using the ‘binarizeBed’ function. We then ran the ‘learnModel’ function, varying the number of chromatin states in the range of 3–24. We chose 16 states as the optimum; these were then manually collapsed into six distinct and more biologically relevant states, as illustrated in Fig. 6a, b.

Promoter occupancy for each state (bp) was calculated over a 5-kb window centred on the TSS of each gene; the overall state of a given promoter was assigned to the state with the largest coverage, with the exception of the background state to which a promoter was assigned only if no other state was present. A genome-wide set of regulatory elements was defined by merging the MACS peaks called from ATAC-seq and ChIP-seq for H3K4me3, H3K27me3, H3K27ac, and CTCF in pre-B cells; the overall state of each was assigned in the same way as for promoters. States were assigned separately based on young and aged chromatin segmentation, allowing the proportions of promoters and regulatory elements in each state to be compared and to identify regions that changed state upon aging. To assess the overlap of regulatory elements in each state with transcription start sites, all mRNA isoforms (based on Ensembl annotations) were considered; any regulatory element within 1-kb of a transcription start site was considered overlapping. To extend the analysis of promoters, the absolute coverage of all states was considered for each promoter: genes displaying the greatest magnitudes of change in state coverage upon aging (in the top 0.5% for increased or decreased bp coverage for at least one regulatory state, excluding background) were selected and changes in coverage were normalized by subtracting the mean change in bp coverage for a given state. Complete-linkage hierarchical clustering was performed based on the Euclidean distance between the Pearson correlation coefficients between state changes. Genes were segregated into nine clusters to maximize the distinction between state change patterns and the log2 fold change in pre-B cell total gene expression upon aging compared for each cluster. The fraction of the promoter occupied by each state was also calculated for selected age-up- and downregulated genes.

### Hi-C analysis

We mapped and filtered the Hi-C reads using HiCUP [83] and analyzed the filtered Hi-C data using HOMER [84]. Using the binned Hi-C data, we computed the coverage- and distance-related background in the Hi-C data at 250-kb resolution, based on an iterative correction algorithm [85]. The compartment signal was computed as the first principle component of the distance-and-coverage corrected interaction profile correlation matrix at 250-kb resolution, as has previously been described and shown to identify active and inactive compartments [30]. Since PCA does not necessarily assign a positive value to the active compartment, H3K4me3 enrichment in young pre-B cells was compared for positive and negative compartments; positive values were then assigned to the group with high enrichment and vice versa. Genomic 250-kb bins that significantly changed compartment association were identified by ANOVA with a threshold of 0.05 and further filtered to include only bins showing a standardized mean change in compartment score > 3. Genes overlapping each bin were identified and the log2 fold change in their expression in aged versus young pre-B cells (total RNA-seq) was compared.

### PCHi-C analysis

Reads were mapped and filtered using HiCUP [83]. BAM files for duplicate sequencing lanes were merged and deduplicated, and interactions were called using CHiCAGO [33], using a threshold of 5 for significant interactions. An interaction was designated as lost or gained upon aging if it was significant at only one age and the difference in score was at least 2. Interactions for which both ends were baited were designated promoter:promoter interactions, while all others were designated promoter:PIR. Enrichment for overlap of PIRs with ChIP-seq and ATAC-seq features was performed within the CHiCAGO pipeline, using MACS peaks called from our data as described above; overlaps expected by chance were simulated within the CHiCAGO pipeline using 100 random subsets of *HindIII* fragments with similar interaction distances. Each promoter and PIR was assigned to one of the six chromatin states for the chromHMM analysis as follows: active promoter = fragment overlaps with active promoter state and does not overlap with polycomb or bivalent states; polycomb = fragment overlaps with polycomb state and does not overlap with active promoter or bivalent state; bivalent = fragment overlaps with bivalent state or with both active promoter and polycomb states; active regulatory = fragment overlaps with active regulatory state and does not overlap with active promoter, bivalent or polycomb states; insulator = fragment overlaps with insulator state and does not overlap with any other state except background; background = fragment overlaps exclusively with background state. All active fragments were defined as those assigned to the active promoter or active regulatory state, or with overlapping ChIP-seq peaks of H3K4me3 or H3K27ac, excluding any fragments assigned to the polycomb or bivalent state or that overlapped with an H3K27me3 peak. To compare enrichment of ATAC-seq and ChIP-seq over PIRs, total read counts over the PIRs were normalized to the total library size and the log2 fold change in median read count compared for PIRs of up- and downregulated genes, excluding upregulated genes predicted to originate from mature cells. Genome browser interaction plots were generated using the WashU browser [86]. Virtual 4C was performed using Seqmonk to import all ditag reads for which the other end mapped to the baited *HindIII* fragment encompassing the *Irs1* gene promoter. Read counts were quantified over merged *HindIII* fragments, such that each quantitation window comprised five adjacent *HindIII* fragments.

### Western blot

Pre- and pro-B cell pellets were sonicated in SDS sample buffer and proteins resolved by SDS-PAGE. Proteins were transferred onto PVDF membranes and immunoblotted with the indicated primary antibodies at 4 °C overnight, followed by 1 h at room temperature. Membranes were washed in TBST (40 mM Tris-HCl, pH 8.0 at room temperature; 0.14 M NaCl; 0.1% Tween-20) and incubated with HRP-conjugated secondary antibodies. IRS1 antibody #2382 was from Cell Signaling Technology (CST)/New England Biolabs (NEB); anti-beta-COP antibody was a kind gift from Dr. Nick Ktistakis, Babraham Institute, UK; goat Anti-Rabbit and anti-Mouse IgG (H + L)-HRP conjugate was from BioRad. Membranes were washed and signal detected by enhanced chemiluminescence. Relative protein expression was quantified using Aida 2D Densitometry software (v3.27).

## Additional files

Additional file 1: Supplementary Tables S1–S16. XLSX file describing datasets generated for this study. (XLSX 369 kb)

Additional file 2: Supplementary Figures S1–S15. PDF of all supplementary figures S1–S15. (PDF 4237 kb)

Additional file 3: Chromatin segmentation output for young pre-B cells. TXT file listing chromatin segmentation output for young pre-B cells. (TXT 4627 kb)

Additional file 4: Chromatin segmentation output for aged pre-B cells. TXT file listing chromatin segmentation output for aged pre-B cells. (TXT 4190 kb)

Additional file 5: Reviewer reports and authors’ response to reviewers. (DOCX 119 kb)

## Abbreviations

DEG: Differentially expressed gene
IGF: Insulin-like growth factor
IIS pathway: Insulin/insulin-like growth factor signaling pathway
IL7R: Interleukin 7 receptor
miRNA: MicroRNA
PCHi-C: Promoter Capture Hi-C
PIR: Promoter interacting region
pre-BCR: Pre-B cell receptor
SD: Standard deviation
SL: Surrogate light chain
tSNE: t-Distributed Stochastic Neighbor Embedding

## References

[1] Nikolich-Žugich J. The twilight of immunity: emerging concepts in aging of the immune system. Nat Immunol. 2018;19:10–9. 10.1038/s41590-017-0006-x 29242543

[2] Corcoran AE, Smart FM, Cowling RJ, Crompton T, Owen MJ, Venkitaraman AR (1996). The interleukin-7 receptor alpha chain transmits distinct signals for proliferation and differentiation during B lymphopoiesis. EMBO J..

[3] Gibson LF, Piktel D, Landreth KS (1993). Insulin-like growth factor-1 potentiates expansion of interleukin-7-dependent pro-B cells. Blood.

[4] Erlandsson L, Licence S, Gaspal F, Lane P, Corcoran AE, Mårtensson I-L (2005). Both the pre-BCR and the IL-7Ralpha are essential for expansion at the pre-BII cell stage in vivo. Eur J Immunol..

[5] Clark MR, Mandal M, Ochiai K, Singh H (2014). Orchestrating B cell lymphopoiesis through interplay of IL-7 receptor and pre-B cell receptor signalling. Nat Rev Immunol..

[6] Bednarski JJ, Pandey R, Schulte E, White LS, Chen B-R, Sandoval GJ (2016). RAG-mediated DNA double-strand breaks activate a cell type-specific checkpoint to inhibit pre-B cell receptor signals. J Exp Med.

[7] Landreth KS, Narayanan R, Dorshkind K (1992). Insulin-like growth factor-I regulates pro-B cell differentiation. Blood.

[8] Cancro MP, Hao Y, Scholz JL, Riley RL, Frasca D, Dunn-Walters DK (2009). B cells and aging: molecules and mechanisms. Trends Immunol..

[9] Stephan RP, Sanders VM, Witte PL (1996). Stage-specific alterations in murine B lymphopoiesis with age. Int Immunol.

[10] Labrie JE, Sah AP, Allman DM, Cancro MP, Gerstein RM (2004). Bone marrow microenvironmental changes underlie reduced RAG-mediated recombination and B cell generation in aged mice. J Exp Med.

[11] Kline GH, Hayden TA, Klinman NR (1999). B cell maintenance in aged mice reflects both increased B cell longevity and decreased B cell generation. J Immunol.

[12] Johnson KM, Owen K, Witte PL (2002). Aging and developmental transitions in the B cell lineage. Int Immunol.

[13] Stephan RP, Lill-Elghanian DA, Witte PL (1997). Development of B cells in aged mice: decline in the ability of pro-B cells to respond to IL-7 but not to other growth factors. J Immunol.

[14] Moskowitz DM, Zhang DW, Hu B, Le Saux S, Yanes RE, Ye Z (2017). Epigenomics of human CD8 T cell differentiation and aging. Sci Immunol.

[15] Ucar D, Márquez EJ, Chung C-H, Marches R, Rossi RJ, Uyar A (2017). The chromatin accessibility signature of human immune aging stems from CD8 +T cells. J Exp Med.

[16] Miller JP, Allman D (2003). The decline in B lymphopoiesis in aged mice reflects loss of very early B-lineage precursors. J Immunol.

[17] Agoulnik IU, Hodgson MC, Bowden WA, Ittmann MM (2011). INPP4B: the new kid on the PI3K block. Oncotarget..

[18] Jin T, Li J, Wei J, Xu P, Yan M, Li J (2016). Impact of diabetes-related gene polymorphisms on the clinical characteristics of type 2 diabetes Chinese Han population. Oncotarget.

[19] Matthews VB, Weerasekera L, Rudnicka C, Sang Q-X, Curran JE, Johnson MP (2017). ADAM19: a novel target for metabolic syndrome in humans and mice. Mediat Inflamm..

[20] Ghosh S, Sengupta S, Madhu SV, McCarthy MI, Bharadwaj D, Marwaha RK (2013). Genome-wide association study for type 2 diabetes in Indians identifies a new susceptibility locus at 2q21. Diabetes..

[21] Robson JE, Robson JE, Eaton SA, Eaton SA, Underhill P, Underhill P (2012). MicroRNAs 296 and 298 are imprinted and part of the GNAS/Gnas cluster and miR-296 targets IKBKE and Tmed9. RNA.

[22] Williamson CM, Ball ST, Dawson C, Mehta S, Beechey CV, Fray M (2011). Uncoupling antisense-mediated silencing and DNA methylation in the imprinted Gnas cluster. Lee JT, editor. PLoS Genet..

[23] Su J-L, Chen P-S, Johansson G, Kuo M-L (2012). Function and regulation of let-7 family microRNAs. Microrna..

[24] Buenrostro JD, Giresi PG, Zaba LC, Chang HY, Greenleaf WJ (2013). Transposition of native chromatin for fast and sensitive epigenomic profiling of open chromatin, DNA-binding proteins and nucleosome position. Nat Meth..

[25] Thurman RE, Rynes E, Humbert R, Vierstra J, Maurano MT, Haugen E (2012). The accessible chromatin landscape of the human genome. Nature.

[26] Kundaje A, Meuleman W, Ernst J, Bilenky M, Yen A, Roadmap Epigenomics Consortium (2015). Integrative analysis of 111 reference human epigenomes. Nature..

[27] Hoffman MM, Ernst J, Wilder SP, Kundaje A, Harris RS, Libbrecht M (2013). Integrative annotation of chromatin elements from ENCODE data. Nucleic Acids Res..

[28] Ernst J, Kellis M (2012). ChromHMM: automating chromatin-state discovery and characterization. Nat Meth..

[29] Chandra T, Kirschner K (2016). Chromosome organisation during ageing and senescence. Curr Opin Cell Biol..

[30] Lieberman-Aiden E, van Berkum NL, Williams L, Imakaev M, Ragoczy T, Ragoczy T (2009). Comprehensive mapping of long-range interactions reveals folding principles of the human genome. Science..

[31] Mifsud B, Tavares-Cadete F, Young AN, Sugar R, Schoenfelder S, Ferreira L (2015). Mapping long-range promoter contacts in human cells with high-resolution capture Hi-C. Nat Genet..

[32] Schoenfelder S, Furlan-Magaril M, Mifsud B, Tavares-Cadete F, Sugar R, Javierre B-M (2015). The pluripotent regulatory circuitry connecting promoters to their long-range interacting elements. Genome Res..

[33] Cairns J, Freire-Pritchett P, Wingett SW, Várnai C, Dimond A, Plagnol V (2016). CHiCAGO: robust detection of DNA looping interactions in Capture Hi-C data. Genome Biol..

[34] Freire-Pritchett P, Schoenfelder S, Várnai C, Wingett SW, Cairns J, Collier AJ (2017). Global reorganisation of cis-regulatory units upon lineage commitment of human embryonic stem cells. eLife..

[35] Schuettengruber B, Bourbon H-M, Di Croce L, Cavalli G (2017). Genome regulation by Polycomb and Trithorax: 70 years and counting. Cell..

[36] Blättler SM, Cunningham JT, Verdeguer F, Chim H, Haas W, Liu H (2012). Yin Yang 1 deficiency in skeletal muscle protects against rapamycin-induced diabetic-like symptoms through activation of insulin/IGF signaling. Cell Metab..

[37] Andrey G, Mundlos S (2017). The three-dimensional genome: regulating gene expression during pluripotency and development. Development..

[38] Martinez-Jimenez CP, Eling N, Chen H-C, Vallejos CA, Kolodziejczyk AA, Connor F (2017). Aging increases cell-to-cell transcriptional variability upon immune stimulation. Science..

[39] Zhao F-Y, Han J, Chen X-W, Wang J, Wang X-D, Sun J-G (2016). miR-223 enhances the sensitivity of non-small cell lung cancer cells to erlotinib by targeting the insulin-like growth factor-1 receptor. Int J Mol Med..

[40] Chen Z, Han J, Zhao F, Zhang J, Zhu H, Ma H (2016). miR-223 reverses the resistance of EGFR-TKIs through IGF1R/PI3K/Akt signaling pathway. Int J Oncol.

[41] Zhu H, Daley GQ, Urbach A, Gregory RI, Triboulet R, Shyh-Chang N (2011). The Lin28/let-7 axis regulates glucose metabolism. Cell..

[42] Frost RJA, Olson EN (2011). Control of glucose homeostasis and insulin sensitivity by the Let-7 family of microRNAs. Proc Natl Acad Sci U S A.

[43] Sun D, Chen R, Darlington GJ, Faull KF, Luo M, Jeong M (2014). Epigenomic profiling of young and aged HSCs reveals concerted changes during aging that reinforce self-renewal. Cell Stem Cell.

[44] Selman C, Partridge L, Withers DJ (2011). Replication of extended lifespan phenotype in mice with deletion of insulin receptor substrate 1. PLoS ONE.

[45] Partridge L, Robinson ICA, Speakman JR, Al-Qassab H, Thornton JM, Withers DJ (2008). Evidence for lifespan extension and delayed age-related biomarkers in insulin receptor substrate 1 null mice. FASEB J.

[46] Holzenberger M, Dupont J, Ducos B, Leneuve P, Géloën A, Even PC (2003). IGF-1 receptor regulates lifespan and resistance to oxidative stress in mice. Nature..

[47] López-Otín C, Blasco MA, Partridge L, Serrano M, Kroemer G (2013). The hallmarks of aging. Cell..

[48] Micó V, Berninches L, Tapia J, Daimiel L (2017). NutrimiRAging: Micromanaging Nutrient Sensing Pathways through Nutrition to Promote Healthy Aging. IJMS.

[49] Jun-Hao ET, Gupta RR, Shyh-Chang N (2016). Lin28 and let-7 in the metabolic physiology of aging. Trends Endocrinol Metab..

[50] Drummond MJ, McCarthy JJ, Sinha M, Spratt HM, Volpi E, Esser KA (2011). Aging and microRNA expression in human skeletal muscle: a microarray and bioinformatics analysis. Physiological Genomics..

[51] Min H, Montecino-Rodriguez E, Dorshkind K (2005). Effects of aging on early B- and T-cell development. Immunol Rev..

[52] Baudler S, Baumgartl J, Hampel B, Buch T, Waisman A, Snapper CM (2005). Insulin-like growth factor-1 controls type 2 T cell-independent B cell response. J Immunol.

[53] Yu VWC, Lymperi S, Oki T, Jones A, Swiatek P, Vasic R (2016). Distinctive mesenchymal-parenchymal cell pairings govern B cell differentiation in the bone marrow. Stem Cell Rep..

[54] Boucher J, Kleinridders A, Kahn CR (2014). Insulin receptor signaling in normal and insulin-resistant states. Cold Spring Harb Perspect Biol..

[55] Farr JN, Xu M, Weivoda MM, Monroe DG, Fraser DG, Onken JL (2017). Targeting cellular senescence prevents age-related bone loss in mice. Nat Med..

[56] Kim H-N, Chang J, Shao L, Han L, Iyer S, Manolagas SC (2017). DNA damage and senescence in osteoprogenitors expressing Osx1 may cause their decrease with age. Aging Cell..

[57] Sherwood EM, Xu W, Riley RL (2003). B cell precursors in senescent mice exhibit decreased recruitment into proliferative compartments and altered expression of Bcl-2 family members. Mech Ageing Dev..

[58] Ratliff M, Alter S, McAvoy K, Frasca D, Wright JA, Zinkel SS (2015). In aged mice, low surrogate light chain promotes pro-B-cell apoptotic resistance, compromises the PreBCR checkpoint, and favors generation of autoreactive, phosphorylcholine-specific B cells. Aging Cell..

[59] Cohen E, Paulsson JF, Blinder P, Burstyn-Cohen T, Du D, Estepa G (2009). Reduced IGF-1 signaling delays age-associated proteotoxicity in mice. Cell..

[60] Kramer NJ, Wang W-L, Reyes EY, Kumar B, Chen C-C, Ramakrishna C (2015). Altered lymphopoiesis and immunodeficiency in miR-142 null mice. Blood..

[61] Knoll M, Simmons S, Bouquet C, Grün JR, Melchers F (2013). miR-221 redirects precursor B cells to the BM and regulates their residence. Eur J Immunol..

[62] Costinean S, Zanesi N, Pekarsky Y, Tili E, Volinia S, Heerema N (2006). Pre-B cell proliferation and lymphoblastic leukemia/high-grade lymphoma in E(mu)-miR155 transgenic mice. Proc Natl Acad Sci U S A..

[63] Monaco G, Chen H, Poidinger M, Chen J, Pedro de Magalhaes J, Larbi AA (2016). flowAI: automatic and interactive anomaly discerning tools for flow cytometry data. Bioinformatics..

[64] Krijthe J. Rtsne: T-distributed stochastic neighbor embedding using a Barnes-hut implementation. 2015. https://github.com/jkrijthe/Rtsne/. Accessed 27 June 2017.

[65] Parkhomchuk D, Borodina T, Amstislavskiy V, Banaru M, Hallen L, Krobitsch S (2009). Transcriptome analysis by strand-specific sequencing of complementary DNA. Nucleic Acids Res..

[66] Schoenfelder S, Sugar R, Dimond A, Javierre B-M, Armstrong H, Mifsud B (2015). Polycomb repressive complex PRC1 spatially constrains the mouse embryonic stem cell genome. Nat Genet..

[67] Babraham Bioinformatics Trim Galore. https://www.bioinformatics.babraham.ac.uk/projects/trim_galore/. Accessed 6 Oct 2017.

[68] Kim D, Langmead B, Salzberg SL (2015). HISAT: a fast spliced aligner with low memory requirements. Nat Meth..

[69] Babraham Bioinformatics Seqmonk Project. http://www.bioinformatics.babraham.ac.uk/projects/seqmonk/. Accessed 10 Nov 2017.

[70] Love MI, Huber W, Anders S (2014). Moderated estimation of fold change and dispersion for RNA-seq data with DESeq2. Genome Biol..

[71] Reimand J, Arak T, Adler P, Kolberg L, Reisberg S, Peterson H (2016). g:Profiler-a web server for functional interpretation of gene lists (2016 update). Nucleic Acids Res..

[72] Durinck S, Bullard J, Spellman PT, Dudoit S (2009). GenomeGraphs: integrated genomic data visualization with R. BMC Bioinformatics..

[73] Langmead B, Salzberg SL (2012). Fast gapped-read alignment with Bowtie 2. Nat Meth..

[74] Chou C-H, Shrestha S, Yang C-D, Chang N-W, Lin Y-L, Liao K-W (2018). miRTarBase update 2018: a resource for experimentally validated microRNA-target interactions. Nucleic Acids Res.

[75] Huang DW, Sherman BT, Lempicki RA (2009). Bioinformatics enrichment tools: paths toward the comprehensive functional analysis of large gene lists. Nucleic Acids Res..

[76] Huang DW, Sherman BT, Lempicki RA (2009). Systematic and integrative analysis of large gene lists using DAVID bioinformatics resources. Nat Protoc..

[77] Quinlan AR, Hall IM (2010). BEDTools: a flexible suite of utilities for comparing genomic features. Bioinformatics..

[78] Zhang Y, Liu T, Meyer CA, Eeckhoute J, Johnson DS, Bernstein BE (2008). Model-based analysis of ChIP-Seq (MACS). Genome Biol..

[79] Koohy H, Down TA, Spivakov M, Hubbard T (2014). A comparison of peak callers used for DNase-Seq data. PLoS ONE..

[80] ENCODE Project Consortium (2012). An integrated encyclopedia of DNA elements in the human genome. Nature..

[81] Boyle A, Kundaje A. mod/mouse/humanENCODE: Blacklisted genomic regions for functional genomics analysis. https://sites.google.com/site/anshulkundaje/projects/blacklists/. Accessed 10 Aug 2017.

[82] Liao Y, Smyth GK, Shi W (2014). featureCounts: an efficient general purpose program for assigning sequence reads to genomic features. Bioinformatics..

[83] Wingett S, Ewels P, Furlan-Magaril M, Nagano T, Schoenfelder S, Fraser P (2015). HiCUP: pipeline for mapping and processing Hi-C data. F1000Res.

[84] Heinz S, Murre C, Cheng JX, Benner C, Spann N, Bertolino E (2010). Simple combinations of lineage-determining transcription factors prime cis-regulatory elements required for macrophage and B cell identities. Molecular Cell.

[85] Imakaev M, Fudenberg G, McCord RP, Naumova N, Goloborodko A, Lajoie BR (2012). Iterative correction of Hi-C data reveals hallmarks of chromosome organization. Nat Meth..

[86] Zhou X, Lowdon RF, Li D, Lawson HA, Madden PAF, Costello JF (2013). Exploring long-range genome interactions using the WashU Epigenome Browser. Nat Meth..

[87] Koohy H, Bolland DJ, Matheson LS, Schoenfelder S, Stellato C, Dimond A, et al. Genome organization and chromatin analysis identifies transcriptional downregulation of insulin-like growth factor signaling as a hallmark of aging in developing B cells. NCBI GEO. GSE109671. https://www.ncbi.nlm.nih.gov/geo/query/acc.cgi?acc=GSE109671. 10.1186/s13059-018-1489-y PMC6124017 30180872

