# Supplementary material for: Genome organization and chromatin analysis identify transcriptional downregulation of insulin-like growth factor signaling as a hallmark of aging in developing B cells
Source: Genome Biol. 2018 Sep 5;19:126. doi: 10.1186/s13059-018-1489-y (PMC6124017; doi:10.1186/s13059-018-1489-y)
Supplement: Supplementary file 2 — Supplementary Figures S1–S15. PDF of all supplementary figures S1–S15. (PDF 4237 kb) [file 13059_2018_1489_MOESM2_ESM.pdf]

Supplementary Figures

Genome organization and chromatin analysis identify transcriptional downregulation of insulin-like growth factor signaling as a hallmark of aging in developing B cells

Hashem Koohy[1,2,*], Daniel J. Bolland[1,3,*], Louise S. Matheson[1,3,*], Stefan Schoenfelder[1], Claudia Stellato[1], Andrew Dimond[1], Csilla Várnai[1], Peter Chovanec[1,3], Tamara Chessa[4], Jeremy Denizot[1,5], Raquel Manzano Garcia[1], Steven W. Wingett[1,6], Paula Freire-Pritchett[1,7], Takashi Nagano[1], Phillip Hawkins[4], Len Stephens[4], Sarah Elderkin[1], Mikhail Spivakov[1,8], Peter Fraser[1,9], Anne E. Corcoran[1,3,¶] and Patrick D. Varga-Weisz[1,10, ¶]

1 Nuclear Dynamics Programme, Babraham Institute, Cambridge
2 Human Immunology Unit, MRC Weatherall Institute of Molecular Medicine, University of Oxford, UK
3 Laboratory of Lymphocyte Signalling and Development, Babraham Institute, Cambridge
4 Signalling, Babraham Institute, Cambridge
5 Current address: Université Clermont Auvergne, Inserm U1071, M2iSH, USC-INRA 2018, F-63000 Clermont-Ferrand, France
6 Bioinformatics, Babraham Institute, Cambridge
7 Division of Cell Biology, Medical Research Council Laboratory of Molecular Biology, Cambridge CB2 0QH, United Kingdom
8 Functional Gene Control Group, MRC London Institute of Medical Sciences (LMS), Du Cane Road, London, W12 0NN, UK
9 Department of Biological Science, Florida State University, Tallahassee, Florida, USA
10 School of Biological Sciences, University of Essex, Colchester, UK
* These authors contributed equally to this work
¶ These authors contributed equally to this work

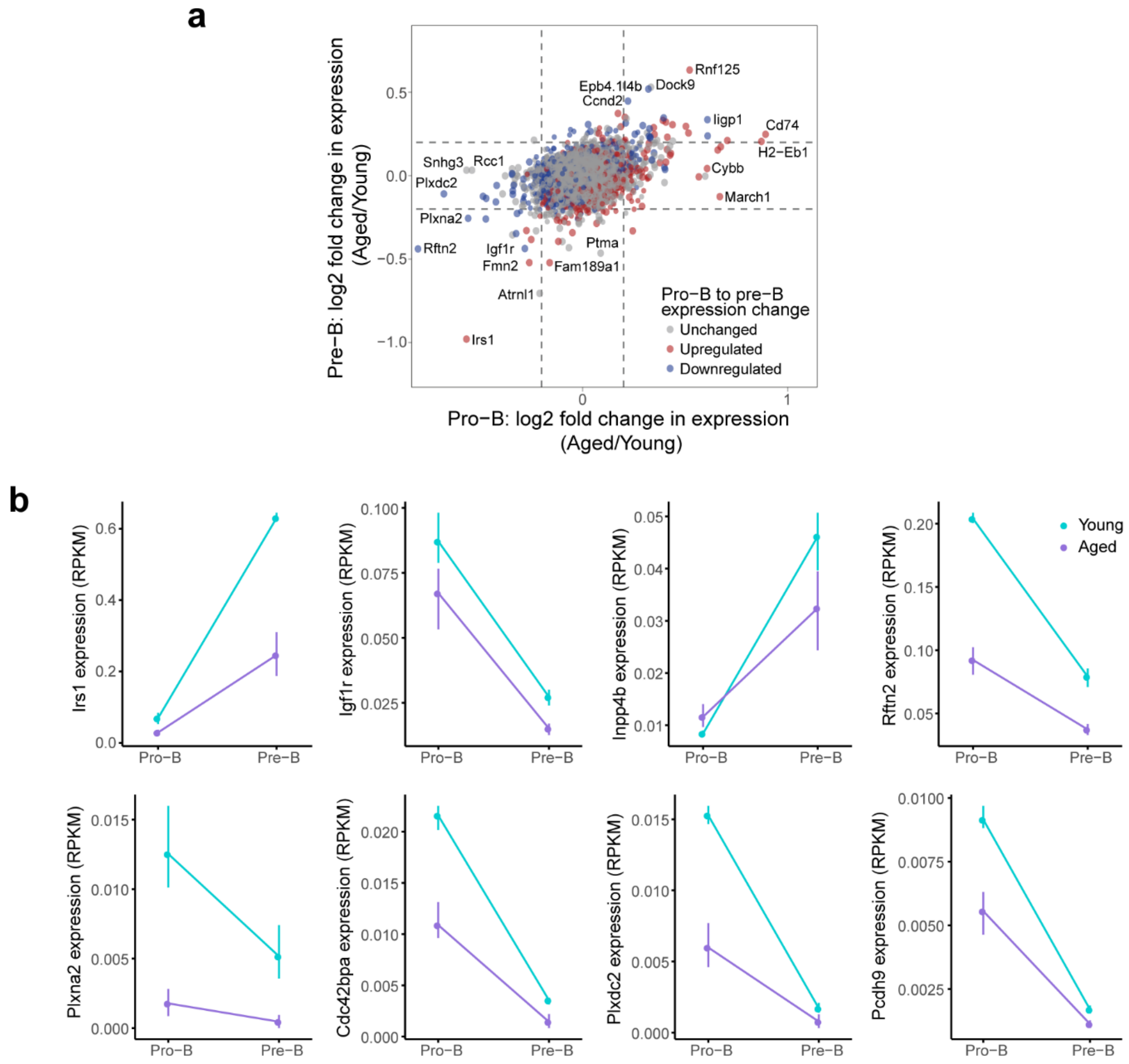

Fig. S1. Gene expression changes upon aging are linked to developmental changes in pro- and pre-B cells.

a Comparison of fold change in gene expression upon aging between pro-B and pre-B cells, showing a similar trend at both developmental stages. Genes with a significant (FDR adjusted *p* value < 0.05) age-related change in expression are indicated with larger point. Genes are colored based on whether their expression is significantly altered (FDR adjusted *p* value < 0.05 and absolute log2 fold change > 1) as cells progress from the pro-B to pre-B cell stage in young mice. Dashed lines indicate a log2 fold change of +/- 0.2. **b** Expression of selected age-downregulated genes in pro-B and pre-B cells. Read counts are expressed as reads per kb per million (RPKM). Points represent the mean, and vertical lines represent the range across three replicates.

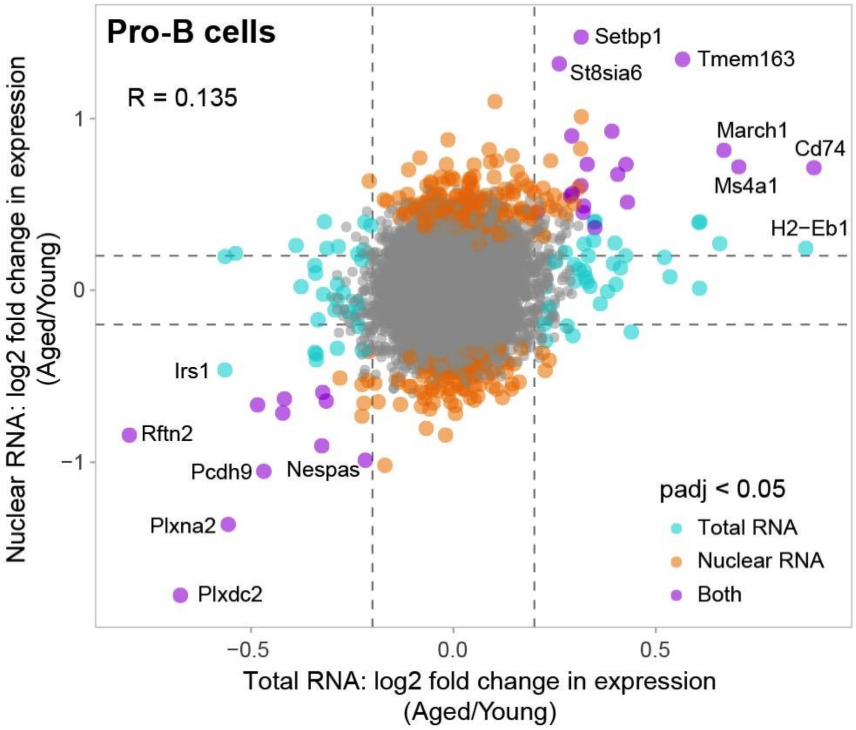

Fig. S2. Gene expression changes upon aging in pro-B cells in nuclear RNA compared to total RNA.

Comparison of fold change in gene expression upon aging between total and nuclear RNA analyses in pro-B cells, showing correlation between the changes detected in these two analyses. Genes with a significant (FDR adjusted *p* value < 0.05) age-related change in expression are indicated with larger point. Genes are colored based on whether their expression is significantly altered in the total RNA analysis, the nuclear RNA analysis, or both. Dashed lines indicate a log2 fold change of +/- 0.2.

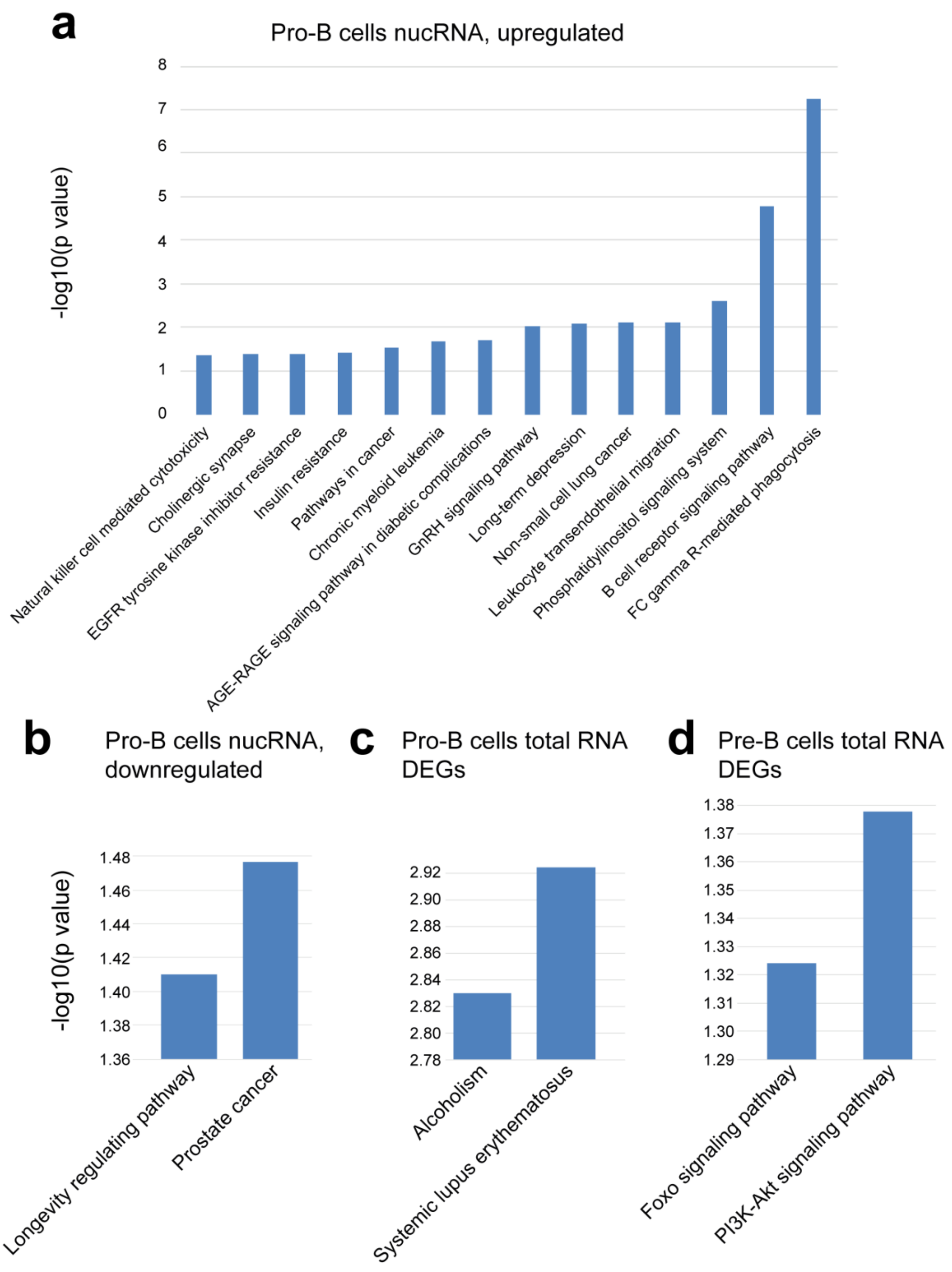

Fig. S3. KEGG pathway analysis of differentially expressed genes upon aging in developing B cells.
KEGG pathways which are significantly (padj < 0.05, Benjamini-Hochberg) associated to pro-B nuclear (nuc) RNA upregulated genes (**a**) and downregulated genes (**b**). Genes associated to the Longevity pathway include: *Igf1r, Igf1, Rb1cc1, Creb1* and *Kras*. Because of the low number of up- and downregulated genes identified in the total RNA-seq analysis, KEGG pathway analysis was performed on combined up- and downregulated genes in pro-B (**c**) and pre-B cells (**d**). Genes linked to the Foxo signaling pathway include *Ccnd2, Igf1r, Sgk1* and *Irs1.* Gene linked to the PI3K-Akt pathway include: *Ccnd2, Igf1r, Sgk1, Ccnd3*, *Reln* and *Irs1.* Mature signature B cell genes have been excluded from KEGG pathway analysis.

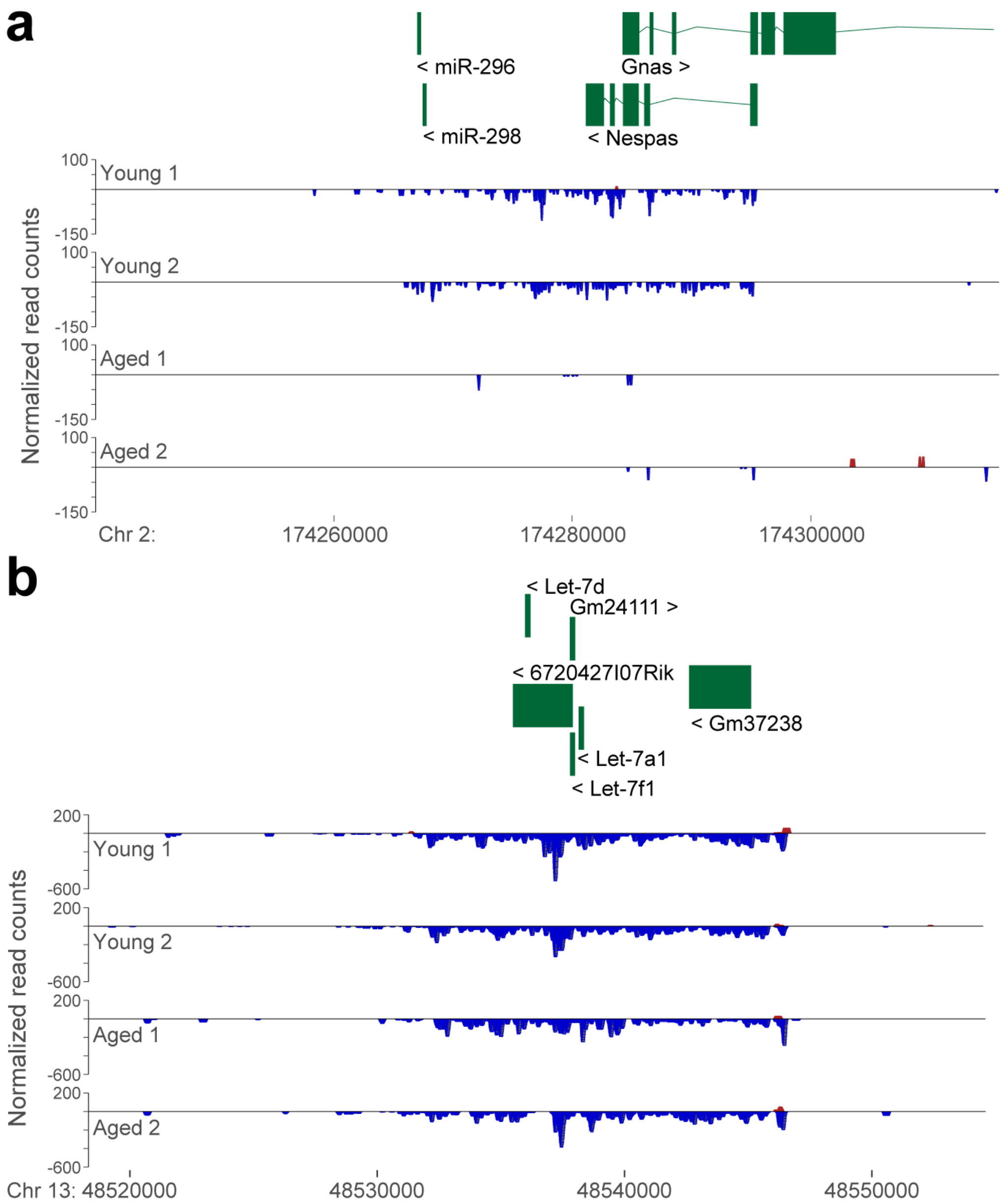

Fig. S4. Active transcription of miRNA precursors in pro-B cells.
Read counts for nuclear RNA-seq over (**a**) *Nespas*, the precursor transcript for *miR-296* and *miR-298*, and (**b**) a non-coding transcript encompassing *Let-7a1/d/f1* were quantified over 100-bp windows and normalized using DESeq2 size factors. Reads originating from the forward strand are represented as positive values (red), whilst those originating from the reverse strand are represented as negative numbers (blue). *Nespas* is significantly down-regulated in aged pro-B cells.

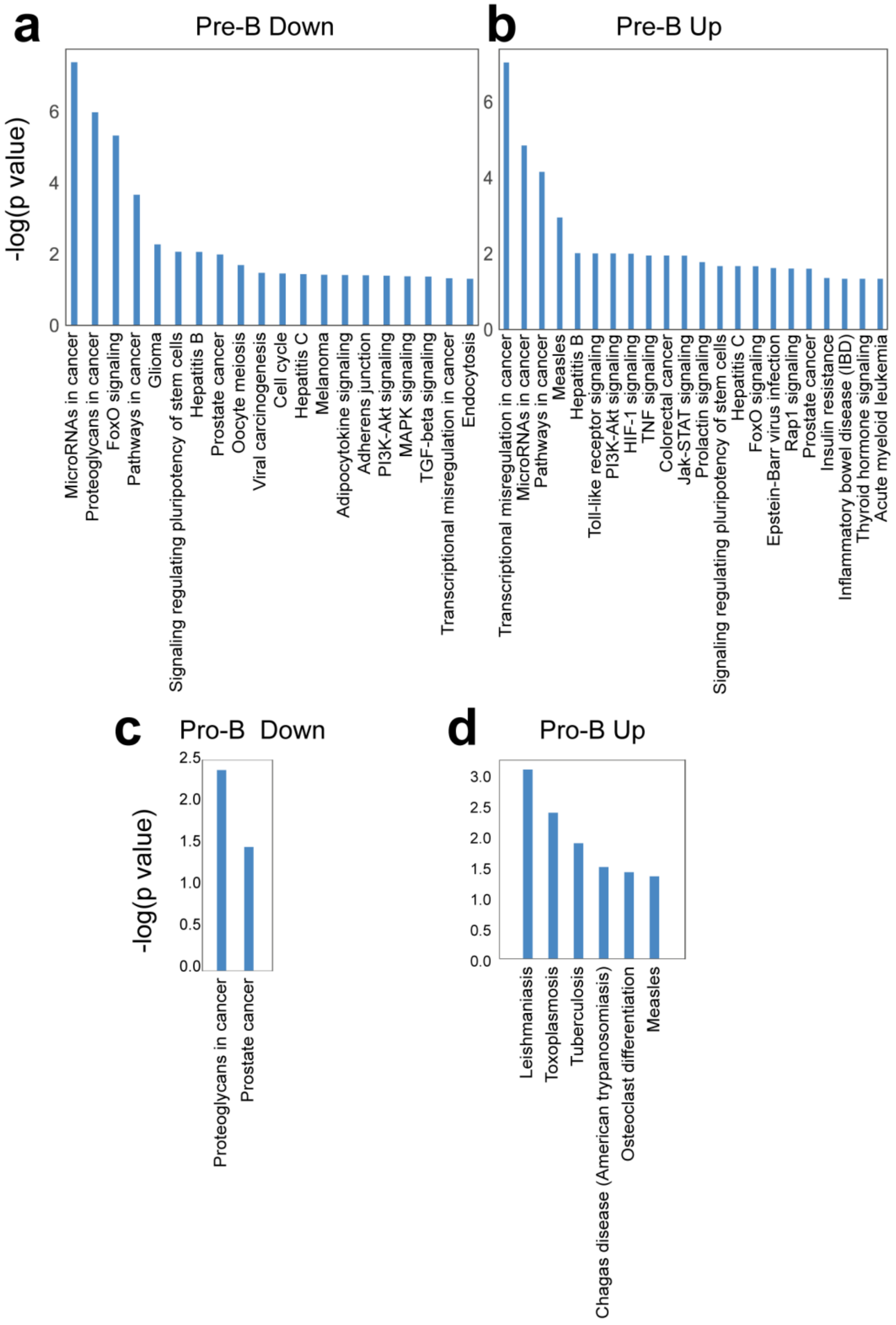

Fig.S5. KEGG Pathways associated with gene targets of differentially expressed miRNAs

Illustrated here are KEGG pathways which are significantly associated with target genes of differentially expressed miRNAs: of down-regulated miRNAs in pre-B cells (**a**), of up-regulated miRNAs in pre-B cells (**b**), of down-regulated miRNAs in pro-B cells (**c**) and of up-regulated miRNAs in pro-B cells (**d**). mirTarBase database was used for identification of functionally validated target genes (Additional File 1, Tables S13-S16). DAVID online server was used for KEGG pathway analysis of target genes. Please see Methods for full detail.

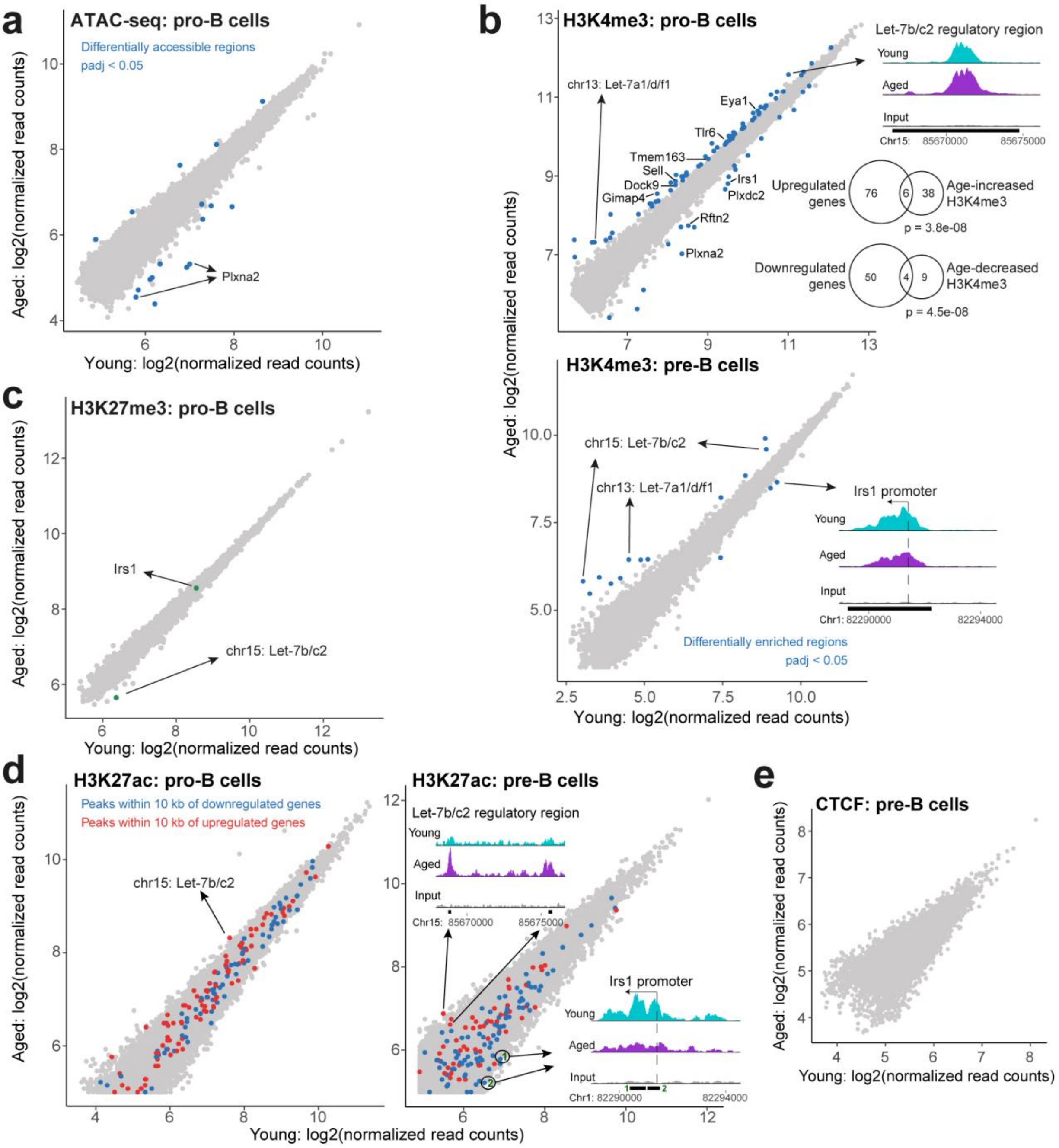

Fig. S6. Changes in chromatin features between young and aged B cell precursors. Scatter plots showing normalized read counts for each chromatin feature over MACS peaks for **a** ATAC-seq in pro-B cells, **b** H3K4me3 in pro- and pre-B cells, **c** H3K27me3 in pro-B cells, **d** H3K27ac in pro- and pre-B cells, **e** CTCF in pre-B cells. Blue points indicate differentially enriched regions defined using DESeq2 (FDR adjusted *p* value < 0.05), except for **d**, in which blue and red points indicate peaks within 10-kb of aged-down- and upregulated genes, respectively. Venn diagrams in **b** show overlap between differentially expressed genes (total RNA) and promoter regions with changes in H3K4me3 in pro-B cells. *P* values are based on a hypergeometric test; given the small number of differential genes/peaks and the large total number of genes from which they might originate, the overlaps are highly significant. Genes in the overlaps are indicated in the scatter plot. For inserts, median normalized reads for aged and young are shown on the same scale. Black bars beneath the plots indicate the peak locations.

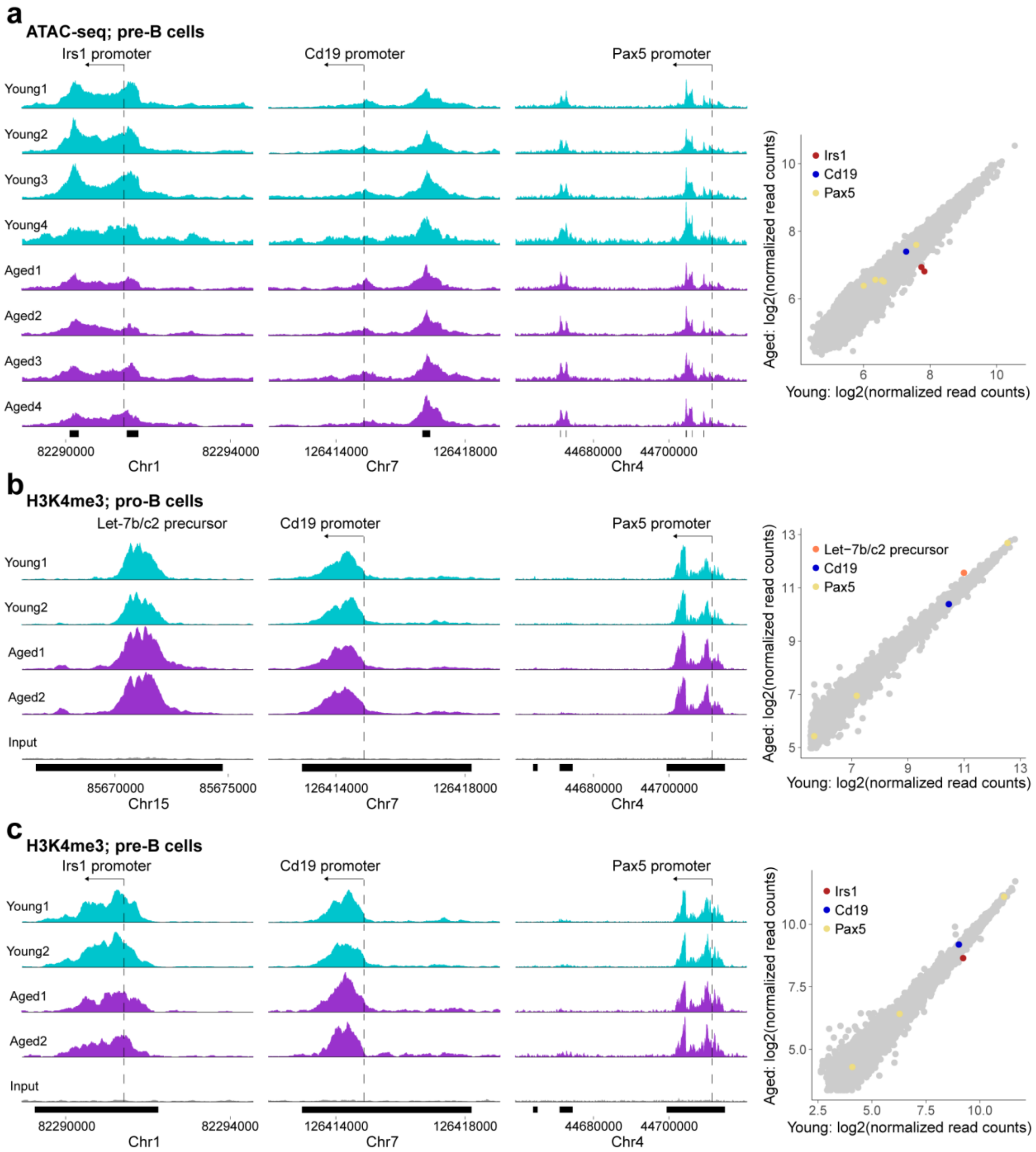

Fig. S7 ATAC-seq and H3K4me3 distributions over selected differentially expressed and control transcripts

a Left: Distribution of ATAC-seq reads in pre-B cells over *Irs1*, which is both differentially expressed and displays differential enrichment in ATAC-seq upon aging, compared to the control genes *Cd19* and *Pax5* which are unaltered. Right: Scatter plot highlighting the peaks over each locus. **b** Left: Distribution of H3K4me3 in pro-B cells over the *Let-7b/c2* precursor, which is both differentially expressed and displays differential enrichment in H3K4me3 upon aging, compared to the control genes *Cd19* and *Pax5* which are unaltered. Right: Scatter plot highlighting the peaks over each locus. **c** Left: Distribution of H3K4me3 in pre-B cells over *Irs1*, which is both differentially expressed and displays differential enrichment in H3K4me3 upon aging, compared to the control genes *Cd19* and *Pax5* which are unaltered. Right: Scatter plot highlighting the peaks over each locus. For all panels, read counts were normalized based on size factors from DESeq2 analysis of MACS peaks, and for a given dataset are displayed on the same scale for each replicate in young and aged mice, and across all three loci. Peaks are displayed as black bars below.

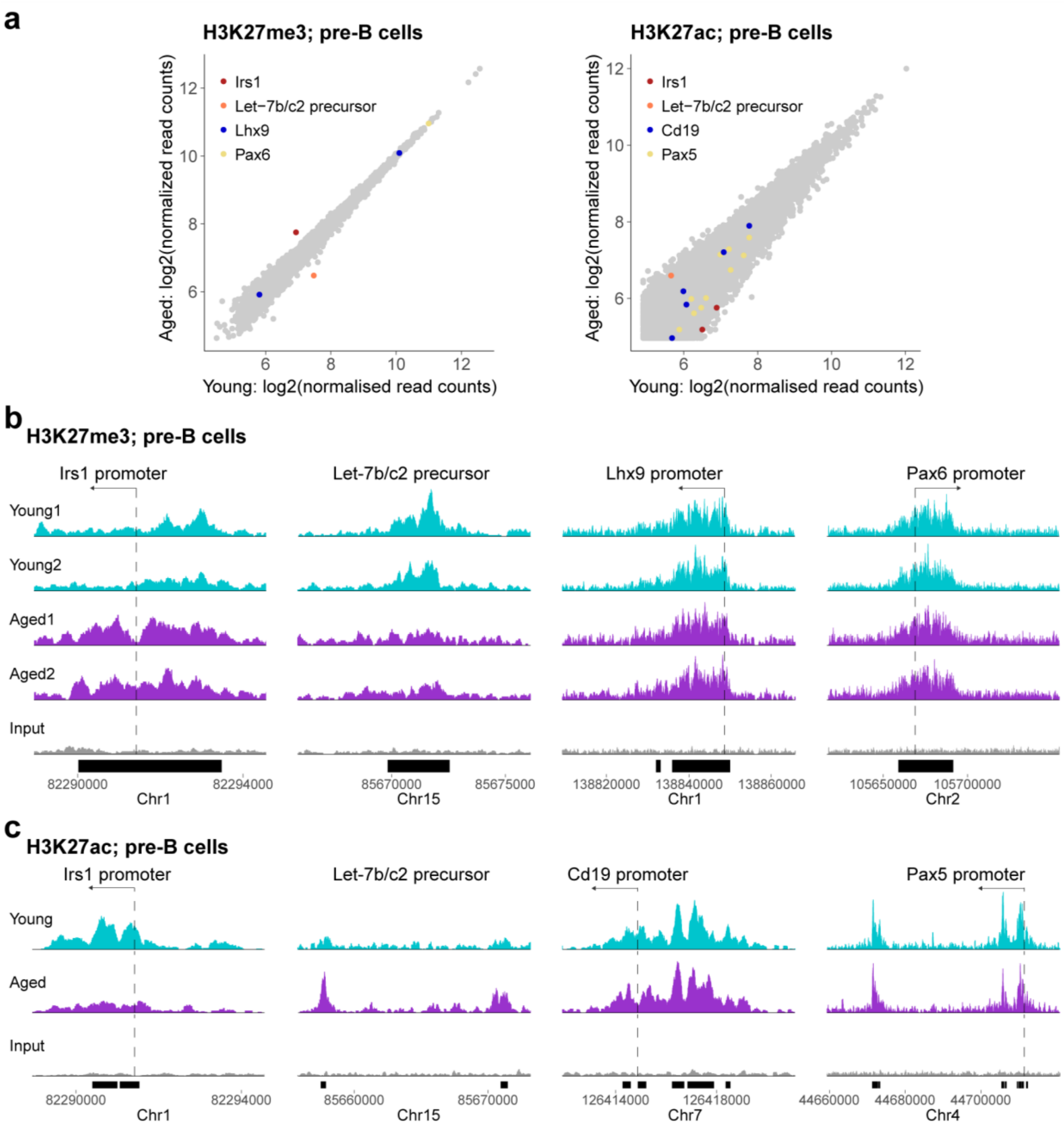

Fig. S8. H3K27me3 and H3K27ac distributions over selected differentially expressed and control transcripts in pre-B cells

a Scatter plots highlighting the peaks over selected transcripts for H3K27me3 (left) and H3K27ac (right). **b** Distribution of H3K27me3 in pre-B cells over *Irs1* and the *Let-7b/c2* precursor, which are both differentially expressed and display differential enrichment in H3K27me3 upon aging, compared to the control genes *Lhx9* and *Pax6* which are unaltered. Read counts were normalized based on size factors from DESeq2 analysis of H3K27me3 peaks, and are displayed on the same scale for each replicate in young and aged mice, and across all four loci. Peaks are displayed as black bars below. **c** Distribution of H3K27ac in pre-B cells over *Irs1* and the *Let-7b/c2* precursor, which are differentially expressed upon aging, compared to the control genes *Cd19* and *Pax5* which are unaltered. Read counts were normalized based on size factors from DESeq2 analysis of H3K27ac peaks, and are displayed on the same scale for young and aged mice, and across all four loci. Peaks are displayed as black bars below.

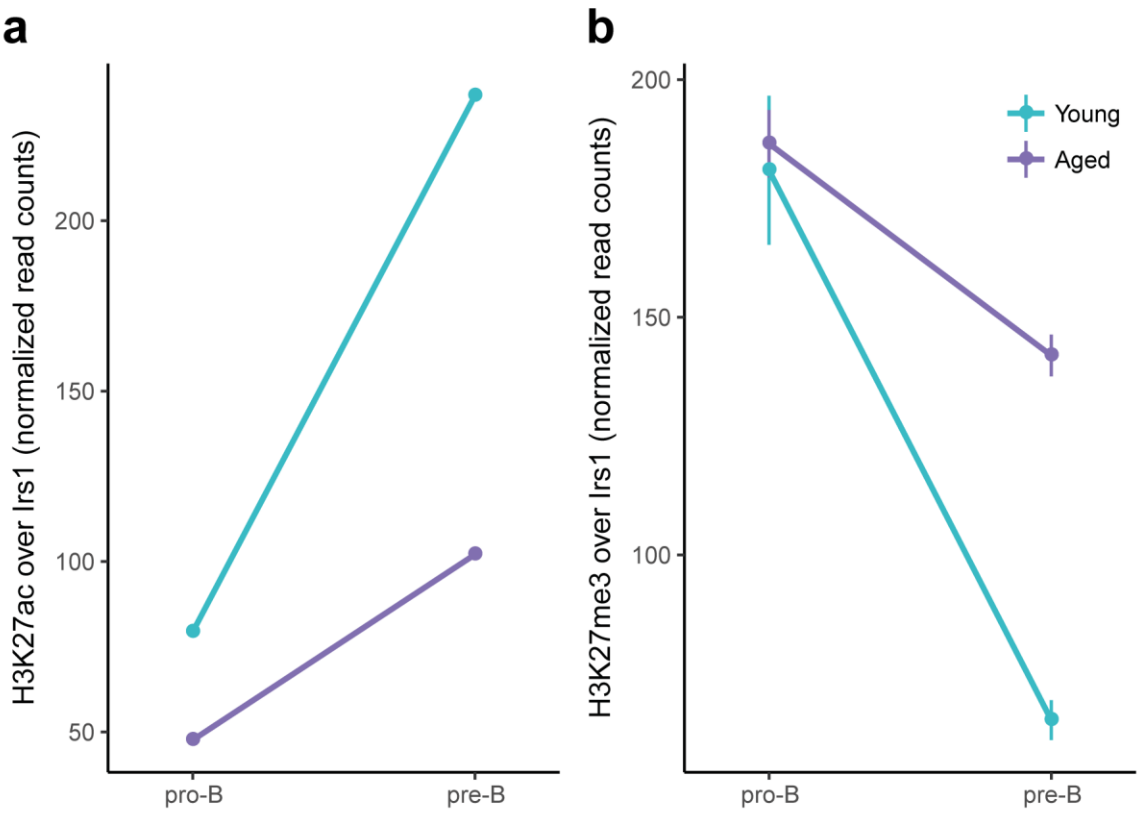

Fig. S9. H3K27me3 and H3K27ac show reciprocal changes upon aging and during B cell development.
Comparison of H3K27ac (**a**) and H3K27me3 (**b**) signal over the *Irs1* promoter in pro-B and pre-B cells from young and aged mice. Reads were quantified within a 2-kb window centered on the *Irs1* transcription start site, and normalized using size factors from DESeq2 analysis of MACS peaks. In **b**, points represent the mean and vertical lines represent the range.

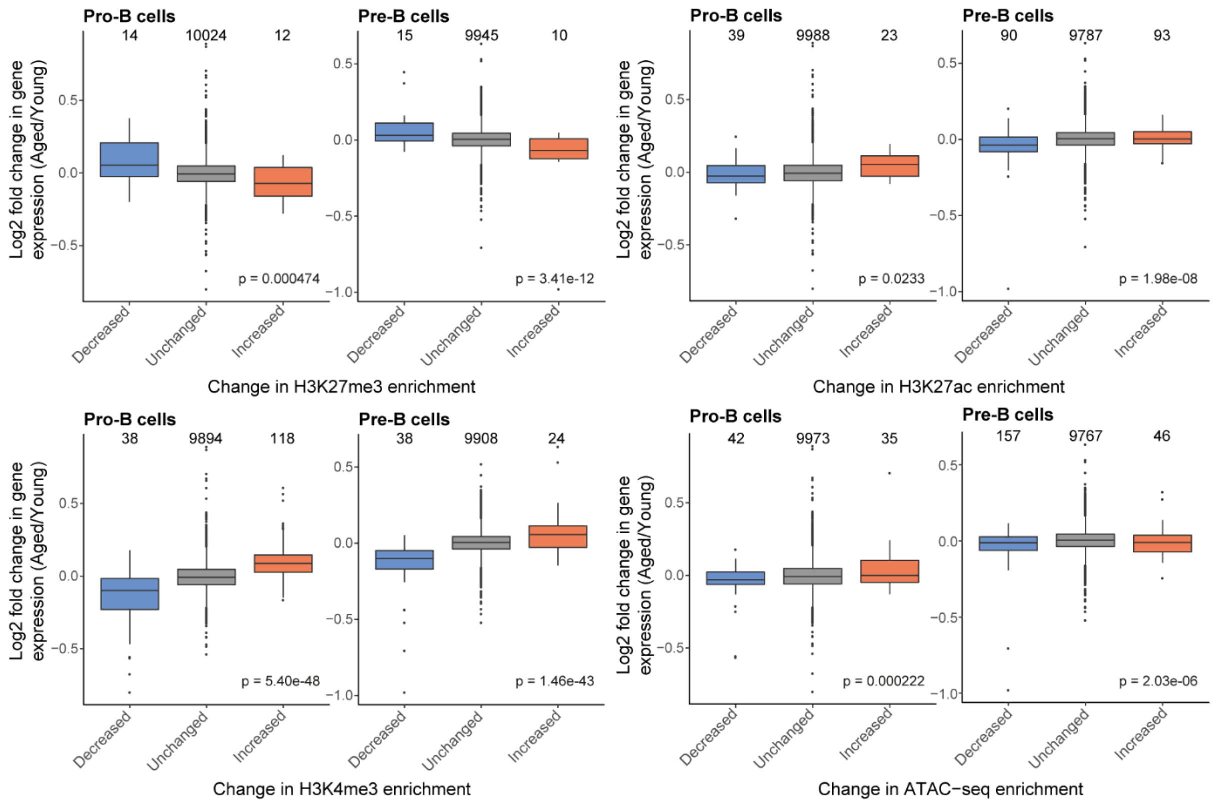

Fig. S10. Chromatin changes result in gene expression changes upon aging in B cell precursors.
Log2 fold change (Aged/Young) in the expression of genes close to peaks for H3K27me3, H3K27ac, H3K4me3 and ATAC-seq whose enrichment is altered (low stringency threshold: unadjusted *p* value < 0.1 for H3K27me3/H3K27ac or < 0.005 for H3K4me3/ATAC-seq), compared to genes close to peaks whose enrichment is unchanged. Peaks were assigned to a gene if they overlapped with a window extending 1-kb up- and downstream of the gene. ANOVA was used to determine *p* values; these were then FDR adjusted to correct for multiple testing. Numbers above each boxplot indicate the number of genes in each category.

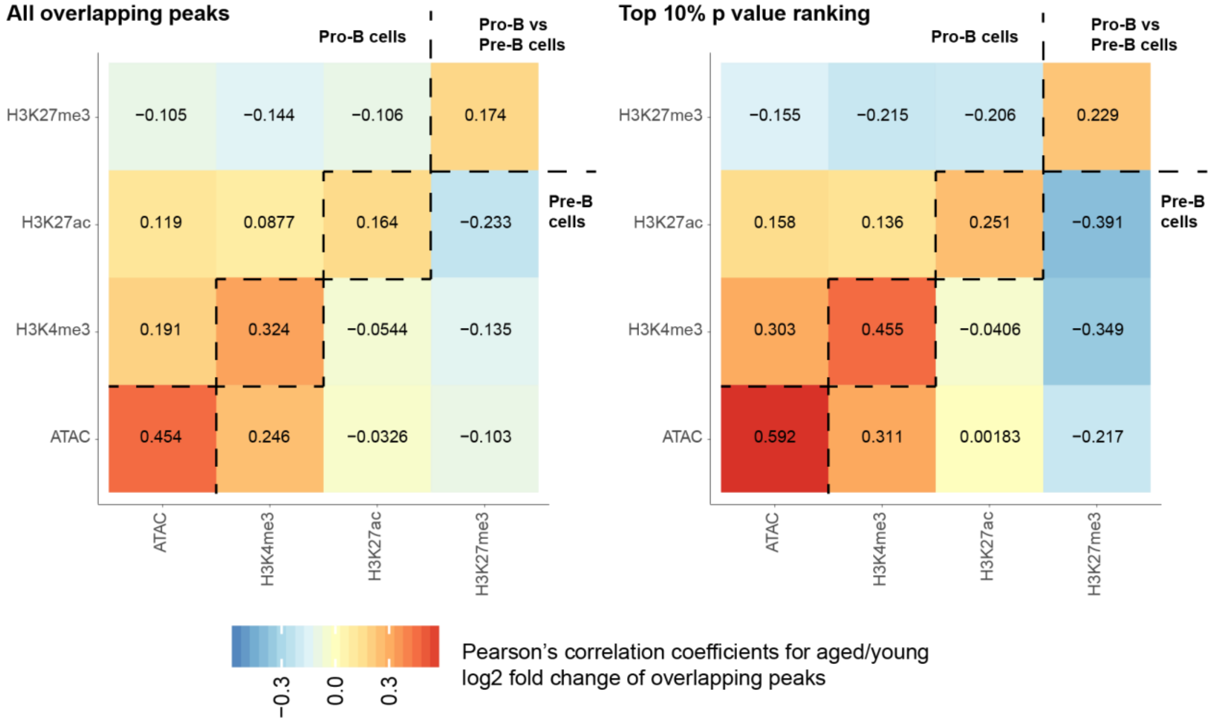

Fig. S11. The correlations of aged/young log-fold-changes between different chromatin features at overlapping peaks

Heatmaps showing the Pearson correlation coefficients between the aged versus young log2 fold changes for different chromatin analyses (ATAC-seq and ChIP-seq) in pro-B cells (above the diagonal), pre-B cells (below the diagonal), and between pro- and pre-B cells (on the diagonal). Left: Correlations for all overlapping peaks between a given pair of datasets. Right: Overlapping pairs of peaks were included only if at least one of them was ranked in the top 10% for differential signal between the respective young and aged cells. See Methods for details.

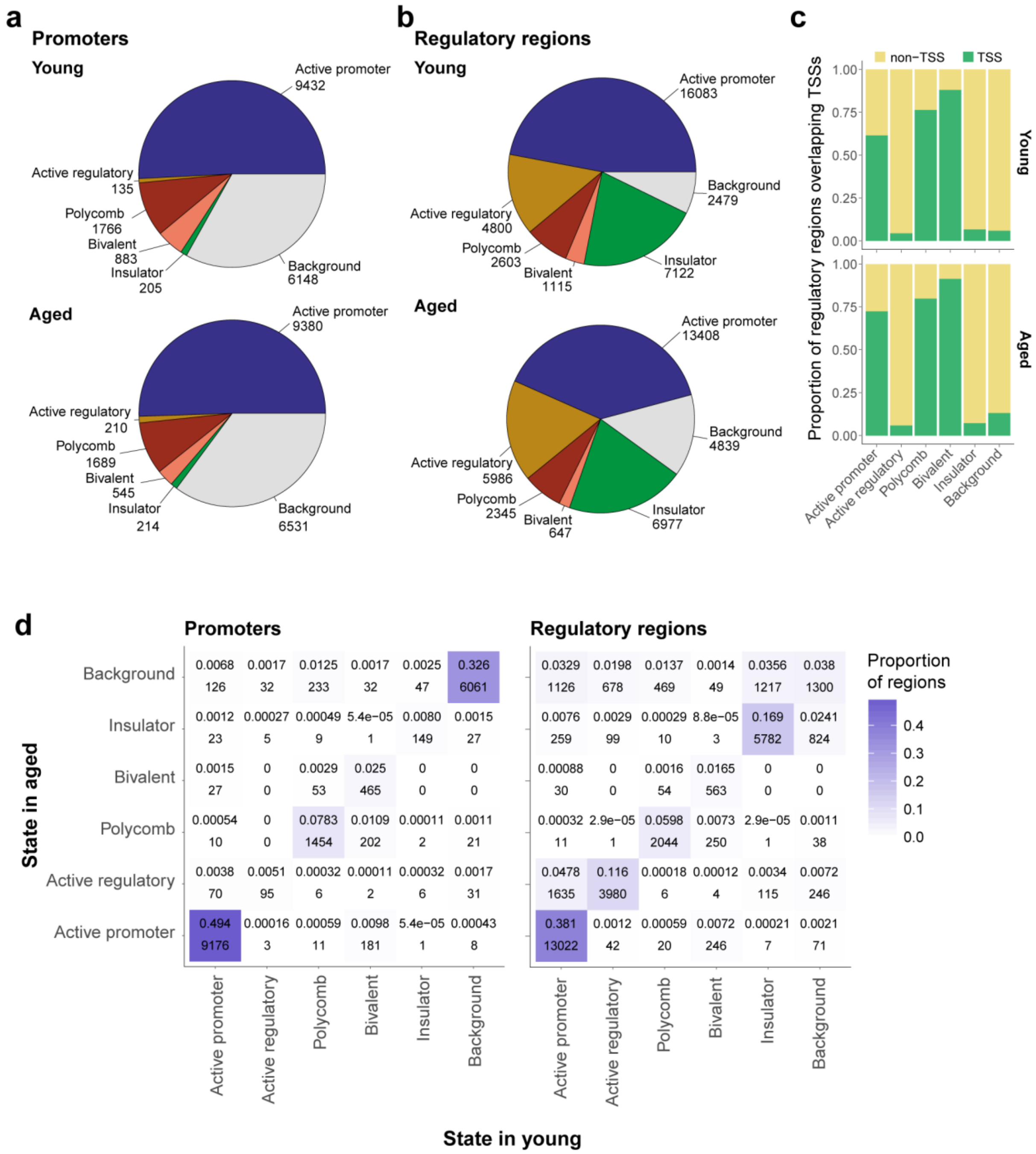

Fig. S12. **Global analysis of chromatin states**

a, b Pie charts showing the proportion of all promoters (**a**; 5-kb windows centered on the consensus transcription start site for all genes) or regulatory elements (**b**; defined as the union of peaks in ATAC-seq and ChIP-seq for H3K4me3, H3K27me3, H3K27ac and CTCF) that are in each state in pre-B cells. Numbers below the labels indicate the total number of promoters/regulatory regions assigned to each state. **c** Proportion of regulatory elements (as in **b**) that are within 1-kb of a transcription start site (TSS) for each state. **d** Heatmap showing the proportion of promoters (left) and regulatory regions (right) that are in the same state in young and aged pre-B cells, or transition from one state to another. Numbers in each square indicate the proportion (top) and total number (bottom) of regions for each transition.

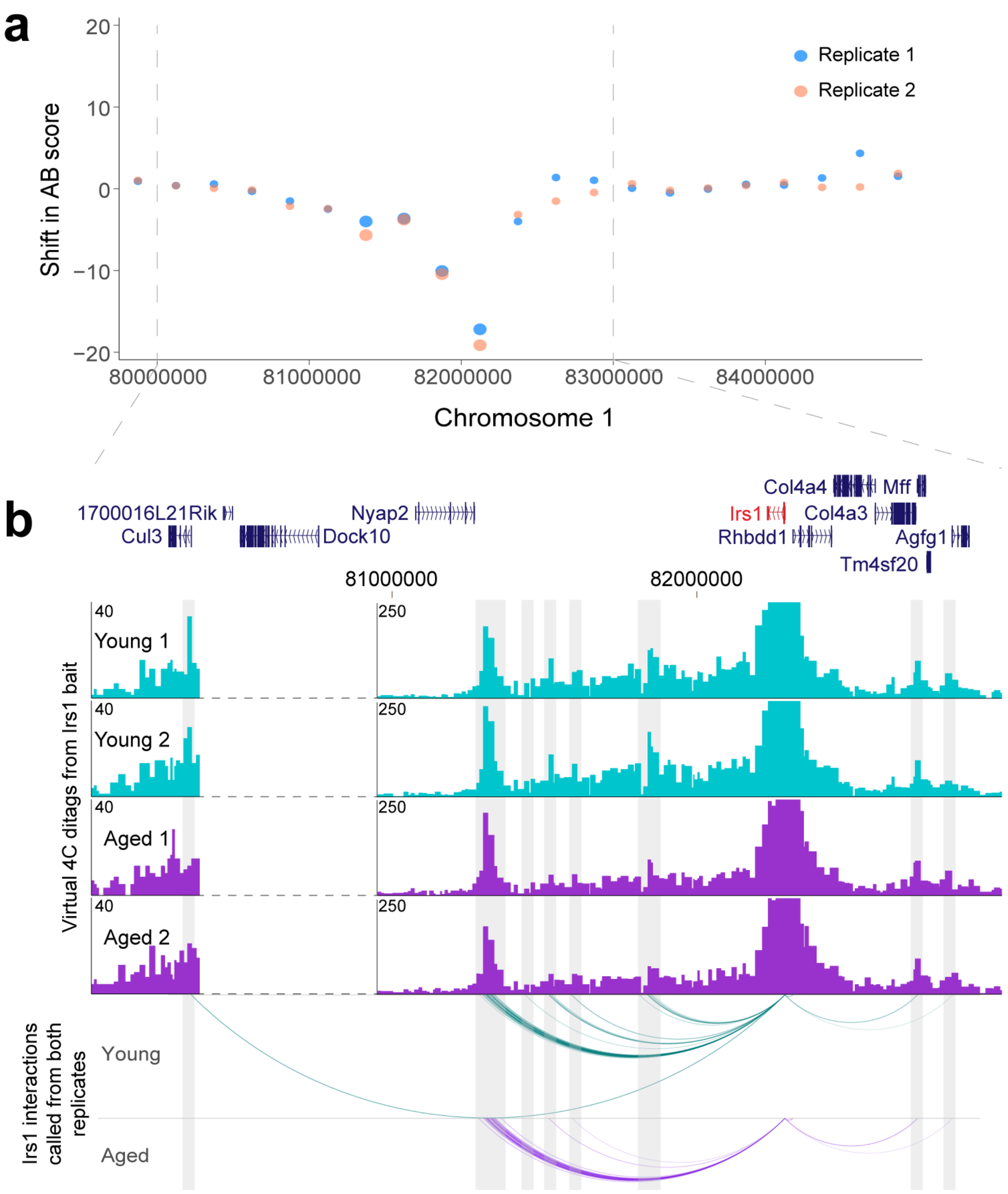

Fig. S13. Altered genomic organization at the *Irs1* gene locus
a Shift in Hi-C AB compartment strength between aged and young pre-B cells over a region encompassing the *Irs1* gene on chromosome 1, at 250-kb resolution. Replicates 1 and 2 are shown separately. Negative peak represents a shift away from the active A compartment and towards the inactive B compartment over the *Irs1* gene in aged mice; significantly altering regions are shown in with a larger point. **b** Virtual 4C for individual replicates showing read counts for the other end of all PCHi-C ditags that include the baited *HindIII* fragment encompassing the *Irs1* promoter at one end. Each quantitation window comprises five adjacent *HindIII* fragments. Gene locations are shown above, and significant interactions called by CHiCAGO are shown below. Ditag read counts are displayed on two different scales to better display the data, due to the exponential decay as you move further from the bait that is characteristic of Hi-C based approaches.

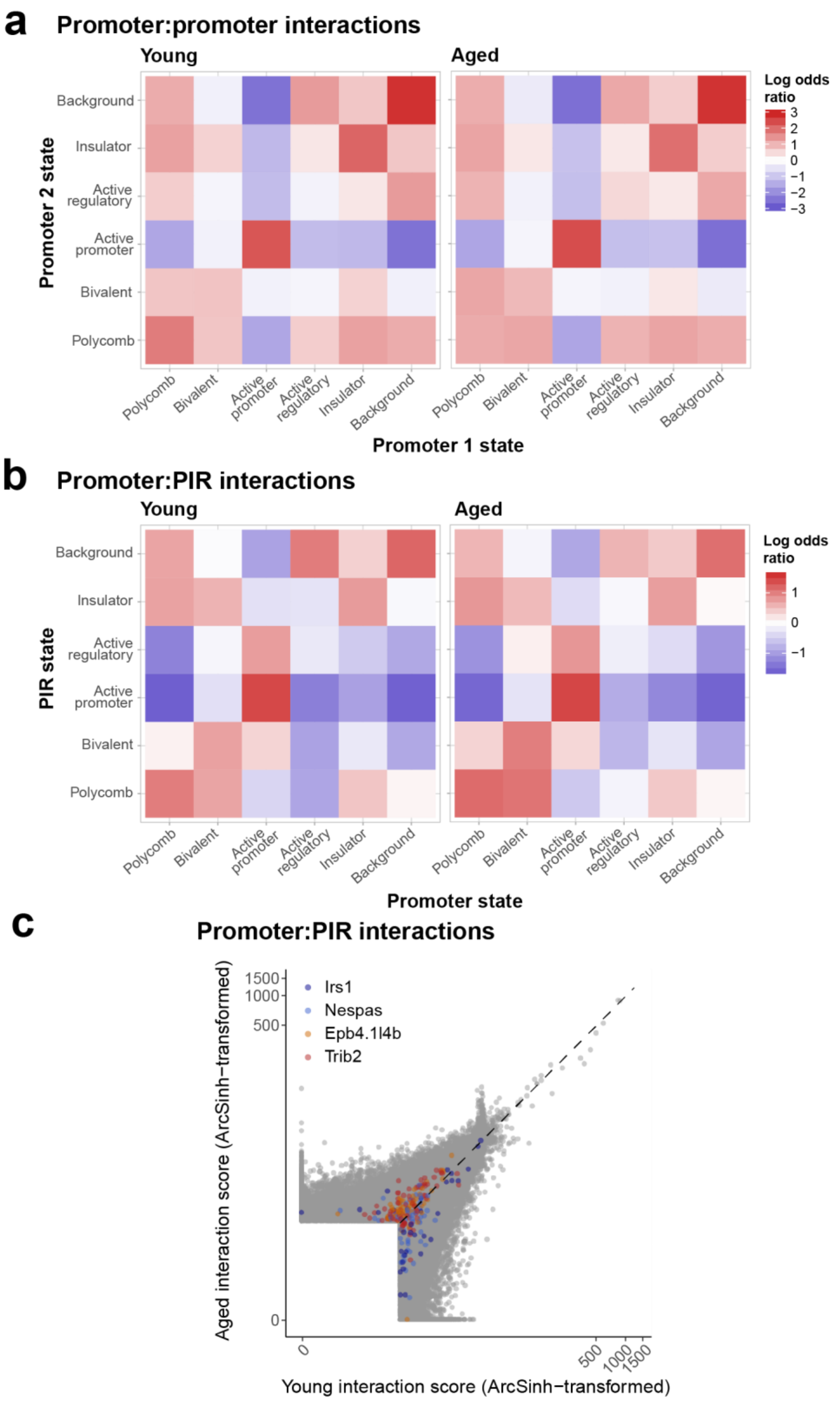

Fig. S14. Genome organization changes in pre-B cells upon aging.
a, b Log2 odds ratios for the number of promoter:promoter (**a**) and promoter:PIR (**b**) interactions comprising each combination of chromatin states, compared to what would be expected by chance. **c** CHiCAGO interaction scores in young and aged pre-B cells for all promoter:PIR interactions, with selected downregulated (*Irs1* and *Nespas*) and upregulated (*Epb4.1l4b* and *Trib2*) genes highlighted. All scores have been ArcSinh transformed; dashed line indicates an identical score for young and aged.

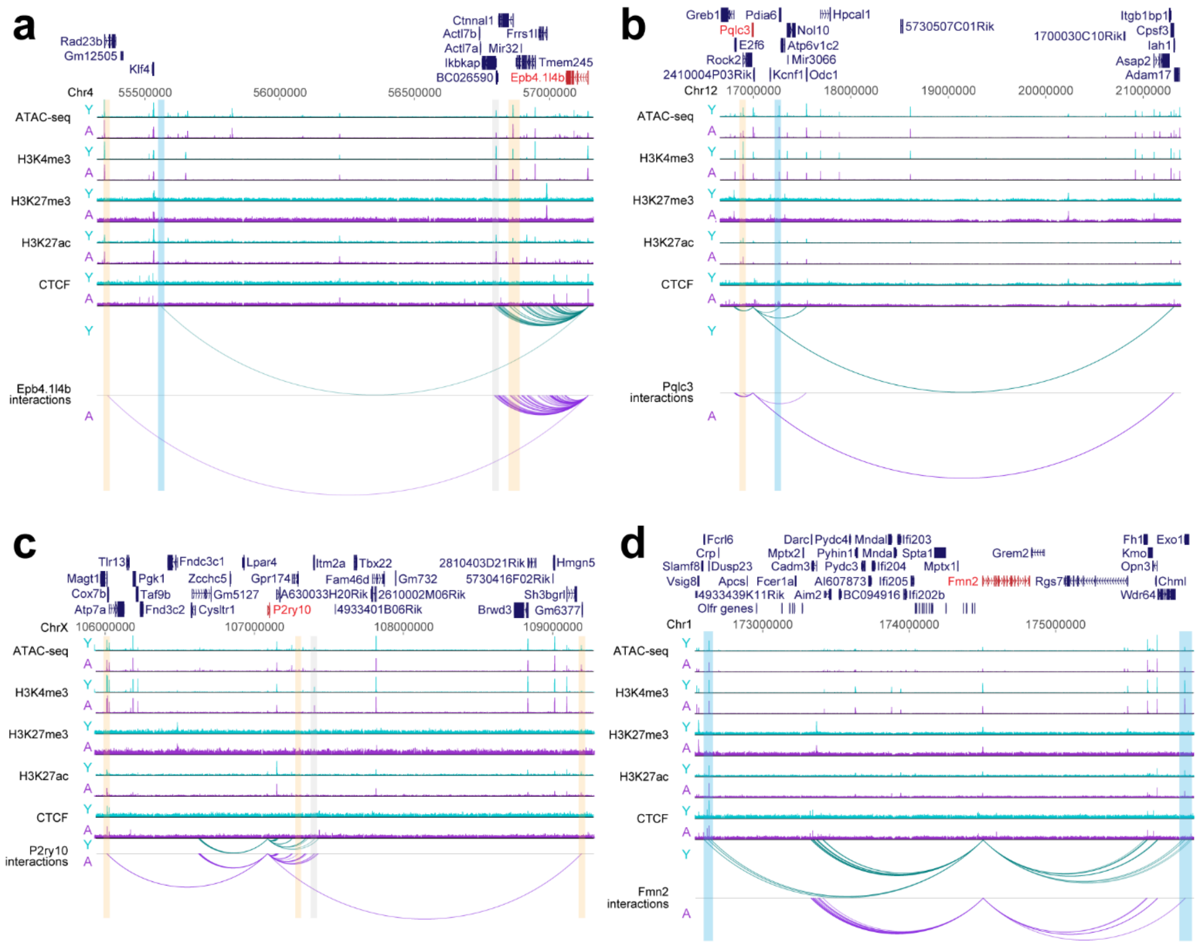

Fig. S15. Rewiring of promoter interactions in aged pre-B cells

Genome browser representation of interactions in young and aged pre-B cells from the promoters of genes whose expression is upregulated (*Epb4.1l4b* (**a**), *Pqlc3* (**b**) and *P2ry10* (**c**)) or downregulated (*Fmn2* (**d**)) upon aging. Shown are all interactions with a CHiCAGO score above the significance threshold of 5 that lie within the genomic region depicted. For ATAC-seq and ChIP-seq tracks, read counts are quantified over 100-bp windows and normalized with size factors from DESeq2 analysis of MACS peaks. For a given locus and chromatin mark, young and aged are displayed on the same scale. Shading indicates regions that gain interactions (orange), lose interactions (blue), or over which active histone modifications are increased (grey).
