# Supplementary material for: Genome organization and chromatin analysis identify transcriptional downregulation of insulin-like growth factor signaling as a hallmark of aging in developing B cells
Source: Genome Biol. 2018 Sep 5;19:126. doi: 10.1186/s13059-018-1489-y (PMC6124017; doi:10.1186/s13059-018-1489-y)
Supplement: Supplementary file 5 — Reviewer reports and authors’ response to reviewers. (DOCX 119 kb) [file 13059_2018_1489_MOESM5_ESM.docx]

**First round of review**

**Reviewer 1**

**Are the methods appropriate to the aims of the study, are they well described, and are necessary controls included?**
Yes.

**Are the conclusions adequately supported by the data shown?**

Yes.

**Are sufficient details provided to allow replication and comparison with related analyses that may have been performed?**

Yes

**Does the work represent a significant advance over previously published studies? Please explain.**

Yes. First demonstration that insulin signalling pathway is affected in aged pro-B cells and caused by polycomb mediated repression.

**Is the paper of broad interest to others in the field, or of outstanding interest to a broad audience of biologists?**

Yes.

**Comments to authors**: Previous studies have established that aged mice display significant abnormalities in the bone marrow. In particular, the frequencies bone marrow B cell precursors are affected during ageing. Here a comprehensive and detailed analysis of gene expression and epigenetic marks associated with ageing B cell progenitors was performed. The authors find a narrow set of genes that are differentially expressed in B cell progenitors. Specifically, the authors identify member of the insulin-like growth factors signaling pathways as key genes whose expression is modulated in aged mice. The authors provide evidence that Polycomb group proteins underpin the differential expression of members of the insulin-like pathway in aged bone marrow B cells.

This is an excellent study. The data are comprehensive, detailed and convincing. While previous studies have attempted to address the topic of aging B cell progenitors in the bone marrow these studies are in the opinion of this reviewer the first to provide solid and believable information. I strongly recommend publication of this manuscript in Genome Biology.

Comments
The authors performed RNA-seq (ribosomal RNA-depleted) on pre- and pro-B-cells isolated from the bone marrow of young and aged mice (Figure 1). The cells were sorted by FACS and defined by CD19, B220, CD25, IgM, CD43. T-SNE provides a good overall view of the data but it is difficult to appreciate the changes quantitatively. They might want to consider moving the plots from the supplementary data to the main figures.

The authors discuss some of the data prematurely in the results section. It would be better to move this to the discussion section.

**Reviewer 2**

**Are the methods appropriate to the aims of the study, are they well described, and are necessary controls included?**
Yes, some important details are needed for some analyses.

**Are the conclusions adequately supported by the data shown?**

Yes in general.

**Are sufficient details provided to allow replication and comparison with related analyses that may have been performed?**

Yes in general.

**Does the work represent a significant advance over previously published studies? Please explain.**

Yes. Little is known about epigenomic changes with aging. This is a useful and comprehensive resource.

**Is the paper of broad interest to others in the field, or of outstanding interest to a broad audience of biologists?**

Yes. Interests both aging biologists, immunologists.

**Comments to authors:** This is a comprehensive "omics" study to uncover changes in B cells (Pro and Pre) with aging. The authors generated extensive data and studied different aspects of transcription and epigenetic regulation with aging in these two cell types. They uncovered that changes with aging are minimal and specifically targeting a few genes and insuling signaling pathway. Changes in these genes have been confirmed with multiple assays. The extent of data generated for this project is impressive and very informative, since very little is known in terms of epigenetic changes in immune cells with aging, beyond DNA methylation studies. The work and the data will be a great resource for the community. There are certain analyses that need clarification and certain analyses that are missing. Our specific suggestions/questions are listed below:

Major points:
* A supplementary table for basic stats of omics assays is needed: read depth, peak number, etc. It is not clear whether some of the observed changes can be attributable to QC as is specifically for chromatin interactions. 

* Need to discuss what is known in terms of epigenomics of immune cells with aging in the background beyond DNA methylation: http://jem.rupress.org/content/early/2017/09/12/jem.20170416 
http://immunology.sciencemag.org/content/2/8/eaag0192

* In general figure legends and figure panels can be improved to help the reader. For example, Figure 1A, Sup figure 1b,c,d can you please add the name of the cells (pro/pre-B) on panels. Hard to follow with the legend. Many important details are omitted. Would be useful to provide the crucial details (e.g., number of animals used for each genomic assay for example) in figure panels and legends. Specific examples: Figure 1B, C, how many mice is used for these analyses? How many mice for figure 2A,C,D. 
* Figure 2D, the clustering results are confusing. What does the last cluster represent, it doesn't change with aging? Please add simple explanation next to each cluster. Authors should show the expression heatmap from all used individuals here, instead of normalizing the groups based on the median. With this change we can see the number of replicates and see whether there are outlier animals.

* Figure S3, are these p-values corrected for multiple hypothesis testing?

* Figure 3A and B, do genome browser inserts have the same scale? This is not mentioned in the legend and is a critical information. I suggest authors to show the peaks as black bars under bigwig profiles for these figures instead of just an arrow that seems to be randomly pointing to this locus. For these figures, authors should also show examples of loci that are not differential for comparison purposes. Also, are these averages of multiple animals? Can they show the data for all biological replicates for these two loci, maybe in Sup?

* FC/FC correlations should be discussed for the following: ATAC vs H3K4me3 (H3K27ac) and H3K4me3 vs H3K27ac

* Why does K4Me3/K27ac agrees more with DE than ATAC? Figure 4 and S7? Are all distal peaks used in these analyses? Would the results get better if only promoter peaks are considered for all assays? Need to be discussed in the paper. 

* What is Figure 5b is showing. Correlation between emission probabilities for pairs of states? Please clarify. 

* Did the authors generate ChromHMM states separately in old and mice? This needs further clarification and they need to show emission probabilities for old and young in 5A is this is the case. Also a global comparison is needed? Are there similar number of promoters, enhancers, etc in old and young? What portion of regulatory elements switch in activity with aging ? In general figure 5D doesnot show major changes for most genes. Can the authors speculate why? 

* What is the difference between Figure S8a and b? Clarification needed. 

* Are there significant changes in chromatin interactions at the network level with aging? Any hubs specific to age groups? Are networks less/more connected? Basic stats for these data should be discussed. 

* Can the lost interactions in Figure 7a be due to the depth of chromatin interaction assay since there are not any other epigenomic changes in the marked regulatory elements? Can they show the 2 replicates for the promoter-capture Hi-C and Hi-C assays separately to show this is consistent across reps. Can authors test these specific interactions using 3C or 4C if they are claiming that these changes are real and consequential?

* Does PIRs include promoter-promoter interactions? What is the rationale for splitting the data into two like this? 

* How are the p-values for Figure 6c,f are calculated, because numbers do not seem that different ? Are they testing whether there is a change? The direction also needs to be tested in these and the details of the test should be provided. 

* How do the authors calculate A and B chromatin scores. Some details on this is needed in the methods. 

* Figure 6e, what is the goal of this analysis? why is there no association with epigenome and transctiptome in this analyses, despite the fact that association has been observed in global analyses. Is it due to small gene numbers tested? Please also clarify the gene numbers used in each analyses. 

* Figure 5C, this analysis is confusing. First details are missing, second why do the authors believe it is useful to look at the chromatin accessibility of promoters for regions that are not promoters (i.e., insulator, Res, polycomb). This analysis should be excluded and the gene expression analyses should be concluded on the chromatin states directly instead of the clusters drived from this analysis. 

* Figure 5D, Authors need to compare all down/up regulated genes and whether there is more active promoter bps in aged (young) for up(down) regulated, maybe using a chi-square test.

Minor points:

* Fold Change (FC) correlation between total and nascent would be useful to understand to what extent they agree. 

* Venn diagrams in Figure S5b are not exciting? Is this because H3K4me3 peaks are also found at enhancers? Or did they already restrict it to promoter peaks? The authors suggest that this is a significant overlap based on chi-square p-values, but it will be more convincing to see the fold changes agree for these sites in both assays. 

* Results from transcriptome are interesting, however, more analyses could be conducted to make sense of these changes for biology. I suggest using GSEA analyses for DEG genes both for total and nascent RNA, which might uncover pathways, gene sets that are up/down regulated as a group. Also, to further interpret the differential microRNAs authors can also incorporate their target genes using computational methods (http://www.mirdb.org/). If same pathway or genes are targeted by these, this will be extra evidence. 

* How would all the epigenomic data compare to each other if the authors put these into a single plot like, PCA or tSNE. Would be great to see this. There are some technical things to consider (like how to define peaks across assays etc), however, this can make a nice overall figure for these epigenomic assays. 

**Authors’ response to reviewers**

*Reviewer #1:*

*Previous studies have established that aged mice display significant abnormalities in the bone marrow. In particular, the frequencies bone marrow B cell precursors are affected during ageing. Here a comprehensive and detailed analysis of gene expression and epigenetic marks associated with ageing B cell progenitors was performed. The authors find a narrow set of genes that are differentially expressed in B cell progenitors. Specifically, the authors identify member of the insulin-like growth*

*factors signaling pathways as key genes whose expression is modulated in aged mice. The authors provide evidence that Polycomb group proteins underpin the differential expression of members of the insulin-like pathway in aged bone marrow B cells.*

*This is an excellent study. The data are comprehensive, detailed and convincing. While previous studies have attempted to address the topic of aging B cell progenitors in the bone marrow these studies are in the opinion of this reviewer the first to provide solid and believable information. I strongly recommend publication of this manuscript in Genome Biology.*

*Comments*

*The authors performed RNA-seq (ribosomal RNA-depleted) on pre- and pro-B-cells isolated from the bone marrow of young and aged mice (Figure 1). The cells were sorted by FACS and defined by CD19, B220, CD25, IgM, CD43. T-SNE provides a good overall view of the data but it is difficult to appreciate the changes quantitatively. They might want to consider moving the plots from the supplementary data to the main figures.*

**Response 1. We have followed this suggestion and now present Figure 1a combined with what was supplementary Figure S1 as the first main figure.**

*The authors discuss some of the data prematurely in the results section. It would be better to move this to the discussion section.*

**Response 2.**

**We thank the reviewer for highlighting this and have accordingly moved the following to the Discussion section, either intact, or merged with similar content in the appropriate section therein:**

**Line 168, page 7: “Several miRNAs, including the Let-7 miRNA family, which regulates Irs1, have been implicated in the ageing process [21].”**

**Line 174: “We also observed an upregulation of seven Let-7 family members and of miR-223, which modulates Igf1r expression [22,23], upon ageing in pro- and/or pre-B cells” - underlined phrase has been moved to Discussion.**

**Line 185: “Let-7 miRNAs have been linked to the ageing process [25].”**

**The above sentences have been moved to the following section of the Discussion:**

**Precursor B cell ageing is associated with gene expression alterations linked to IGF signalling, line 496-498**

**Line 193: “including several that co-ordinately regulate metabolic signalling pathways”**

**Line 269: “In summary, these analyses uncovered a role for chromatin remodelling in underpinning gene expression changes upon ageing in precursor B cells.”**

**These sentences have been deleted, as they are repetitive with statements already in the Discussion.**

**Line 343: “Our analysis highlighted in particular Irs1 and Let-7b and -7c2 as differentially regulated genes with substantial chromatin/epigenetic changes at their promoter/regulatory regions in aged pre-B cells. Notably, these genes are key players in the insulin/IGF and nutrient signalling pathways and their downregulation has been associated with the ageing process.” The underlined sentence has been moved to the following section of the Discussion: Precursor B cell ageing is associated with gene expression alterations linked to IGF signalling, line 494-495, and the other sentence has been deleted.**

**Line 408-412: shortened to make summary more results focussed.**

*Reviewer #2:*

*This is a comprehensive "omics" study to uncover changes in B cells (Pro and Pre) with aging. The authors generated extensive data and studied different aspects of transcription and epigenetic regulation with aging in these two cell types. They uncovered that changes with aging are minimal and specifically targeting a few genes and insulin signaling pathway. Changes in these genes have been confirmed with multiple assays. The extent of data generated for this project is impressive and very informative, since very little is known in terms of epigenetic changes in immune cells with aging, beyond DNA methylation studies. The work and the data will be a great resource for the community. There are certain analyses that need clarification and certain analyses that are missing. Our specific suggestions/questions are listed below:*

*Major points:*

** A supplementary table for basic stats of omics assays is needed: read depth, peak number, etc. It is not clear whether some of the observed changes can be attributable to QC as is specifically for chromatin interactions.*

**Response 3. We apologise for incompletely providing this basic information in our initial submission. To address this, we have expanded Table S1 in Additional file 2, which now includes the total read counts for all datasets, the numbers of peaks for ChIP-seq and ATAC-seq datasets, and several summary statistics for the Hi-C and Promoter Capture-Hi-C analyses. To highlight the consistency of signals between different replicates, we also now show browser views of selected tracks of replicates for some of the key ATAC-seq and ChIP-seq data, including loci that do not show changes (new supplementary Figures S7 and S8), and also Hi-C and Promoter Capture Hi-C data (new supplementary Figure S13).**

** Need to discuss what is known in terms of epigenomics of immune cells withaging in the background beyond DNA methylation:*

*http://jem.rupress.org/content/early/2017/09/12/jem.20170416*

*http://immunology.sciencemag.org/content/2/8/eaag0192*

**Response 4. We have now expanded the introduction, taking the suggestions of this reviewer into consideration. We now write (page 4, line 85-95, new parts in red): “The advent of new technologies in functional genomics enables illumination of the changes in B cell development that occur during ageing genome-wide. Recently, application of these technologies to ageing human T cells has provided profound insight into widespread epigenetic changes that impair the function of closely related lymphocytes in the adaptive immune system. In particular, ageing CD8+ T cells lose chromatin accessibility at promoters, which may compromise their metabolic state [14 – Moskowitz et al Sci Immunol 2017]. Comparison of ageing human lymphocyte populations in peripheral blood has revealed concordant downregulation of chromatin accessibility and gene expression of components of the IL7R signalling pathway in CD8+, but not CD4+ T cells, suggesting that epigenetic dysfunction in shared pathways in ageing requires careful unravelling [15 – Ucar et al JEM 2017]. Here we present an integrated analysis of the transcriptome, epigenetic landscape and higher-order chromatin structure in young and aged pro- and pre-B cells from mouse bone**

**marrow.”**

** In general figure legends and figure panels can be improved to help the reader. For example, Figure 1A, Sup figure 1b,c,d can you please add the name of the cells (pro/pre-B) on panels. Hard to follow with the legend. Many important details are omitted. Would be useful to provide the crucial details (e.g., number of animals used for each genomic assay for example) in figure panels and legends. Specific examples: Figure 1B, C, how many mice is used for these analyses? How many mice for figure 2A,C,D.*

**Response 5. We have now added more information in the figures as suggested, e.g., what is now Figure 1b-d explicitly mentions the cell types. We also added more information into other figures as suggested by this reviewer. We always used pools of mice as indicated in the Methods section. The number of replicates for all experiments, and additional statistics, are provided in Supplementary Table S1 (see Response 3). We now stress this in the Results section right at the start, writing: “We sorted cells from pooled cohorts of male young and aged mice (12-15 mice per cohort) according to Fig. 1a and generated transcriptome, chromatin accessibility (ATAC-seq), histone modification (ChIP-seq) and chromosome conformation (Hi-C) data sets to identify changes associated with the ageing phenotype (summary statistics for all datasets and replicates are provided in Additional File 2, Table S1).”**

** Figure 2D, the clustering results are confusing. What does the last cluster represent, it doesn't change with aging? Please add simple explanation next to each cluster. Authors should show the expression heatmap from all used individuals here, instead of normalizing the groups based on the median. With this change we can see the number of replicates and see whether there are outlier animals.*

**Response 6. As suggested by the reviewer, we now show the results of each single replicate experiment in what is now Figure 3c, and we have added descriptive terms for each cluster. All of the miRNAs shown in this figure were detected as being differentially expressed in pro- and/or pre-B cells upon ageing, albeit some more pronounced than others (log2 fold changes and p-values are all provided in Additional File 2, Table S5). We have rephrased the figure legend to highlight this: “Hierarchical clustering of miRNAs that are differentially expressed upon ageing in pro- and/or pre-B cells identifies groups of co-regulated miRNAs“. We have also added the following sentence to the manuscript to better describe this figure: “Differentially expressed miRNAs segregated into clusters displaying similar expression changes upon ageing, several of which also showed a modulation in their expression between pro-B and pre- B cells (Fig. 3c).”**

** Figure S3, are these p-values corrected for multiple hypothesis testing?*

**Response 7. The p-values are corrected for multiple hypothesis testing (Benjamini- Hochberg), we have now added this information to the legend.**

** Figure 3A and B, do genome browser inserts have the same scale? This is not mentioned in the legend and is a critical information.*

**Response 8. The genome browser inserts have the same scale, and we now state this in the legend. We have also added this clarification to the legend for Supplementary Figure S6.**

*I suggest authors to show the peaks as black bars under bigwig profiles for these figures instead of just an arrow that seems to be randomly pointing to this locus.*

**Response 9. We have followed this suggestion in what is now Figure 4, and have also added the peak locations in supplementary Figure S6.**

*For these figures, authors should also show examples of loci that are not differential for comparison purposes. Also, are these averages of multiple animals? Can they show the data for all biological replicates for these two loci, maybe in Sup?*

**Response 10. We now also show loci that do not exhibit changes in Additional File 1, in the new supplementary Figures S7 for ATAC-seq and H3K4me3, and S8 for H3K27me3 and H3K27ac. We have clarified in the legends for new Figure 4 and Figure S6 that the insert plots represent the median of all replicates, and present the data for single replicates in the new supplementary figures alongside the unchanged loci.**

**We have also expanded a sentence at the beginning of the Results to: “We sorted cells from pooled cohorts of male young and aged mice (12-15 mice per cohort)”, to emphasise that our analyses were performed with replicates from pooled cohorts of mice, not from individual mice.**

** FC/FC correlations should be discussed for the following: ATAC vs H3K4me3 (H3K27ac) and H3K4me3 vs H3K27ac*

**Response 11. To address this, we have added Supplementary Figure S11 to Additional File 1, and now write in Results (page 11, line 275 onwards): “In order to examine the relationship between the different types of chromatin alterations that we have analysed, we identified overlapping peaks between all pairwise combinations of ChIP-seq and ATAC-seq data within a given cell type (pro-B or pre-B), and between pro-B and pre-B cells for a given chromatin feature. We then derived correlation coefficients comparing the age-related log2 fold changes in enrichment for overlapping peaks from each pairwise combination (Additional File 1, Fig. S11, left). The highest correlation was observed for log2 fold changes in the same chromatin mark in pro-B versus pre-B cells, consistent with the correlation between age-related gene expression changes at these two developmental stages. We also observed a positive correlation between changes in ATAC-seq and H3K4me3 in ageing, but less so between either of these and H3K27ac, in line with the non-redundant nature of these chromatin features. The majority of correlation coefficients increased when considering only pairs of peaks for which at least one ranked highly (top 10%) in significance (Additional File 1, Fig. S11, right). Most strikingly, changes in H3K27me3 anti-correlate with changes in ATAC-seq, H3K4me3 and H3K27ac, suggesting coordinated changes in chromatin marks upon ageing”**

** Why does K4Me3/K27ac agrees more with DE than ATAC? Figure 4 and S7? Are all distal peaks used in these analyses? Would the results get better if only promoter peaks are considered for all assays? Need to be discussed in the paper.*

**Response 12. To address this, we now write in results (page 11, lines 269-274): “It is noteworthy that H3K4me3 and H3K27ac agree more with differential gene expression than chromatin accessibility (ATAC-seq; Fig. 5; Additional File 1, Fig. S10). This is in line with the fact that chromatin accessibility is not necessarily linked to active gene expression, but can also be found over other regulatory elements such as insulators, while H3K4me3 and H3K27ac are more strongly linked to actively transcribed genes [25, 26].”**

**We tested the reviewer’s suggestion and used only promoter peaks in both of these analyses. However, this increased the correlations only slightly for ATAC-seq; and conversely the correlations were decreased for H3K27ac, likely reflecting the localisation of this mark to enhancers. We, therefore, prefer to present the original analyses, taking into account all peaks within 1 kb of the genes, in our manuscript.**

** What is Figure 5b is showing. Correlation between emission probabilities for pairs of states? Please clarify.*

**Response 13. We apologise for not making this clear enough in our initial submission. We have now changed the legend for what is now Fig. 6, writing:**

**“a Emission parameters learnt by the Hidden Markov Model in chromHMM analysis. It shows the likelihood of emitting each of the 5 marks used in this analysis at any given chromatin state. b Transition parameters learnt by the HMM in chromHMM analysis: given the current chromatin state (at a specific 200 bp genomic region), how likely is it that the adjacent 200 bp region will be in any of the 16 inferred states. Dashed blocks show states that transit to each other more frequently suggesting a more biologically meaningful chromatin state, thus driving our clustering into 6 states: Polycomb, Bivalent, Active Promoter, Active Regulatory Regions, Insulator and Background.”**

** Did the authors generate ChromHMM states separately in old and mice? This needs further clarification and they need to show emission probabilities for old and young in 5A is this is the case.*

**Response 14. We apologise that our previous description was not clear. ChromHMM provides a setting (used, for example in the RoadMap Epigenomics project, https://www.nature.com/articles/nature14248), in which the chromatin states of different cell types can be inferred at the same time, resulting in only one emission and transition matrix of parameters. We used this setting and treated Young and Aged as different cell types.**

**We have now improved the methods section to make this clear, writing: “We took a similar approach to that described in a previous study [26] allowing us to provide both Young and Aged data (analogous to cell types in that study) in one CellMarks file. With this setting, only one Emission and one Transition parameter matrix is inferred. Based on these learnt parameters, the genomic localisation of these states is inferred separately for Young and Aged. For this, first, the genome was binned into 200 bp bins and binarised based on the pre-B cells ATAC-seq and ChIP-seq peaks using the ‘binarizeBed’ function…”**

*Also a global comparison is needed? Are there similar number of promoters, enhancers, etc in old and young? What portion of regulatory elements switch in activity with aging?*

**Response 15. To address these questions, we have generated new supplementary Figure S12, and summarise these results on page 12, line 298 onwards. We present pie charts showing the proportion of all promoters or regulatory elements (defined as the union of peaks of ATAC-seq and ChIP-seq for H3K4me3, H3K27me3, H3K27ac and CTCF) that are in each state in pre-B cells. We also show the proportion of regulatory elements that overlap with a transcription start site for each state. Finally, we present heatmaps showing the proportion of promoters and regulatory regions that are in the same state in young and aged pre-B cells, or transition from one state to another.**

*In general figure 5D does not show major changes for most genes. Can the authors speculate why?*

**Response 16. As stated above and in the Discussion section of our paper, the changes that we identified and describe are few and select, yet insightful and interesting with regard to the biology of ageing. The majority of genes with altered expression do not show differential enrichment of chromatin marks. This likely reflects in part the relatively small fold changes that we see at the transcript level, and due to this we are not surprised that we do not see many major changes in the chromatin state at these promoters. Whilst we do see global relationships between age-related gene expression changes and histone modifications (Fig. 5 and S10), these are relatively subtle. Due to the binary nature of chromHMM (at a given genomic location, a peak for a specific chromatin feature is either present or absent), such subtle changes would be unlikely to result in a complete switch between different states. This analysis thus highlights the genes that show more substantial changes, and is complemented by the other analyses we have performed that are more suitable for detecting subtle changes.**

**We have now added the following statement to the manuscript (new text in red; page 13 line 333 onwards): “Similar to the observation for the majority of upregulated genes, most of these changes were subtle, consistent with the relatively small fold changes in the expression of these genes (Fig. 2b), and the binary nature of chromHMM (at a given genomic location, a peak for a specific chromatin feature is either present or absent). However, we observed substantial changes occurring over the Irs1 promoter, where the active state transitioned to a bivalent state upon ageing (Fig. 6d); it was also identified in the genome-wide analysis, segregating in cluster 2 (Fig. 6c). Chromatin state analysis thus highlights genes that show the most profound alterations in the chromatin at their promoters.”**

** What is the difference between Figure S8a and b? Clarification needed.*

**Response 17. What is now Supplementary Figure S14a looks at promoter:promoter interactions and Figure S14b at promoter interactions with other elements other than promoters (PIR, Promoter Interacting Regions). We now make this clear on the figure panels. We have now also improved our explanation of the difference between these interaction types in the manuscript (see Response 20 below).**

** Are there significant changes in chromatin interactions at the network level with aging? Any hubs specific to age groups? Are networks less/more connected? Basic stats for these data should be discussed*.

**Response 18. Whilst a detailed analysis of gene interaction networks in young developing B cells would undoubtedly be of interest per se, this is beyond the scope of our present study. In our experience, network level changes only become apparent in systems displaying profound and widespread gene expression changes: indeed, in parallel work (Chovanec et al., manuscript in preparation) we do not find many differences in chromatin interactions at the network level even when comparing different blood cell types. Thus, we believe that it is unlikely that a comparison of networks in young and aged cells, where changes in gene regulation are comparatively subtle, would uncover significant differences.**

** Can the lost interactions in Figure 7a be due to the depth of chromatin interaction assay since there are not any other epigenomic changes in the marked regulatory elements? Can they show the 2 replicates for the promoter-capture Hi-C and Hi-C assays separately to show this is consistent across reps. Can authors test these specific interactions using 3C or 4C if they are claiming that these changes are real and consequential?*

**Response 19. We now show both replicates for these analyses over the Irs1 locus in supplementary figure S13. Since the overall quality of the data sets is high for both young and aged (as also indicated by the summary statistics of the Hi-C/ PCHi-C data in the updated Additional file 2, Table S1), we do not think that the lost interactions can be explained by differences in the quality of the data sets. Furthermore, the Promoter Capture Hi-C and Hi-C assays cross-validate changes around the Irs1 promoter. Therefore, we do not believe that additional 3C or 4C assays (utilising the same methodology in principle, but at a lower-throughput level and without the biotin pulldown step that reduces the noise levels) would add much to the analysis. Indeed, when developing PCHi-C, we validated the interactions identified using quantitative 3C (Schoenfelder et al., Genome Research 2015), 3C (Mifsud et al., Nature Genetics 2015) and DNA FISH (Schoenfelder et al., Genome Research 2015; Schoenfelder et al., Nature Genetics 2015), which demonstrated that both 3C and DNA FISH very accurately reproduced our PCHi-C results.**

** Does PIRs include promoter-promoter interactions? What is the rationale for splitting the data into two like this?*

**Response 20. To clarify this, we now write (page 15, line 370-375): “PCHi-C enriches for elements that interact with a given promoter (promoter interacting regions; PIRs), including enhancers and silencers. It also enriches for interactions between promoters, e.g., when they are located in the same transcription factory or share an enhancer. To distinguish such interactions, we analysed promoter: PIR interactions, where we specifically exclude promoter:promoter interactions, separately from promoter:promoter interactions”.**

**In addition to the different biological nature of these interactions, promoter:promoter interactions are between HindIII fragments that are both baited in our promoter capture system, which may result in different behaviour during the in-solution hybrid capture step of PCHi-C, compared to promoter:PIR interactions (where only the promoter is baited). For this reason, promoter:promoter interactions are always analysed separately from promoter:PIR interactions, in all PCHi-C publications (unless specifically stated otherwise).**

**Therefore, we now also make it clear that in what is now Fig. 7f, we decided to look at the total number of active regions contacted, without distinguishing promoter:promoter and promoter:PIR interactions, writing: “We also observed a number of cases where the promoters of upregulated genes gained connections to regions characterised by an active chromatin state (including both promoters and PIRs), while downregulated genes lost interactions with active regions”.**

** How are the p-values for Figure 6c,f are calculated, because numbers do not seem that different ? Are they testing whether there is a change? The direction also needs to be tested in these and the details of the test should be provided.*

**Response 21. We have now clarified this in the legend for what is now Figure 7c,f, writing: “FDR-adjusted p-values are based on Fisher’s exact test for independence of the proportions of gained/lost interactions across gene categories”. As can be seen from the p-values on the figures, the significance of these is not high; indeed as stated in the manuscript, the change in Figure 7f is not significant. Given that we are comparing three sets of genes, with three categories for each, we do not believe that a test for direction is appropriate.**

** How do the authors calculate A and B chromatin scores. Some details on this is needed in the methods.*

**Response 22. In the methods section, we have expanded our description of this, now writing: “The compartment signal was computed as the first principle component of the distance-and-coverage corrected interaction profile correlation matrix at 250 kb resolution, as has previously been described and shown to identify active and inactive compartments [30]. Since principle component analysis does not necessarily assign a positive value to the active compartment, H3K4me3 enrichment in young pre-B cells was compared for positive and negative compartments; positive values were then assigned to the group with high enrichment, and vice versa”.**

** Figure 6e, what is the goal of this analysis? why is there no association with epigenome and transcriptome in this analyses, despite the fact that association has been observed in global analyses. Is it due to small gene numbers tested? Please also clarify the gene numbers used in each analyses.*

**Response 23. This analysis is focused on PIRs, in contrast to the global analyses presented in earlier figures that focus on the promoters themselves. We have added this onto the figure to make it clear. We have also added the number of genes and PIRs to the legend. Whilst we detected PIRs for only approximately two thirds of differentially expressed genes, each of these interacts with multiple PIRs, comprising a total of over 300 PIRs for both up- and downregulated genes, and so we do not believe that the absence of association is driven by low numbers. This underscores the finding that while we observe re-wiring of promoters depending on changes in activity between young and aged (which, in turn, is often linked to changes in promoter chromatin) we do not find much evidence of changes in chromatin at the respective PIRs, unlike what has been described for ES cell differentiation.**

**To emphasise this, we now write in the Discussion (page 18, lines 445-450): “In contrast to the association between gene expression changes and alterations in histone modifications and chromatin accessibility over promoter regions, the chromatin at PIRs was remarkably stable. However, we found that changes in gene expression upon ageing were often accompanied by rewiring of the interactions of these promoters with other, potentially regulatory, regions of the genome. Given the absence of chromatin alterations at PIRs, it is conceivable that this rewiring is driven by chromatin modulations at the promoters themselves.”**

** Figure 5C, this analysis is confusing. First details are missing, second why do the authors believe it is useful to look at the chromatin accessibility of promoters for regions that are not promoters (i.e., insulator, Res, polycomb). This analysis should be excluded and the gene expression analyses should be concluded on the chromatin states directly instead of the clusters drived from this analysis.*

**Response 24. We apologise for any confusion in interpretation of this analysis. We believe that the approach taken in this figure to integrate the data for different chromatin states, identifying transitions rather than simply the gain or loss of an individual state, maximises the potential of our data. To clarify this analysis, we write now (page 13, line 311 onwards):**

**“To extend this analysis and identify more subtle changes that do not necessarily result in an overall switch in the state, we then calculated the difference in the total number of base pairs occupied by each state in aged compared to young pre-B cells. Whilst the majority of promoters displayed minimal changes in state occupancy upon ageing (Additional File 1, Fig. S12), we selected a subset of genes that displayed the largest magnitude changes over their promoters. In order to integrate these changes and identify groups of genes whose promoters displayed similar transitions in their chromatin state, we used unsupervised clustering. This segregated the promoters into 9 clusters displaying different patterns of state changes (Fig. 6c, top; Additional File 2, Table S10). These genes also frequently displayed modulations in gene expression consistent with their altered chromatin state (Fig. 6c, bottom).…”. We have also clarified how the subset of gene promoters was selected in the Methods section: “Genes displaying the greatest magnitudes of change in state coverage (in the top 0.5% for increased or decreased bp coverage for at least one regulatory state, excluding background) were selected, and changes in coverage were normalised by subtracting the mean change in bp coverage for a given state”.**

** Figure 5D, Authors need to compare all down/up regulated genes and whether there is more active promoter bps in aged (young) for up(down) regulated, maybe using a chi-square test.*

**Response 25. We apologise for the slight inaccuracy in our previous legend for this figure. We have now clarified, both in the legend and the manuscript, that we compared all up and downregulated genes from pre-B cells (total RNA-seq): “We next assessed chromatin state changes at the promoters of all genes showing significant age-specific expression changes in pre-B cells”. (page 13 line 324-326).**

**We do not claim that, overall, the changes of the active promoter state over up- and downregulated genes are significant. In fact, what is now Figure 6d shows that only few up- and downregulated genes (for example, the downregulated Irs1) have substantial changes at their promoter region. The figure for reviewers only shown below further illustrates this, comparing the coverage of the ‘Active promoter region’ state for each gene promoter between up- and downregulated genes. We see that there is a slight tendency for down-regulated genes to have a reduced active state coverage in aged cells, but the mean changes in active state coverage upon ageing do not show a significant difference between up- and downregulated genes (p-value = 0.16). We now make it clearer in the manuscript that most of these changes are only subtle (see Response 16).**

*Minor points:*

** Fold Change (FC) correlation between total and nascent would be useful to understand to what extent they agree.*

**Response 26. We now show this in new supplementary Figure S2, Additional file 1. In the manuscript, we have added the following (new text in red): “While ~17% (30 out of 175) of DEGs detected in total RNA were also DEGs in the nuclear RNA-seq analysis, and overall the fold changes were correlated (Additional File 1, Fig. S2), this analysis revealed many more genes showing significant age-related differential transcription” [in the nascent RNA transcriptome].**

** Venn diagrams in Figure S5b are not exciting? Is this because H3K4me3 peaks are also found at enhancers? Or did they already restrict it to promoter peaks? The authors suggest that this is a significant overlap based on chi-square p-values, but it will be more convincing to see the fold changes agree for these sites in both assays.*

**Response 27. As stated in the figure legend (now S6b): “Venn diagrams in b show overlap between differentially expressed genes (total RNA) and promoter regions with changes in H3K4me3 in pro-B cells; p-values are based on a hypergeometric test.” As the reviewer suggests, the overlaps are already restricted to promoter peaks. The pvalues are based on a hypergeometric test rather than a chi squared test, and therefore take into account the number of differentially expressed genes/enriched peaks and the total number of genes in our analyses. Whilst the numbers in the overlaps are small, given the very small number of differential genes/peaks compared to the large total number of genes, the significance of these overlaps is in fact highly significant. To clarify this we have added to the legend: “given the small number of differential genes/peaks and the large total number of genes from which they might originate, the overlaps are highly significant”. Although we do not present data directly comparing the fold change for these genes, we believe that what are now Figure 5 and supplementary Figure S10 extend and confirm this, showing a global association between changes in gene expression and H3K4me3.**

** Results from transcriptome are interesting, however, more analyses could be conducted to make sense of these changes for biology. I suggest using GSEA analyses for DEG genes both for total and nascent RNA, which might uncover pathways, gene sets that are up/down regulated as a group. Also, to further interpret the differential microRNAs authors can also incorporate their target genes using computational methods (http://www.mirdb.org/). If same pathway or genes are targeted by these, this will be extra evidence.*

**Response 28. We now provide tables of target genes for differentially expressed miRNAs in Additional File 2, Tables S13-S16, and present KEGG pathway analysis of these target genes in new Supplementary Figure S5. This analysis shows some overlap in KEGG pathway terms with the analysis of the nascent RNA and total RNA transcriptomes. As for GSEA, it is indeed a powerful method to increase the sensitivity of DE analysis, but we wanted to stay on the more stringent side, particularly given the appreciable number of associations already revealed by the conventional pathway analysis.**

** How would all the epigenomic data compare to each other if the authors put these into a single plot like, PCA or tSNE. Would be great to see this. There are some technical things to consider (like how to define peaks across assays etc), however, this can make a nice overall figure for these epigenomic assays.*

**Response 29.**

**Although a dimensionality reduction approach such as PCA, tSNE or UMAP may provide a nice visualisation, it does not take into account spatial relationships in the genome between readouts for adjacent regions and does not provide intuitive insights into differences between conditions (such as, in our case, young and old). Therefore, we instead opted to integrate chromatin information using ChromHMM analysis. In addition, when addressing an earlier point by the reviewer (see Response 11), we chose to analyse FC/FC correlations for all pairwise combinations of ATAC-seq and ChIP-seq for histone modifications, giving an overview of the relationships between age-related changes across these epigenomic datasets. Together, we believe that these analyses give deeper and more biologically meaningful insights into the associations between our data, and how they are modulated upon ageing.**

**Please note that in addition to addressing the reviewers’ comments above, we have expanded our description of the Babraham Institute Ageing colony, which we established for these studies. This is in accordance with best practice advised by the UK Home Office to increase transparency about procedures for the long-term care of aged mice, which under new guidelines has to be specifically justified.**
